# Essential nutrients and cerebral small vessel diseases: a two-sample Mendelian randomization study

**DOI:** 10.3389/fnut.2023.1172587

**Published:** 2023-06-22

**Authors:** Jiayi Li, Kailin Xia, Zhengrui Wang, Yanru Liu, Yicheng Tong, Yuwei Wang, Yumou Zhou, Linjing Zhang, Lu Tang, Dongsheng Fan, Qiong Yang

**Affiliations:** ^1^Department of Neurology, Peking University Third Hospital, Beijing, China; ^2^Peking University Health Science Center, Beijing, China; ^3^Beijing Key Laboratory of Biomarker and Translational Research in Neurodegenerative Diseases, Beijing, China; ^4^Key Laboratory for Neuroscience, National Health Commission/Ministry of Education, Peking University, Beijing, China

**Keywords:** cerebral small vessel disease, intracerebral hemorrhage, small vessel stroke, nutrient, Mendelian randomization

## Abstract

**Background:**

Previous studies have suggested a potential association between nutrients and cerebral small vessel disease (CSVD), but this association has not been fully addressed.

**Object:**

We intended to clarify the causal associations between four categories of essential nutrients (amino acids, polyunsaturated fatty acids, minerals and vitamins) and two acute manifestations of CSVD (intracerebral hemorrhage and small vessel stroke) using two-sample Mendelian randomization (MR) analysis.

**Method:**

We obtained European-based large-scale genome-wide association studies (GWASs) related to CSVD (6,255 cases and 233,058 controls) and nutrient concentrations. Causality evaluation mainly included the results of the inverse variance-weighted (IVW) method. The simple median method, the weighted median method and the MR–Egger method were adopted for sensitivity analyses.

**Results:**

For ICH or SVS, increased levels of phenylalanine (OR = 1.188, *p* < 0.001) and dihomo-gamma-linolenic acid (DGLA) (OR = 1.153, *p* = 0.001) showed risk effects, while docosapentaenoic acid (DPA) (OR = 0.501, *p* < 0.001), zinc (OR = 0.919, *p* < 0.001), and arachidonic acid (OR = 0.966, *p* = 0.007) showed protective effects. For lobar hemorrhage or SVS, AA (OR = 0.978, *p* < 0.001), zinc (OR = 0.918, *p* < 0.001), and retinol (OR = 0.753, *p* < 0.001) showed risk effects; DPA (OR = 0.682, *p* = 0.022), gamma-linolenic acid (OR = 0.120, *p* = 0.033) and 25(OH)D (OR = 0.874, *p* = 0.040) showed protective effects. For nonlobar hemorrhage or SVS, DGLA (OR = 1.088, *p* < 0.001) and phenylalanine (OR = 1.175, *p* = 0.001) showed risk effects.

**Conclusion:**

Our study analyzed the effect of nutrients on CSVD risk from a genetic perspective, with implications for CSVD prevention through nutrient supplementation.

## 1. Introduction

Cerebral small vessel disease (CSVD) is a group of clinical syndromes that affect perforated arterioles, capillaries, and venules supplying the white matter and deep structures of the brain (1). Neuroimaging findings resulting from CSVD include recent small subcortical infarcts, lacunar infarction, white matter hyperintensity (WMH) and cerebral microbleed (CMB) (2), which are considered the main vascular factors of dementia, cognitive decline, gait disturbance, mood disturbance, and stroke (3). CSVD causes small vessel stroke (SVS), which accounts for approximately 25% of ischemic stroke cases (4). Most intracerebral hemorrhages (ICHs) are also caused by CSVD. ICH and SVS are acute manifestations of CSVD caused by the rupture or occlusion of the small vessels (3).

Circulating nutrients include essential amino acids, essential polyunsaturated fatty acids, essential vitamins and essential minerals. Supplementation with essential nutrients is considered a promising preventive measure against CSVD due to their ability to maintain metabolic homeostasis and reduce oxidative stress, which plays a central role in tissue function (5). However, we only found cohort or observational studies examining dietary interventions that affect cardiovascular and cerebrovascular outcomes (6–8), but specific nutrient categories and effects were not addressed. Observational studies are more likely to be influenced by selection bias and confounders. Randomized controlled trials (RCTs) are able to overcome the limitations of observational studies and provide the highest level of evidence, but they come with a high cost and are not always possible due to ethical or other concerns (9). To overcome the limitations of RCTs, Mendelian randomization (MR) was introduced to mimic RCTs to perform causal inference. According to Mendelian Laws of Inheritance, genotypes are randomly assigned from one generation to the next generation and they are unaffected by confounding factors. Thus, we performed a MR study to decipher the causal association between essential nutrients (including essential amino acids, essential polyunsaturated fatty acids, essential minerals and essential vitamins) and CSVD (including ICH and SVS).

## 2. Materials and methods

### 2.1. Data collection and instrumental variable selection

**Figure 1:**
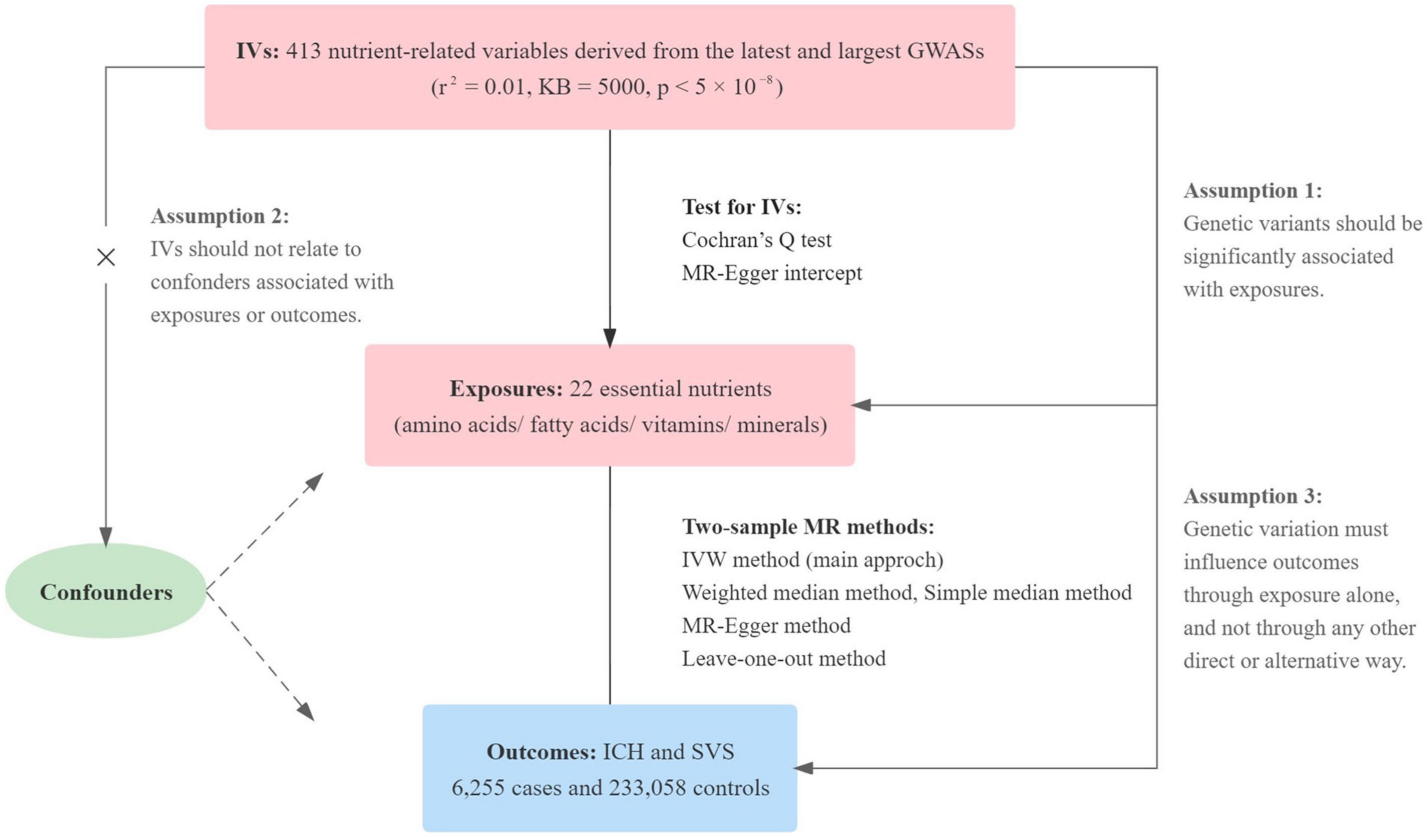
The process of our MR analyses is shown in this flow chart. Three key assumptions for our two-sample MR study. Assumption 1: The selected instrumental variables (IVs) should be significantly related to nutrients. Assumption 2: The selected IVs are not associated with other confounders. Assumption 3: There is no independent causal pathway between IVs and the outcome other than through nutrients. IVs, instrumental variables; GWASs, genome-wide association studies; MR, Mendelian randomization; IVW, inverse variance-weighted; ICH, intracerebral hemorrhage; SVS, small vessel stroke; KB, kilo base.

The design of our study was shown in Figure 1. We extracted single nucleotide polymorphisms (SNPs) for each essential nutrient (exposure) from the most recently published genome-wide association study (GWAS) with the largest sample size on PubMed. SNPs related to essential amino acids were extracted from datasets including valine, tryptophan, phenylalanine, isoleucine, leucine, lysine and methionine (10, 11). For the essential polyunsaturated fatty acids, eicosapentaenoic acid (EPA), docosapentaenoic acid (DPA), docosahexaenoic acid (DHA), arachidonic acid, dihomo-gamma-linolenic acid (DGLA), alpha-linolenic acid (ALA), gamma-linolenic acid and linoleic acid were included (10, 12, 13). Calcium (14), copper, iron (15), magnesium (16), zinc (17), and phosphorus (18) were included in the essential minerals. To assess the effects of vitamins on our outcomes, we examined vitamin A (retinol) (19), beta-carotene (20), lycopene (19), vitamin B (B6 and B12) (21, 22), vitamin C (23), vitamin D [25(OH)D] (24), and vitamin E (alpha-tocopherol) (25).

We selected the eligible SNPs as the instrumental variables (IVs) for the exposures. And we set strict selection condition on IVs: (1) SNPs should have significant associations with exposures (*p* < 5 × 10^−8^), with no linkage disequilibrium (*r*^2^ = 0.01, region size = 5,000 kb); (2) SNPs should be not rare (MAF ≥ 0.01). SNPs not found in outcome GWASs were replaced with proxies searched in the online tool SNIPA (*r*^2^ > 0.8) (26). We deleted those SNPs without available proxies.

The GWAS summary level data of outcomes were obtained from a recently published meta-analysis study, including 6,255 ICH or SVS cases and 233,058 controls from European ancestry. In this GWAS, three ICH datasets and one SVS dataset were integrated into a cross-trait dataset and generated as three outcomes, namely, ICH or SVS, lobar hemorrhage or SVS and nonlobar hemorrhage or SVS (27).

Due to the limitations of the MR methods, only exposures with at least two IVs could be used to perform the following analyses. Isoleucine, lysine, methionine, EPA, ALA, vitamin B6 and beta-carotene were removed. In total, we obtained 2-171 IVs related to 22 nutrient biomarkers (the characteristics of all the exposure SNPs were listed in Supplementary Table S1).

### 2.2. Mendelian randomization

The inverse variance-weighted (IVW) method was the main approach we used to analyze the causality; it combines the SNP-outcome effects on SNP-exposure effects by a weighted linear regression and allows the existence of overdispersion (28). We chose the multiplicative random effects model of IVW for our analysis, as it can obtain the average value of real estimates. To strengthen the robustness of our results, we used some additional sensitivity analyses including the simple median method, the weighted median method and the MR–Egger method. The result of the weighted median method is the median of a weighted empirical density function of the ratio estimates (29). It can obtain a more effective estimate when at least 50% of IVs are valid compared to the simple median method. It is sensitive when IVs are added or deleted (30). Using the MR–Egger method, we can obtain an unbiased estimate even if the SNPs have pleiotropy (31).

We used Cochran’s Q test to measure the heterogeneity of SNPs, and the MR–Egger intercept method was performed to detect horizontal pleiotropy. In Cochran’s Q test, significant heterogeneity (*p* < 0.05) indicates that there may be some genetic variants that violate key assumptions of IVs (32). For the MR–Egger regression intercept, when the y-intercept has no significant difference from the origin (*p* > 0.05), IVs can be considered to have no horizontal pleiotropy. From the principal of the MR–Egger intercept method, exposures with less than three SNPs cannot be tested for horizontal pleiotropy.

We adopted scatterplots and leave-one-out plots to describe the effect of a single SNP on the outcomes. The scatterplot showed the effect of each SNP on the exposure and outcome. The leave-one-out (LOO) plot was used to assess the influential IVs by sequentially excluding each genetic variant and recalculating the MR-IVW estimate.

The Bonferroni correction was employed for multiple-testing correction, which changed the significance level of the *p* value from 0.05 to 7.58 × 10^−4^ (*p* value = 0.05/(22*3)). All analyses were performed in R software version 4.2.0 by the “TwoSampleMR” package (version 0.5.6).

## 3. Results

We evaluated 22 possible risk factors for ICH and SVS, including four amino acids, six polyunsaturated fatty acids, six minerals and six vitamins.

### 3.1. Amino acids

**Figure 2:**
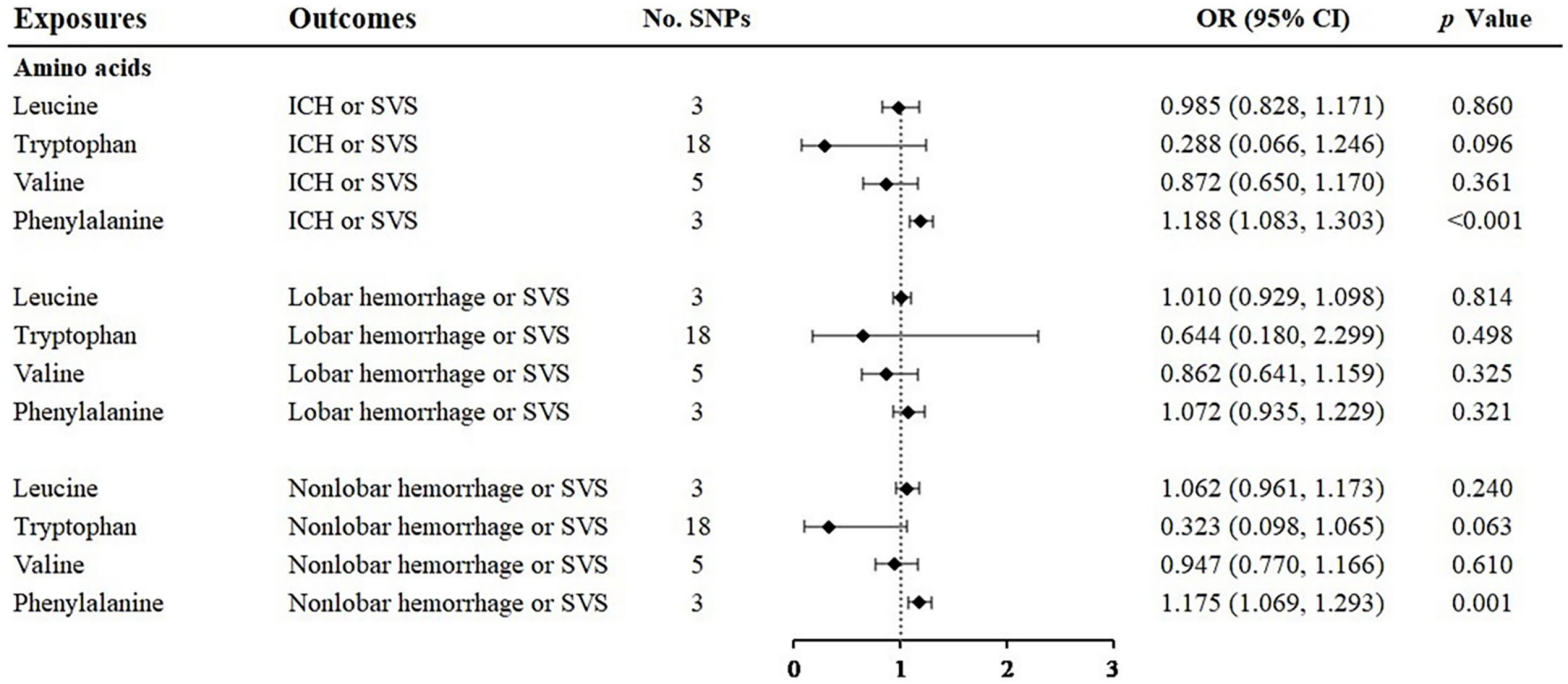
Associations of amino acids and the risk of cerebral hemorrhage or SVS using the IVW method. For the image in the figure, the black diamond represents the OR value, the straight line represents 95% CI, and the dotted line represents OR = 1. IVW, inverse variance-weighted; OR, odds ratio; 95% CI, 95% confidence interval; ICH, intracerebral hemorrhage; SVS, small vessel stroke; SNPs, single nucleotide polymorphisms.

When analyzing the relationship between amino acids and ICH or SVS, the results of the IVW method showed the risk effect of phenylalanine on ICH or SVS (OR: 1.188; 95% CI: 1.083–1.303, *p* < 0.001) and the suggestive risk effect on nonlobar hemorrhage or SVS (OR: 1.175; 95% CI: 1.069–1.293, *p* = 0.001) (Figure 2, Supplementary Figures S1, S2). The simple median, the weighted median and the MR–Egger method all illustrated similar trends (Table 1). No SNP heterogeneity was detected via Cochran’s Q test. Similarly, no horizontal pleiotropy was found by the MR–Egger intercept test (Table 2). The LOO method showed that no single SNP of phenylalanine had a significant effect on ICH or SVS (Supplementary Figure S2D). Through the LOO method, we found that the effect of phenylalanine on nonlobar hemorrhage and SVS did not remain significant after removing a SNP (rs4253238) (Supplementary Figure S2L). Leucine, tryptophan and valine were not significantly associated with the three outcomes at the genetic level (Figure 2), and the heterogeneity assessment of SNPs showed that the results were relatively reliable (Table 2).

**Table 1:** Summary of the causal effects of each trait on intracerebral hemorrhage or small vessel stroke via different Mendelian randomization methods.

Exposures	Methods	ICH or SVS			Lobar hemorrhage or SVS			Nonlobar hemorrhage or SVS		
		No. SNPs	OR(95% CI)	*p* value	No. SNPs	OR(95% CI)	*p* value	No. SNPs	OR(95% CI)	*p* value
**Amino acids**										
Leucine	Simple median	3	0.964 (0.705, 1.318)	0.818	3	0.991 (0.747, 1.316)	0.952	3	1.010 (0.805, 1.268)	0.931
Leucine	Weighted median	3	0.950 (0.718, 1.256)	0.718	3	0.988 (0.780, 1.253)	0.923	3	1.065 (0.888, 1.277)	0.497
Leucine	MR–Egger	3	0.888 (0.155, 5.079)	0.915	3	0.901 (0.192, 4.233)	0.916	3	1.641 (0.533, 5.053)	0.547
Leucine	IVW	3	0.985 (0.828, 1.171)	0.860	3	1.010 (0.929, 1.098)	0.814	3	1.062 (0.961, 1.173)	0.240
Tryptophan	Simple median	18	0.306 (0.032, 2.921)	0.304	18	0.668 (0.095, 4.712)	0.686	18	0.296 (0.066, 1.323)	0.111
Tryptophan	Weighted median	18	0.318 (0.036, 2.824)	0.304	18	0.636 (0.093, 4.367)	0.645	18	0.339 (0.077, 1.491)	0.152
Tryptophan	MR–Egger	18	0.022 (0.000, 1.584E+08)	0.746	18	0.000 (0.000, 1.983E+05)	0.451	18	2.726 (0.000, 4.02E+07)	0.907
Tryptophan	IVW	18	0.288 (0.066, 1.246)	0.096	18	0.644 (0.180, 2.299)	0.498	18	0.323 (0.098, 1.065)	0.063
Valine	Simple median	5	0.961 (0.717, 1.287)	0.788	5	0.925 (0.706, 1.211)	0.571	5	0.869 (0.690, 1.093)	0.231
Valine	Weighted median	5	0.932 (0.709, 1.227)	0.617	5	0.905 (0.717, 1.144)	0.405	5	0.947 (0.786, 1.140)	0.562
Valine	MR–Egger	5	0.556 (0.094, 3.291)	0.564	5	0.533 (0.090, 3.166)	0.538	5	0.959 (0.258, 3.559)	0.954
Valine	IVW	5	0.872 (0.650, 1.170)	0.361	5	0.862 (0.641, 1.159)	0.325	5	0.947 (0.770, 1.166)	0.610
Phenylalanine	Simple median	3	1.165 (0.829, 1.638)	0.378	3	1.133 (0.823, 1.560)	0.445	3	1.210 (0.970, 1.509)	0.091
Phenylalanine	Weighted median	3	1.183 (0.842, 1.663)	0.333	3	1.111 (0.830, 1.486)	0.479	3	1.212 (0.969, 1.517)	0.093
Phenylalanine	MR–Egger	3	1.913 (0.242, 15.147)	0.649	3	1.097 (0.176, 6.827)	0.937	3	1.406 (0.369, 5.350)	0.705
Phenylalanine	IVW	3	1.188 (1.083, 1.303)	<0.001	3	1.072 (0.935, 1.229)	0.321	3	1.175 (1.069, 1.293)	0.001
**Polyunsaturated fatty acids**										
Docosahexaenoic acid (DHA)	Simple median	5	1.248 (0.951, 1.637)	0.110	5	1.116 (0.881, 1.415)	0.363	5	1.079 (0.910, 1.278)	0.383
Docosahexaenoic acid (DHA)	Weighted median	5	1.146 (0.879, 1.494)	0.314	5	1.070 (0.862, 1.329)	0.537	5	1.026 (0.877, 1.199)	0.752
Docosahexaenoic acid (DHA)	MR–Egger	5	0.539 (0.070, 4.171)	0.595	5	0.693 (0.173, 2.773)	0.640	5	0.718 (0.295, 1.750)	0.519
Docosahexaenoic acid (DHA)	IVW	5	0.975 (0.710, 1.340)	0.878	5	0.990 (0.800, 1.225)	0.927	5	0.960 (0.835, 1.105)	0.572
Arachidonic acid (AA)	IVW	2	0.966 (0.943, 0.991)	0.007	2	0.978 (0.970, 0.986)	<0.001	2	0.983 (0.961, 1.007)	0.159
Gamma linolenic acid (GLA)	IVW	2	0.047 (0.001, 2.986)	0.149	2	0.120 (0.017, 0.840)	0.033	2	0.259 (0.011, 5.899)	0.397
Linoleic acid (LA)	Simple median	17	0.966 (0.850, 1.098)	0.594	17	0.976 (0.865, 1.102)	0.697	17	0.994 (0.916, 1.079)	0.887
Linoleic acid (LA)	Weighted median	17	0.987 (0.875, 1.113)	0.829	17	1.019 (0.907, 1.144)	0.754	17	1.003 (0.930, 1.082)	0.937
Linoleic acid (LA)	MR–Egger	17	1.097 (0.840, 1.432)	0.508	17	1.077 (0.832, 1.394)	0.582	17	1.005 (0.878, 1.150)	0.945
Linoleic acid (LA)	IVW	17	0.999 (0.892, 1.119)	0.985	17	1.007 (0.903, 1.122)	0.906	17	1.010 (0.955, 1.069)	0.726
Docosapentaenoic acid (DPA)	Simple median	3	0.468 (0.223, 0.981)	0.044	3	0.691 (0.334, 1.431)	0.320	3	1.036 (0.570, 1.882)	0.909
Docosapentaenoic acid (DPA)	Weighted median	3	0.455 (0.284, 0.730)	0.001	3	0.628 (0.411, 0.958)	0.031	3	0.780 (0.572, 1.064)	0.116
Docosapentaenoic acid (DPA)	MR–Egger	3	0.428 (0.167, 1.094)	0.327	3	0.546 (0.237, 1.261)	0.391	3	0.565 (0.287, 1.112)	0.346
Docosapentaenoic acid (DPA)	IVW	3	0.501 (0.381, 0.658)	<0.001	3	0.682 (0.491, 0.947)	0.022	3	0.768 (0.554, 1.064)	0.112
Dihomo-gamma-linolenic acid (DGLA)	IVW	2	1.153 (1.057, 1.258)	0.001	2	1.087 (0.999, 1.183)	0.053	2	1.088 (1.080, 1.096)	<0.001
**Minerals**										
Zinc	IVW	2	0.919 (0.891, 0.948)	<0.001	2	0.918 (0.913, 0.922)	<0.001	2	0.959 (0.904, 1.017)	0.159
Magnesium	Simple median	6	0.884 (0.043, 18.281)	0.936	6	1.101 (0.068, 17.764)	0.946	6	0.792 (0.088, 7.155)	0.836
Magnesium	Weighted median	6	0.832 (0.043, 16.270)	0.903	6	1.627 (0.113, 23.518)	0.721	6	0.994 (0.144, 6.877)	0.995
Magnesium	MR–Egger	6	0.139 (0.000, 4941.383)	0.731	6	3.554 (0.000, 47697.355)	0.807	6	2.895 (0.001, 5755.788)	0.797
Magnesium	IVW	6	0.542 (0.024, 12.143)	0.699	6	0.648 (0.038, 11.117)	0.765	6	0.605 (0.062, 5.935)	0.666
Iron	Simple median	3	1.127 (0.946, 1.342)	0.179	3	1.065 (0.899, 1.263)	0.467	3	1.104 (0.989, 1.233)	0.079
Iron	Weighted median	3	1.105 (0.942, 1.298)	0.221	3	1.040 (0.902, 1.198)	0.593	3	1.091 (0.985, 1.208)	0.097
Iron	MR–Egger	3	1.352 (0.767, 2.384)	0.487	3	1.307 (0.782, 2.185)	0.493	3	1.275 (0.883, 1.841)	0.419
Iron	IVW	3	1.068 (0.959, 1.189)	0.231	3	0.996 (0.878, 1.130)	0.952	3	1.073 (0.998, 1.154)	0.055
Phosphorus	Simple median	5	0.897 (0.447, 1.798)	0.759	5	1.281 (0.683, 2.404)	0.440	5	1.116 (0.655, 1.899)	0.687
Phosphorus	Weighted median	5	0.895 (0.483, 1.659)	0.726	5	1.305 (0.751, 2.268)	0.344	5	0.931 (0.619, 1.400)	0.731
Phosphorus	MR–Egger	5	0.318 (0.008, 12.174)	0.581	5	0.611 (0.014, 26.284)	0.814	5	0.323 (0.019, 5.595)	0.494
Phosphorus	IVW	5	0.956 (0.516, 1.774)	0.888	5	1.117 (0.606, 2.058)	0.723	5	0.862 (0.528, 1.409)	0.554
Calcium	Simple median	171	0.915 (0.756, 1.106)	0.358	171	1.024 (0.868, 1.206)	0.781	171	0.990 (0.875, 1.119)	0.867
Calcium	Weighted median	171	1.011 (0.791, 1.293)	0.928	171	1.041 (0.838, 1.292)	0.719	171	0.974 (0.833, 1.139)	0.744
Calcium	MR–Egger	171	0.923 (0.716, 1.189)	0.537	171	1.001 (0.806, 1.243)	0.990	171	0.921 (0.774, 1.096)	0.354
Calcium	IVW	171	0.983 (0.868, 1.112)	0.780	171	1.033 (0.931, 1.145)	0.544	171	0.998 (0.917, 1.086)	0.964
Copper	IVW	2	0.966 (0.931, 1.002)	0.062	2	0.995 (0.979, 1.011)	0.524	2	1.001 (0.981, 1.022)	0.892
**Vitamins**										
Retinol	IVW	2	0.810 (0.534, 1.231)	0.324	2	0.753 (0.718, 0.790)	<0.001	2	0.977 (0.690, 1.383)	0.894
Vitamin B12	Simple median	6	1.010 (0.840, 1.213)	0.919	6	0.929 (0.787, 1.096)	0.383	6	1.025 (0.902, 1.164)	0.707
Vitamin B12	Weighted median	6	1.045 (0.872, 1.252)	0.631	6	0.979 (0.836, 1.147)	0.792	6	1.086 (0.961, 1.227)	0.186
Vitamin B12	MR–Egger	6	1.018 (0.634, 1.632)	0.946	6	1.051 (0.663, 1.667)	0.842	6	1.047 (0.757, 1.448)	0.795
Vitamin B12	IVW	6	1.049 (0.898, 1.225)	0.547	6	0.980 (0.841, 1.143)	0.798	6	1.041 (0.936, 1.158)	0.459
Lycopene	Simple median	4	1.006 (0.903, 1.121)	0.914	4	1.038 (0.939, 1.147)	0.469	4	1.029 (0.957, 1.108)	0.440
Lycopene	Weighted median	4	1.034 (0.928, 1.151)	0.546	4	1.108 (1.003, 1.224)	0.044	4	1.053 (0.982, 1.130)	0.146
Lycopene	MR–Egger	4	1.142 (0.973, 1.339)	0.245	4	1.185 (1.029, 1.365)	0.143	4	1.094 (0.985, 1.215)	0.237
Lycopene	IVW	4	1.043 (0.969, 1.124)	0.265	4	1.059 (0.976, 1.149)	0.167	4	1.037 (0.994, 1.083)	0.096
Vitamin C	Simple median	10	1.403 (0.930, 2.117)	0.106	10	1.110 (0.821, 1.502)	0.497	10	1.236 (0.962, 1.588)	0.098
Vitamin C	Weighted median	10	0.921 (0.711, 1.192)	0.530	10	0.854 (0.679, 1.074)	0.177	10	0.916 (0.769, 1.092)	0.328
Vitamin C	MR–Egger	10	0.753 (0.464, 1.222)	0.284	10	0.792 (0.560, 1.119)	0.223	10	0.801 (0.620, 1.033)	0.126
Vitamin C	IVW	10	1.094 (0.768, 1.556)	0.619	10	0.994 (0.781, 1.266)	0.963	10	1.039 (0.846, 1.276)	0.715
25(OH)D	Simple median	70	0.716 (0.505, 1.015)	0.061	69	0.846 (0.619, 1.156)	0.293	70	0.864 (0.691, 1.079)	0.197
25(OH)D	Weighted median	70	0.868 (0.711, 1.060)	0.165	69	0.888 (0.746, 1.057)	0.180	70	0.951 (0.835, 1.084)	0.455
25(OH)D	MR–Egger	70	1.003 (0.827, 1.216)	0.976	69	0.953 (0.793, 1.145)	0.610	70	1.059 (0.930, 1.205)	0.390
25(OH)D	IVW	70	0.871 (0.755, 1.005)	0.058	69	0.874 (0.769, 0.994)	0.040	70	0.961 (0.868, 1.063)	0.436
Alpha-tocopherol	Simple median	3	1.492 (0.360, 6.193)	0.581	3	1.700 (0.536, 5.399)	0.368	3	1.058 (0.515, 2.171)	0.878
Alpha-tocopherol	Weighted median	3	1.234 (0.394, 3.866)	0.719	3	1.728 (0.598, 4.988)	0.312	3	0.987 (0.500, 1.946)	0.969
Alpha-tocopherol	MR–Egger	3	11.381 (0.005, 27250.758)	0.650	3	50.994 (0.020, 128896.032)	0.505	3	1.705 (0.028, 103.985)	0.841
Alpha-tocopherol	IVW	3	0.984 (0.415, 2.338)	0.972	3	1.072 (0.381, 3.019)	0.895	3	0.958 (0.707, 1.298)	0.782

IVW, inverse variance-weighted; OR, odds ratio; 95% CI, 95% confidence interval; ICH, intracerebral hemorrhage; SVS, small vessel stroke; SNPs, single nucleotide polymorphisms.

**Table 2:** Summary of the additional Mendelian randomization analysis for the effect of each trait on intracerebral hemorrhage or small vessel stroke.

Exposures	ICH or SVS				Lobar hemorrhage or SVS				Nonlobar hemorrhage or SVS			
	MR–Egger		Cochran’ s Q		MR–Egger		Cochran’ s Q		MR–Egger		Cochran’ s Q	
	Intercept	*p*	*Q*	*p*	Intercept	*p*	*Q*	*p*	Intercept	*p*	*Q*	*p*
**Amino acids**												
Phenylalanine	−0.038	0.728	0.208	0.901	−0.002	0.984	0.581	0.748	−0.014	0.835	0.532	0.766
Valine	0.042	0.649	7.326	0.120	0.045	0.628	9.493	0.050	−0.001	0.986	8.736	0.068
Tryptophan	0.014	0.826	12.815	0.748	0.040	0.474	12.270	0.783	−0.011	0.803	20.376	0.255
Leucine	0.010	0.925	0.892	0.640	0.011	0.907	0.265	0.876	−0.041	0.583	0.709	0.701
**Polyunsaturated fatty acids**												
Arachidonic acid (AA)	NA	NA	1.146	0.284	NA	NA	0.162	0.688	NA	NA	2.379	0.123
Docosapentaenoic acid (DPA)	0.009	0.771	0.120	0.729	0.012	0.660	1.339	0.512	0.017	0.496	2.497	0.287
Docosahexaenoic acid (DHA)	0.069	0.605	12.351	0.015	0.041	0.645	7.050	0.133	0.034	0.562	5.779	0.216
Linoleic acid (LA)	−0.015	0.459	31.388	0.012	−0.011	0.578	37.062	0.002	0.001	0.935	18.118	0.317
Gamma linolenic acid (GLA)	NA	NA	3.059	0.080	NA	NA	0.850	0.357	NA	NA	4.137	0.042
Dihomo-gamma-linolenic acid (DGLA)	NA	NA	0.834	0.361	NA	NA	0.997	0.318	NA	NA	0.014	0.907
**Minerals**												
Magnesium	0.010	0.801	8.748	0.120	−0.013	0.729	9.240	0.100	−0.012	0.691	11.317	0.045
Calcium	0.002	0.580	182.926	0.236	0.001	0.750	163.198	0.632	0.003	0.301	204.469	0.037
Phosphorus	0.050	0.589	6.486	0.166	0.027	0.770	8.050	0.090	0.045	0.541	9.752	0.045
Iron	−0.051	0.555	1.277	0.528	−0.059	0.479	2.202	0.332	−0.037	0.518	1.384	0.500
Copper	NA	NA	0.084	0.772	NA	NA	0.021	0.884	NA	NA	0.065	0.799
Zinc	NA	NA	0.060	0.806	NA	NA	0.002	0.963	NA	NA	0.521	0.471
**Vitamins**												
Retinol	NA	NA	0.216	0.642	NA	NA	0.004	0.952	NA	NA	0.361	0.548
Vitamin B12	0.005	0.899	5.774	0.329	−0.010	0.764	7.190	0.207	−0.001	0.972	6.368	0.272
Lycopene	−0.037	0.320	2.315	0.510	−0.046	0.207	3.554	0.314	−0.021	0.365	1.819	0.611
Vitamin C	0.027	0.190	20.960	0.007	0.021	0.133	17.265	0.045	0.024	0.033	23.324	0.006
25(OH)D	−0.008	0.031	65.151	0.609	−0.004	0.174	60.431	0.731	−0.005	0.026	78.496	0.203
Alpha-tocopherol	−0.081	0.646	2.125	0.346	−0.128	0.508	3.883	0.144	−0.019	0.828	0.619	0.734

ICH, intracerebral hemorrhage; SVS, small vessel stroke; NA, due to the limitation of the number of SNPs, no valid value can be obtained.

### 3.2. Polyunsaturated fatty acids

**Figure 3:**
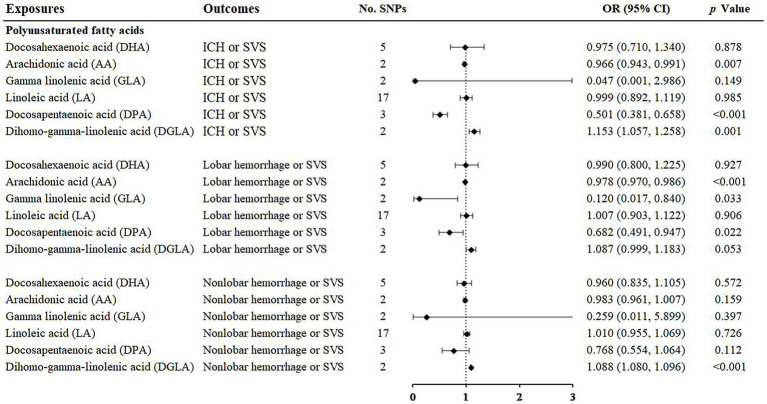
Associations of polyunsaturated fatty acids and the risk of cerebral hemorrhage or SVS using the IVW method. For the image in the figure, the black diamond represents the OR value, the straight line represents 95% CI, and the dotted line represents OR = 1. IVW, inverse variance-weighted; OR, odds ratio; 95% CI, 95% confidence interval; SVS, small vessel stroke; SNPs, single nucleotide polymorphisms.

With regard to the effect of ω3 polyunsaturated fatty acids including DPA and DHA, we found that DPA had a protective effect on ICH or SVS (OR: 0.501; 95% CI: 0.381–0.658, *p* < 0.001) and a suggestive protective effect on lobar hemorrhage or SVS (OR: 0.682; 95% CI: 0.491–0.947, *p* = 0.022) (Figure 3, Supplementary Figures S3, S4) by IVW method. The simple median method and the weighted median method provided robust evidence for the risk of DPA on ICH or SVS. The MR–Egger analysis also showed a similar trend. The weight median method demonstrated a protective trend for DPA on lobar hemorrhage or SVS (Table 1). The SNPs of DPA had no heterogeneity or horizontal pleiotropy (Table 2). DHA was not causally associated with any of the three outcomes.

For the ω6 polyunsaturated fatty acids, AA, DGLA, GLA and LA were included in our analysis. AA showed protective effects on ICH or SVS (OR: 0.966; 95% CI: 0.943–0.991, *p* = 0.007) and lobar hemorrhage or SVS (OR: 0.978; 95% CI: 0.970–0.986, *p* < 0.001). GLA had a suggestive protective relationship with lobar hemorrhage or SVS (OR: 0.120; 95% CI: 0.017–0.840, *p* = 0.033) (Figure 3, Supplementary Figures S3, S4). DGLA showed risk effects on ICH or SVS (OR: 1.153; 95% CI: 1.057–1.258, *p* = 0.001) and nonlobar hemorrhage or SVS (OR: 1.088; 95% CI: 1.080–1.096, *p* < 0.001) (Figure 3). However, there are only two available IVs for AA, as well as GLA and DGLA, which hindered the sensitivity analyses. We did not find causal association between LA and the three outcomes, which may be due to the heterogeneity in IVs of LA revealed by Cochran’s Q test (Table 2).

### 3.3. Minerals

**Figure 4:**
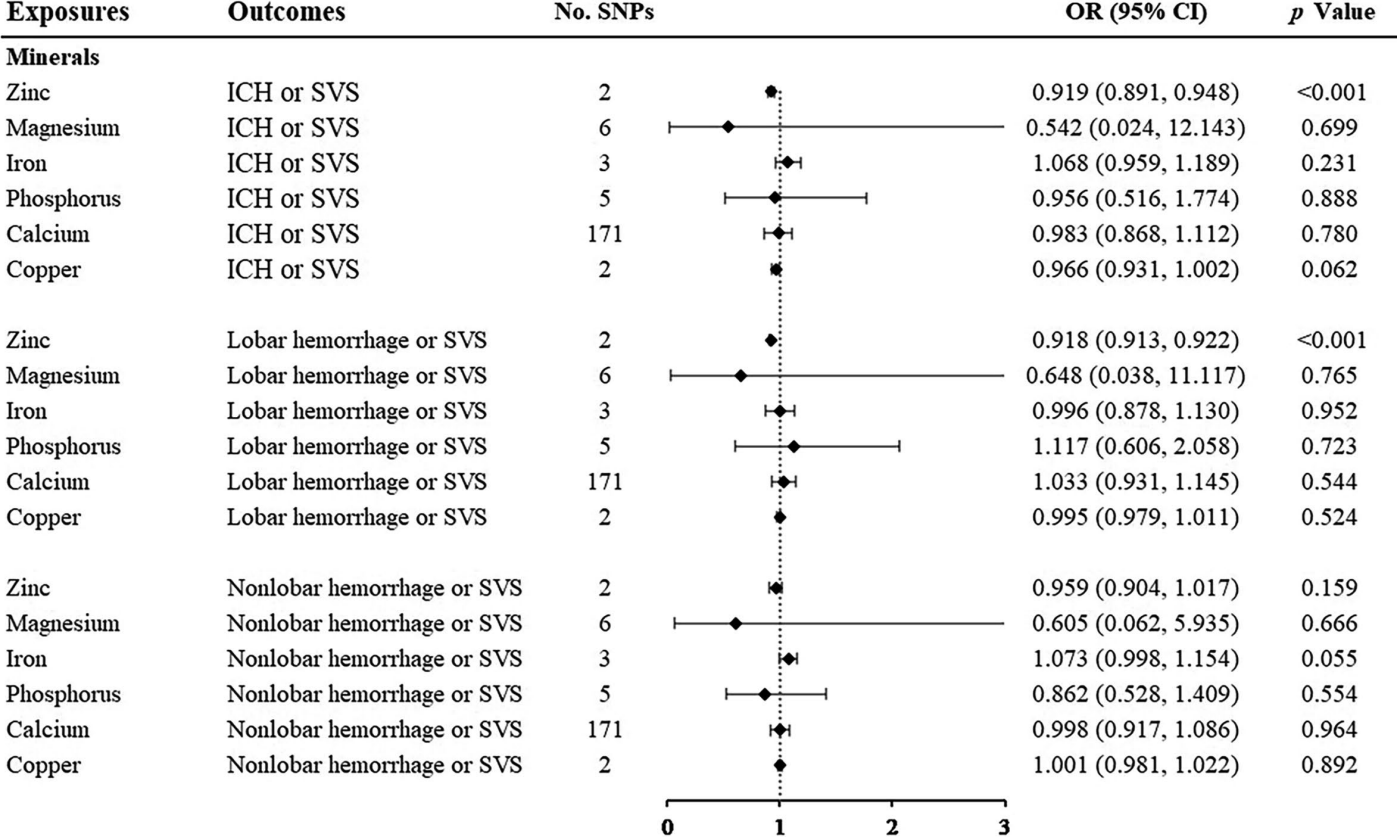
Associations of minerals and the risk of cerebral hemorrhage or SVS using the IVW method. For the image in the figure, the black diamond represents the OR value, the straight line represents 95% CI, and the dotted line represents OR = 1. IVW, inverse variance-weighted; OR, odds ratio; 95% CI, 95% confidence interval; SVS, small vessel stroke; SNPs, single nucleotide polymorphisms.

Among the minerals we were interested in, only zinc showed a protective relationship with ICH or SVS and lobar hemorrhage or SVS. For one standard deviation unit increase in the concentration of zinc, the risk of ICH or SVS decreased by 8.1% (OR: 0.919; 95% CI: 0.891–0.948, *p* < 0.001) and lobar hemorrhage or SVS decreased by 8.2% (OR: 0.918; 95% CI: 0.913–0.922, *p* < 0.001) (Figure 4, Supplementary Figures S5, S6). We could not perform the sensitivity analysis on zinc due to the limited number of IVs. None of the other minerals, namely, magnesium, iron, phosphorus, calcium, and copper, were associated with any of the three outcomes.

### 3.4. Vitamins

**Figure 5:**
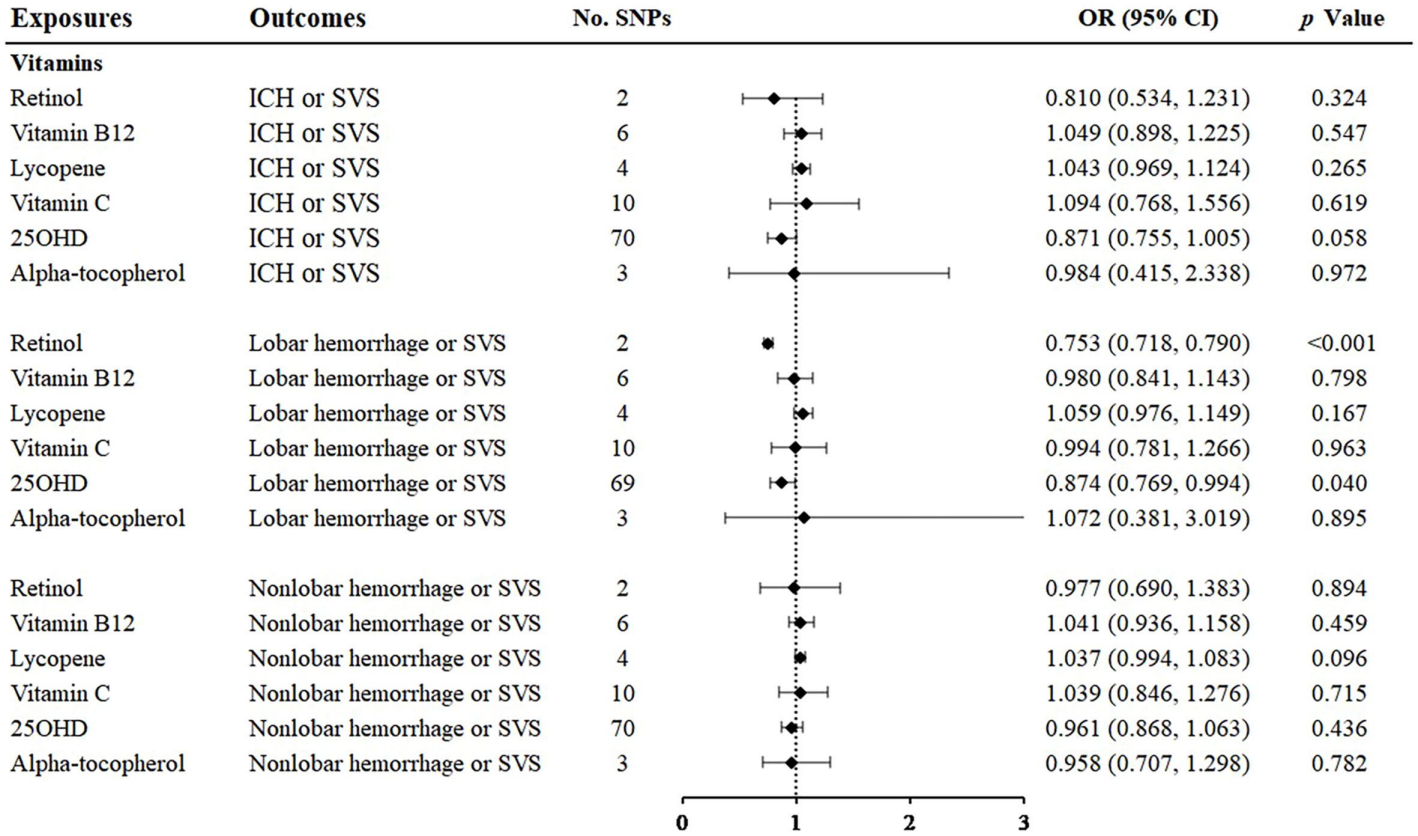
Associations of vitamins and the risk of cerebral hemorrhage or SVS using the IVW method. For the image in the figure, the black diamond represents the OR value, the straight line represents 95% CI, and the dotted line represents OR = 1. IVW, inverse variance-weighted; OR, odds ratio; 95% CI, 95% confidence interval; SVS, small vessel stroke; SNPs, single nucleotide polymorphisms.

Among the vitamins or provitamins, 25(OH)D showed a protective effect on lobar hemorrhage or SVS (OR: 0.874; 95% CI: 0.769–0.994, *p* = 0.040) (Figure 5, Supplementary Figures S7, S8). Sensitivity analyses presented similar trends (Table 1). Additional tests confirmed that 25(OH)D was not influenced by heterogeneity and horizontal pleiotropy (Table 2). Retinol showed a protective relationship with lobar hemorrhage or SVS (OR: 0.753; 95% CI: 0.718–0.790, *p* < 0.001) and sensitivity analysis could not be performed due to the limited number of IVs. None of other vitamins, namely, vitamin B12, lycopene, vitamin C, and alpha-tocopherol, were causally associated with the three outcomes.

## 4. Discussion

In our study, we performed MR analyses on essential circulating nutrients and CSVD using GWAS summary data. We found that for ICH or SVS, a higher genetically predicted level of phenylalanine was a risk factor, while DPA was a protective factor. For lobar hemorrhage or SVS, DPA and 25(OH)D were suggestive protective factors. For nonlobar hemorrhage or SVS, phenylalanine was a suggestive risk. The studies of Delgado-Lista et al. (7) and Sarfo et al. (8) have revealed some relationships between nutrients and cerebrovascular diseases, but our MR study provided more reliable evidence of CSVD by using GWAS summary data with the largest sample size by far.

Nonlobar hemorrhage is mostly caused by traditional vascular risk factors such as hypertension, and lobar hemorrhage in elderly individuals is mostly caused by cerebral amyloid angiopathy (CAA) (33). It was shown that nutrients had different effects according to the location of cerebral hemorrhage, which suggested that these nutrients may be related to different pathophysiological processes that eventually act on the brain parenchyma or cerebral blood vessels.

In our study of amino acids, phenylalanine had a hazardous predictive effect on ICH or SVS and nonlobar hemorrhage or SVS. A 12-year follow-up study of 9,584 Finnish men found that phenylalanine was associated with an increased risk of ischemic stroke and other cardiovascular events (34). A meta-analysis of seven prospective cohort studies also showed that phenylalanine was associated with a significantly increased risk of ischemic stroke (35), although ischemic stroke was not subdivided into subtypes such as SVS. There have been no previous studies of the effect of phenylalanine on the risk of ICH. Phenylalanine is an important aromatic amino acid. Excess phenylalanine can reduce cell membrane density, increase fluidity and permeability, and prompt cytotoxic effects (36). Phenylalanine may cause CSVD because it is associated with important vascular risk factors such as BMI, low-density cholesterol, systolic blood pressure, and blood glucose level (37). Phenylacetylglutamine, an intestinal microbial metabolite downstream of phenylalanine, can also increase the risk of cardiovascular disease (38). Future research is needed to explore the mechanism underlying phenylalanine and CSVD.

Regarding polyunsaturated fatty acids, previous studies based on Western populations have shown that dietary polyunsaturated fatty acid intake can reduce total cholesterol and low-density lipoprotein cholesterol, which in turn can reduce the risk of stroke (39, 40). Polyunsaturated fatty acids were defined as ω3 and ω6. The public generally recognizes that ω3 polyunsaturated fatty acid supplementation is beneficial for cardiovascular and cerebrovascular diseases. It can reduce the level of triglycerides in plasma and improve the status of coagulation, inflammation and fibrinolysis in patients (41). This protective relationship has also been confirmed by large epidemiological studies (42). However, in recent years, a growing number of meta-studies have shown that people could not obtain significant benefit for cerebrovascular outcomes from ω3 supplementation (43). Even if there is a benefit, it is only a very slight protective effect (44) and is no longer recommended clinically (45). Previous clinical studies on ω3 supplementation have varied in certain characteristics, including but not limited to dose, formulation, study population, clinical setting (primary and secondary prevention), and background therapy (46), which can all lead to bias. MR studies are known to reduce the likelihood of bias. In the ω3 category of our study, DPA showed a protective effect on ICH or SVS and a suggestive protective effect on lobar hemorrhage or SVS. There was a lack of data on the association between DPA and CSVD in the past, which should be confirmed in the future.

The circulating concentration of 25(OH)D is considered the best measurement of plasma vitamin D status (47). A meta-analysis showed that vitamin D intake and circulating vitamin D were associated with ischemic stroke but were not associated with hemorrhagic stroke (48). However, the number of original studies on hemorrhagic stroke was limited which may cause insignificant results. An RCT recruited 5,110 community participants and revealed that supplementation with vitamin D in high doses once a month did not prevent participants from cardiovascular diseases (49). One reason for this may be that vitamin D supplements have a short half-life, so the monthly dosing cycle may not achieve effective blood concentrations. Moreover, the average follow-up in the studies was only 3.3 years, and vitamin D may benefit longer-term observations. We also found some previous MR studies about the association between 25(OH)D and stroke. One study found that higher plasma 25(OH)D concentrations were not associated with ischemic stroke and its subtypes, including SVS. However, there was only 6 SNPs associated with 25OHD concentrations in this study, which was 69 in our study. Another previous MR study showed that vitamin D was a protective factor of ICH (50), which was consistent with our results. In addition, 25(OH)D was proven to correlate inversely with the neuroimaging markers of CSVD, including lacunar infarction, WMH and deep CMB (51). The outcome of our study was the cross trait of ICH and SVS, which can reveal the comprehensive associations between 25(OH)D and CSVD. Thus, our result may be more powerful due to the larger sample size of GWAS and larger number of SNPs. Vitamin D plays an important role in the occurrence of various cardiometabolic features, including diabetes (52), metabolic syndrome (53), renin-angiotensin system activation (54) and inhibition of the production of some inflammatory factors (55). These mechanisms supported the biological plausibility for our research.

We also focused on vitamin B12. Vitamin B12 is generally recognized to have a protective effect on cerebrovascular risk through homocysteine metabolic pathways, but the results were nonsignificant in our analyses. This may be due to the limited effect of vitamin B12 supplementation on lowering serum homocysteine levels (56). Additionally, a RCT study showed that vitamin B12 did not appear to be as effective as placebo in reducing the incidence of major vascular events (57). Moreover, an MR study showed that vitamin B12 was not significantly related to SVS (58). In summary, vitamin B12 supplementation may not protect people from CSVD.

In addition to focusing on the effects of nutrients on CSVD, we considered the effects of nutrients that exhibit a protective trend toward lobar hemorrhage or SVS on cerebral amyloid angiopathy (CAA), including DPA and vitamin D. CAA is generally caused by the deposition of amyloid proteins in cerebral blood vessels. For fatty acids, their aggregates can cobind to peptide oligomers in the early stages of amyloid fibrosis and inhibit the formation of amyloids. DPA may use this pathway to prevent lobar hemorrhage. Vitamin D plays an important role in the maintenance of calcium homeostasis, the reduction in calcium influx caused by amyloid deposition and the reduction in neuronal damage (59). This suggests that these nutrients might affect the occurrence of CAA by influencing pathophysiological processes similar to amyloid deposition in AD.

Our research used cross-trait GWAS data of ICH and SVS to avoid the problem of reduced efficiency due to the small sample size of ICH GWAS data alone (60). This method effectively utilized the large-scale SVS GWAS data and was able to better reflect the comprehensive effect of exposures on the common features of CSVD. Previous prospective cohort studies focusing on nutrients and CSVD have been hampered due to the difficulties in collecting accurate blood concentrations of single nutrients, individual fluctuations in blood drug levels, and the need for a long observation period after the intervention. Our MR study avoided these difficulties and provided high-quality evidence for clinical studies in the future.

Nevertheless, our study had some limitations. As the GWAS outcomes were cross-featured, it was not possible to distinguish exposures that were independently associated with ICH and SVS, which obscured the contradictions between ICH and SVS. Moreover, some nutrients including AA, GLA, DGLA, zinc, and retinol have limited number of IVs, which may reduce the statistical power. However, the GWAS data we used were the largest by far and it may provide some insight into this area. Finally, in terms of the interpretation of the results, our analyses reflected the impact of lifetime nutrient accumulation, and the short-term efficacy of nutrient supplementation should be interpreted cautiously. Further validations by clinical studies are still needed.

## 5. Conclusion

Our results showed that for ICH or SVS, a higher genetically predicted level of phenylalanine was a risk factor, while DPA was a protective factor. For lobar hemorrhage or SVS, DPA and 25(OH)D were suggestive protective factors. For nonlobar hemorrhage or SVS, phenylalanine was a suggestive risk factor. Our study analyzed the effects of essential nutrients on the risk of ICH and SVS from the perspective of gene–environment interactions, which could benefit intervention strategies for CSVD in the general population.

## Data availability statement

The original contributions presented in the study are included in the article/Supplementary material, further inquiries can be directed to the corresponding authors.

## Author contributions

KX: data curation and methodology. JL: formal analysis. DF and QY: supervision. ZW, YL, YT, YW, YZ, LZ, and LT: validation. JL and KX: writing—original draft. JL, KX, LT, QY, and DF: writing—review and editing. All authors contributed to the article and approved the submitted version.

## Funding

This research was funded by the National Natural Science Foundation of China (grant number 81901204) and the Beijing Municipal Science and Technology Commission (grant number D141100000114005).

## Conflict of interest

The authors declare that the research was conducted in the absence of any commercial or financial relationships that could be construed as a potential conflict of interest.

## Publisher’s note

All claims expressed in this article are solely those of the authors and do not necessarily represent those of their affiliated organizations, or those of the publisher, the editors and the reviewers. Any product that may be evaluated in this article, or claim that may be made by its manufacturer, is not guaranteed or endorsed by the publisher.

## Figures and Tables

**Table:** 

ALA	Alpha-linolenic acid
CAA	Cerebral amyloid angiopathy
CI	Confidence interval
CMB	Cerebral microbleed
CSVD	Cerebral small vessel disease
DGLA	Dihomo-gamma-linolenic acid
DHA	Docosahexaenoic acid
DPA	Docosapentaenoic acid
EPA	Eicosapentaenoic acid
GWAS	Genome-wide association study
ICH	Intracerebral hemorrhage
IV	Instrumental variable
IVW	Inverse variance-weighted
KB	Kilo base
LOO	Leave-one-out
MR	Mendelian randomization
OR	Odds ratio
RCT	Randomized controlled trial
SNP	Single nucleotide polymorphism
SVS	Small vessel stroke
WMH	White matter hyperintensity

## References

[1] Wardlaw JM Smith C Dichgans M. Small vessel disease: mechanisms and clinical implications. Lancet Neurol. (2019) 18:684–96. doi: 10.1016/S1474-4422(19)30079-1 31097385

[2] Wardlaw JM Smith EE Biessels GJ Cordonnier C Fazekas F Frayne R. Neuroimaging standards for research into small vessel disease and its contribution to ageing and neurodegeneration. Lancet Neurol. (2013) 12:822–38. doi: 10.1016/S1474-4422(13)70124-8, PMID: 23867200 PMC3714437

[3] Ter Telgte A van Leijsen EMC Wiegertjes K Klijn CJM Tuladhar AM de Leeuw FE. Cerebral small vessel disease: from a focal to a global perspective. Nat Rev Neurol. (2018) 14:387–98. doi: 10.1038/s41582-018-0014-y, PMID: 29802354

[4] Traylor M Malik R Nalls MA Cotlarciuc I Radmanesh F Thorleifsson G. Genetic variation at 16q24.2 is associated with small vessel stroke. Ann Neurol. (2017) 81:383–94. doi: 10.1002/ana.24840, PMID: 27997041 PMC5366092

[5] Shenkin A. The key role of micronutrients. Clin Nutr. (2006) 25:1–13. doi: 10.1016/j.clnu.2005.11.006 16376462

[6] Nassir C Ghazali MM Hashim S Idris NS Yuen LS Hui WJ. Diets and cellular-derived microparticles: weighing a plausible link with cerebral small vessel disease. Front Cardiovasc Med. (2021) 8:632131. doi: 10.3389/fcvm.2021.632131, PMID: 33718454 PMC7943466

[7] Delgado-Lista J Alcala-Diaz JF Torres-Peña JD Quintana-Navarro GM Fuentes F Garcia-Rios A. Long-term secondary prevention of cardiovascular disease with a Mediterranean diet and a low-fat diet (CORDIOPREV): a randomised controlled trial. Lancet. (2022) 399:1876–85. doi: 10.1016/S0140-6736(22)00122-2 35525255

[8] Sarfo FS Ovbiagele B Gebregziabher M Akpa O Akpalu A Wahab K. Unraveling the risk factors for spontaneous intracerebral hemorrhage among west Africans. Neurology. (2020) 94:e998–e1012. doi: 10.1212/WNL.0000000000009056, PMID: 32075893 PMC7238923

[9] Lee K Lim CY. Mendelian randomization analysis in observational epidemiology. J Lipid Atheroscler. (2019) 8:67–77. doi: 10.12997/jla.2019.8.2.67, PMID: 32821701 PMC7379124

[10] Kettunen J Demirkan A Würtz P Draisma HHM Haller T Rawal R. Genome-wide study for circulating metabolites identifies 62 loci and reveals novel systemic effects of LPA. Nat Commun. (2016) 7:11122. doi: 10.1038/ncomms11122, PMID: 27005778 PMC4814583

[11] The Multiple Tissue Human Expression Resource (MuTHER) Consortium Shin SY Fauman EB Petersen AK Krumsiek J Santos R. An atlas of genetic influences on human blood metabolites. Nat Genet. (2014) 46:543–50. doi: 10.1038/ng.2982, PMID: 24816252 PMC4064254

[12] Guan W Steffen BT Lemaitre RN Wu JHY Tanaka T Manichaikul A. Genome-wide association study of plasma N6 polyunsaturated fatty acids within the cohorts for heart and aging research in genomic epidemiology consortium. Circ Cardiovasc Genet. (2014) 7:321–31. doi: 10.1161/CIRCGENETICS.113.000208, PMID: 24823311 PMC4123862

[13] Lemaitre RN Tanaka T Tang W Manichaikul A Foy M Kabagambe EK. Genetic loci associated with plasma phospholipid n-3 fatty acids: a meta-analysis of genome-wide association studies from the CHARGE consortium. PLoS Genet. (2011) 7:e1002193. doi: 10.1371/journal.pgen.1002193, PMID: 21829377 PMC3145614

[14] Sinnott-Armstrong N Tanigawa Y Amar D Mars N Benner C Aguirre M. Genetics of 35 blood and urine biomarkers in the UK biobank. Nat Genet. (2021) 53:185–94. doi: 10.1038/s41588-020-00757-z, PMID: 33462484 PMC7867639

[15] Benyamin B Esko T Ried JS Radhakrishnan A Vermeulen SH Traglia M. Novel loci affecting iron homeostasis and their effects in individuals at risk for hemochromatosis. Nat Commun. (2014) 5:4926. doi: 10.1038/ncomms5926, PMID: 25352340 PMC4215164

[16] Meyer TE Verwoert GC Hwang SJ Glazer NL Smith AV van Rooij FJ. Genome-wide association studies of serum magnesium, potassium, and sodium concentrations identify six loci influencing serum magnesium levels. PLoS Genet. (2010) 6:e1001045. doi: 10.1371/journal.pgen.1001045, PMID: 20700443 PMC2916845

[17] Evans DM Zhu G Dy V Heath AC Madden PA Kemp JP. Genome-wide association study identifies loci affecting blood copper, selenium and zinc. Hum Mol Genet. (2013) 22:3998–4006. doi: 10.1093/hmg/ddt239, PMID: 23720494 PMC3766178

[18] Kestenbaum B Glazer NL Köttgen A Felix JF Hwang S-J Liu Y. Common genetic variants associate with serum phosphorus concentration. J Am Soc Nephrol. (2010) 21:1223–32. doi: 10.1681/ASN.2009111104, PMID: 20558539 PMC3152230

[19] D’Adamo CR D’Urso A Ryan KA Yerges-Armstrong LM Semba RD Steinle NI. A common variant in the SETD7 gene predicts serum lycopene concentrations. Nutrients. (2016) 8:82. doi: 10.3390/nu8020082, PMID: 26861389 PMC4772045

[20] Ferrucci L Perry JR Matteini A Perola M Tanaka T Silander K. Common variation in the beta-carotene 15,15′-monooxygenase 1 gene affects circulating levels of carotenoids: a genome-wide association study. Am J Hum Genet. (2009) 84:123–33. doi: 10.1016/j.ajhg.2008.12.019, PMID: 19185284 PMC2668002

[21] Hazra A Kraft P Lazarus R Chen C Chanock SJ Jacques P. Genome-wide significant predictors of metabolites in the one-carbon metabolism pathway. Hum Mol Genet. (2009) 18:4677–87. doi: 10.1093/hmg/ddp428, PMID: 19744961 PMC2773275

[22] Grarup N Sulem P Sandholt CH Thorleifsson G Ahluwalia TS Steinthorsdottir V. Genetic architecture of vitamin B12 and folate levels uncovered applying deeply sequenced large datasets. PLoS Genet. (2013) 9:e1003530. doi: 10.1371/journal.pgen.1003530, PMID: 23754956 PMC3674994

[23] Zheng J-S Luan J’a Sofianopoulou E Imamura F Stewart ID Day FR. Plasma vitamin C and type 2 diabetes: genome-wide association study and Mendelian randomization analysis in European populations. Diabetes Care. (2021) 44:98–106. doi: 10.2337/dc20-1328, PMID: 33203707 PMC7783939

[24] Manousaki D Mitchell R Dudding T Haworth S Harroud A Forgetta V. Genome-wide association study for vitamin D levels reveals 69 independent loci. Am J Hum Genet. (2020) 106:327–37. doi: 10.1016/j.ajhg.2020.01.017, PMID: 32059762 PMC7058824

[25] Major JM Yu K Wheeler W Zhang H Cornelis MC Wright ME. Genome-wide association study identifies common variants associated with circulating vitamin E levels. Hum Mol Genet. (2011) 20:3876–83. doi: 10.1093/hmg/ddr296, PMID: 21729881 PMC3168288

[26] Matthias A. SNiPA: an interactive, genetic variant-centered annotation browser. Bioinformatics (Oxford, England). (2015) 31:1334–6. doi: 10.1093/bioinformatics/btu779 25431330 PMC4393511

[27] Chung J Marini S Pera J Norrving B Jimenez-Conde J Roquer J. Genome-wide association study of cerebral small vessel disease reveals established and novel loci. Brain. (2019) 142:3176–89. doi: 10.1093/brain/awz233 31430377 PMC6763741

[28] Burgess S Bowden J Fall T Ingelsson E Thompson SG. Sensitivity analyses for robust causal inference from Mendelian randomization analyses with multiple genetic variants. Epidemiology. (2017) 28:30–42. doi: 10.1097/EDE.0000000000000559, PMID: 27749700 PMC5133381

[29] Zheng J Baird D Borges MC Bowden J Hemani G Haycock P. Recent developments in Mendelian randomization studies. Curr Epidemiol Rep. (2017) 4:330–45. doi: 10.1007/s40471-017-0128-6, PMID: 29226067 PMC5711966

[30] Bowden J Davey Smith G Haycock PC Burgess S. Consistent estimation in Mendelian randomization with some invalid instruments using a weighted median estimator. Genet Epidemiol. (2016) 40:304–14. doi: 10.1002/gepi.21965, PMID: 27061298 PMC4849733

[31] Bowden J Davey Smith G Burgess S. Mendelian randomization with invalid instruments: effect estimation and bias detection through egger regression. Int J Epidemiol. (2015) 44:512–25. doi: 10.1093/ije/dyv080, PMID: 26050253 PMC4469799

[32] Bowden J del Greco M F Minelli C Zhao Q Lawlor DA Sheehan NA. Improving the accuracy of two-sample summary-data Mendelian randomization: moving beyond the NOME assumption. Int J Epidemiol. (2019) 48:728–42. doi: 10.1093/ije/dyy258, PMID: 30561657 PMC6659376

[33] Jolink WMT Wiegertjes K Rinkel GJE Algra A de Leeuw FE Klijn CJM. Location-specific risk factors for intracerebral hemorrhage: systematic review and meta-analysis. Neurology. (2020) 95:e1807–18. doi: 10.1212/WNL.0000000000010418, PMID: 32690784

[34] Jauhiainen R Vangipurapu J Laakso A Kuulasmaa T Kuusisto J Laakso M. The association of 9 amino acids with cardiovascular events in Finnish men in a 12-year follow-up study. J Clin Endocrinol Metabol. (2021) 106:3448–54. doi: 10.1210/clinem/dgab562, PMID: 34346487 PMC8634085

[35] Vojinovic D Kalaoja M Trompet S Fischer K Shipley MJ Li S. Association of circulating metabolites in plasma or serum and risk of stroke: Meta-analysis from seven prospective cohorts. Neurology. (2020) 96:e1110–23. doi: 10.1212/WNL.0000000000011236, PMID: 33268560 PMC8055347

[36] Erimban S Daschakraborty S. How does excess phenylalanine affect the packing density and fluidity of a lipid membrane? Phys Chem Chem Phys. (2021) 23:27294–303. doi: 10.1039/D1CP05004D, PMID: 34850794

[37] Vangipurapu J Stancáková A Smith U Kuusisto J Laakso M. Nine amino acids are associated with decreased insulin secretion and elevated glucose levels in a 7.4-year follow-up study of 5,181 Finnish men. Diabetes. (2019) 68:1353–8. doi: 10.2337/db18-1076, PMID: 30885989

[38] Nemet I Saha PP Gupta N Zhu W Romano KA Skye SM. A cardiovascular disease-linked gut microbial metabolite acts via adrenergic receptors. Cells. (2020) 180:862–77.e22. doi: 10.1016/j.cell.2020.02.016, PMID: 32142679 PMC7402401

[39] Sacks FM Lichtenstein AH Wu JHY Appel LJ Creager MA Kris-Etherton PM. Dietary fats and cardiovascular disease: a presidential advisory from the American Heart Association. Circulation. (2017) 136:e1–e23. doi: 10.1161/CIR.0000000000000510, PMID: 28620111

[40] Schwab U Lauritzen L Tholstrup T Haldorsson TI Riserus U Uusitupa M. Effect of the amount and type of dietary fat on cardiometabolic risk factors and risk of developing type 2 diabetes, cardiovascular diseases, and cancer: a systematic review. Nutr Res. (2014) 58:25145. doi: 10.3402/fnr.v58.25145, PMID: 25045347 PMC4095759

[41] Derosa G Maffioli P D'Angelo A Salvadeo S Ferrari I Fogari E. Effects of long chain ω-3 fatty acids on metalloproteinases and their inhibitors in combined dyslipidemia patients. Expert Opin Pharmacother. (2009) 10:1239–47. doi: 10.1517/14656560902865601, PMID: 19397392

[42] Kris-Etherton PM Harris WS Appel LJ. Fish consumption, fish oil, omega-3 fatty acids, and cardiovascular disease. Circulation. (2002) 106:2747–57. doi: 10.1161/01.CIR.0000038493.65177.94 12438303

[43] Zheng T Zhao J Wang Y Liu W Wang Z Shang Y. The limited effect of omega-3 polyunsaturated fatty acids on cardiovascular risk in patients with impaired glucose metabolism: a meta-analysis. Clin Biochem. (2014) 47:369–77. doi: 10.1016/j.clinbiochem.2013.11.025, PMID: 24342751

[44] Abdelhamid AS Brown TJ Brainard JS Biswas P Thorpe GC Moore HJ. Omega-3 fatty acids for the primary and secondary prevention of cardiovascular disease. Cochrane Database Syst Rev. (2020) 2020:CD003177. doi: 10.1002/14651858.CD003177.pub5 PMC7049091 32114706

[45] Sheikh O Vande Hei AG Battisha A Hammad T Pham S Chilton R. Cardiovascular, electrophysiologic, and hematologic effects of omega-3 fatty acids beyond reducing hypertriglyceridemia: as it pertains to the recently published REDUCE-IT trial. Cardiovasc Diabetol. (2019) 18:84. doi: 10.1186/s12933-019-0887-0, PMID: 31234885 PMC6591979

[46] Siscovick DS Barringer TA Fretts AM Wu JH Lichtenstein AH Costello RB. Omega-3 polyunsaturated fatty acid (fish oil) supplementation and the prevention of clinical cardiovascular disease: a science advisory from the American Heart Association. Circulation. (2017) 135:e867–84. doi: 10.1161/CIR.0000000000000482, PMID: 28289069 PMC6903779

[47] Canada H. Dietary reference intakes. Nutr Rev. (1997) 55:319. 9329268 10.1111/j.1753-4887.1997.tb01621.x

[48] Zhou R Wang M Huang H Li W Hu Y Wu T. Lower vitamin D status is associated with an increased risk of ischemic stroke: a systematic review and Meta-analysis. Nutrients. (2018) 10:277. doi: 10.3390/nu10030277, PMID: 29495586 PMC5872695

[49] Scragg R Stewart AW Waayer D Lawes CMM Toop L Sluyter J. Effect of monthly high-dose vitamin D supplementation on cardiovascular disease in the vitamin D assessment study: a randomized clinical trial. JAMA Cardiol. (2017) 2:608–16. doi: 10.1001/jamacardio.2017.0175, PMID: 28384800 PMC5815022

[50] Szejko N Acosta JN Both CP Leasure A Matouk C Sansing L. Genetically-Proxied levels of vitamin D and risk of intracerebral hemorrhage. J Am Heart Assoc. (2022) 11:e024141. doi: 10.1161/JAHA.121.024141, PMID: 35730641 PMC9333362

[51] Chung PW Park KY Kim JM Shin DW Park MS Chung YJ. 25-hydroxyvitamin D status is associated with chronic cerebral small vessel disease. Stroke. (2015) 46:248–51. doi: 10.1161/STROKEAHA.114.007706, PMID: 25424481

[52] Dadrass A Mohamadzadeh Salamat K Hamidi K Azizbeigi K. Anti-inflammatory effects of vitamin D and resistance training in men with type 2 diabetes mellitus and vitamin D deficiency: a randomized, double-blinded, placebo-controlled clinical trial. J Diabetes Metab Disord. (2019) 18:323–31. doi: 10.1007/s40200-019-00416-z, PMID: 31890657 PMC6914746

[53] Park JE Pichiah PBT Cha YS. Vitamin D and metabolic diseases: growing roles of vitamin D. J Obes Metab Syndr. (2018) 27:223–32. doi: 10.7570/jomes.2018.27.4.223, PMID: 31089567 PMC6513299

[54] Cui C Xu P Li G Qiao Y Han W Geng C. Vitamin D receptor activation regulates microglia polarization and oxidative stress in spontaneously hypertensive rats and angiotensin II-exposed microglial cells: role of renin-angiotensin system. Redox Biol. (2019) 26:101295. doi: 10.1016/j.redox.2019.101295, PMID: 31421410 PMC6831892

[55] Krivoy A Satz J Hornfeld SH Bar L Gaughran F Shoval G. Low levels of serum vitamin D in clozapine-treated schizophrenia patients are associated with high levels of the proinflammatory cytokine IL-6. Int Clin Psychopharmacol. (2020) 35:208–13. doi: 10.1097/YIC.0000000000000303, PMID: 31913874

[56] Collaboration HLT. Lowering blood homocysteine with folic acid based supplements: meta-analysis of randomised trials. Homocysteine lowering trialists' collaboration. BMJ. (1998) 316:894–8. doi: 10.1136/bmj.316.7135.894, PMID: 9569395 PMC28491

[57] VITATOPS Trial Study Group. B vitamins in patients with recent transient ischaemic attack or stroke in the VITAmins TO prevent stroke (VITATOPS) trial: a randomised, double-blind, parallel, placebo-controlled trial. Lancet Neurol. (2010) 9:855–65. doi: 10.1016/S1474-4422(10)70187-3, PMID: 20688574

[58] Larsson SC Traylor M Markus HS. Homocysteine and small vessel stroke: a mendelian randomization analysis. Ann Neurol. (2019) 85:495–501. doi: 10.1002/ana.25440, PMID: 30785218 PMC6594149

[59] Przybelski RJ Binkley NC. Is vitamin D important for preserving cognition? A positive correlation of serum 25-hydroxyvitamin D concentration with cognitive function. Arch Biochem Biophys. (2007) 460:202–5. doi: 10.1016/j.abb.2006.12.018, PMID: 17258168

[60] Woo D Falcone GJ Devan WJ Brown WM Biffi A Howard TD. Meta-analysis of genome-wide association studies identifies 1q22 as a susceptibility locus for intracerebral hemorrhage. Am J Hum Genet. (2014) 94:511–21. doi: 10.1016/j.ajhg.2014.02.012, PMID: 24656865 PMC3980413

