# Supplementary material for: Essential nutrients and cerebral small vessel diseases: a two-sample Mendelian randomization study
Source: Front Nutr. 2023 Jun 22;10:1172587. doi: 10.3389/fnut.2023.1172587 (PMC10325681; doi:10.3389/fnut.2023.1172587)
Supplement: Supplementary file 1 [file Data_Sheet_1.docx]

Supplementary Material

Essential Nutrients and Cerebral Small Vessel Diseases: A Two-Sample Mendelian Randomization Study

Jiayi Li ^†^, Kailin Xia ^†^ , Zhengrui Wang, Yanru Liu, Yicheng Tong, Yuwei Wang, Yumou Zhou, Linjing Zhang, Lu Tang, Dongsheng Fan * and Qiong Yang *

^†^These authors contributed equally to this work and share first authorship.

*** Correspondence:**

[Dongsheng](mailto:email@uni.eduDongsheng) Fan: [dsfan2010@aliyun.com](mailto:dsfan2010@aliyun.com); Qiong Yang: yangqiongputh@126.com

# Supplementary Table

**Supplementary Table S1.** Characteristics of instrumental variables (IVs)

| SNP | A1 | A2 | A1FREQ | BETA | SE | P value | Traits | N |
| --- | --- | --- | --- | --- | --- | --- | --- | --- |
| rs1011468 | A | G | 0.475748 | -0.013795 | 0.001946 | 1.35E-12 | 25OHD | 443734 |
| rs10127775 | T | A | 0.604643 | 0.011801 | 0.001997 | 3.43E-09 | 25OHD | 443734 |
| rs10426 | A | G | 0.213371 | 0.025226 | 0.002382 | 3.31E-26 | 25OHD | 443734 |
| rs1047891 | A | C | 0.316449 | -0.014168 | 0.002088 | 1.16E-11 | 25OHD | 443734 |
| rs10500209 | C | T | 0.282129 | -0.013417 | 0.002169 | 6.18E-10 | 25OHD | 443734 |
| rs10818769 | G | C | 0.856966 | -0.016972 | 0.00287 | 3.35E-09 | 25OHD | 443734 |
| rs10832289 | T | A | 0.410173 | -0.068522 | 0.001965 | 2.03E-266 | 25OHD | 443734 |
| rs10859995 | C | T | 0.581322 | -0.039396 | 0.001971 | 7.03E-89 | 25OHD | 443734 |
| rs10887718 | T | C | 0.527362 | -0.012476 | 0.001946 | 1.44E-10 | 25OHD | 443734 |
| rs111529171 | C | G | 0.216376 | -0.015488 | 0.002369 | 6.24E-11 | 25OHD | 443734 |
| rs11264360 | A | T | 0.24292 | 0.018008 | 0.002286 | 3.34E-15 | 25OHD | 443734 |
| rs1149605 | C | T | 0.171453 | 0.01928 | 0.002577 | 7.34E-14 | 25OHD | 443734 |
| rs11723621 | G | A | 0.291123 | -0.18693 | 0.002121 | 2.903E-1689 | 25OHD | 443734 |
| rs117913124 | A | G | 0.027583 | -0.354126 | 0.005937 | 1.653E-775 | 25OHD | 443734 |
| rs12123821 | T | C | 0.04793 | 0.074472 | 0.004583 | 2.25E-59 | 25OHD | 443734 |
| rs1229984 | C | T | 0.973099 | -0.046854 | 0.006481 | 4.85E-13 | 25OHD | 443734 |
| rs12317268 | G | A | 0.151589 | -0.018528 | 0.002717 | 9.15E-12 | 25OHD | 443734 |
| rs12803256 | G | A | 0.770616 | 0.100325 | 0.002325 | 8.599E-407 | 25OHD | 443734 |
| rs12997242 | A | G | 0.437687 | -0.012512 | 0.001972 | 2.23E-10 | 25OHD | 443734 |
| rs157595 | G | A | 0.614127 | -0.015581 | 0.00205 | 2.95E-14 | 25OHD | 443734 |
| rs17765311 | C | A | 0.344611 | -0.01515 | 0.002047 | 1.35E-13 | 25OHD | 443734 |
| rs1800588 | T | C | 0.21461 | -0.029769 | 0.002366 | 2.65E-36 | 25OHD | 443734 |
| rs1800775 | A | C | 0.486317 | -0.016619 | 0.00195 | 1.56E-17 | 25OHD | 443734 |
| rs1858889 | C | A | 0.50084 | 0.012836 | 0.001942 | 3.85E-11 | 25OHD | 443734 |
| rs188480917 | G | C | 0.010804 | -0.343291 | 0.009688 | 5E-275 | 25OHD | 443734 |
| rs1972994 | T | A | 0.64702 | -0.017509 | 0.002036 | 7.99E-18 | 25OHD | 443734 |
| rs2011425 | G | T | 0.07939 | -0.046356 | 0.00361 | 9.66E-38 | 25OHD | 443734 |
| rs2037511 | A | G | 0.165391 | 0.016025 | 0.002618 | 9.29E-10 | 25OHD | 443734 |
| rs2074735 | C | G | 0.064197 | 0.027256 | 0.003969 | 6.55E-12 | 25OHD | 443734 |
| rs2229742 | C | G | 0.103928 | -0.025738 | 0.00319 | 7.13E-16 | 25OHD | 443734 |
| rs2585442 | G | C | 0.246306 | 0.033609 | 0.002287 | 6.87E-49 | 25OHD | 443734 |
| rs261291 | C | T | 0.35583 | -0.022414 | 0.002033 | 2.89E-28 | 25OHD | 443734 |
| rs2762942 | A | G | 0.942083 | 0.053192 | 0.004321 | 7.99E-35 | 25OHD | 443734 |
| rs28364331 | G | A | 0.018845 | 0.061386 | 0.007186 | 1.31E-17 | 25OHD | 443734 |
| rs2847500 | A | G | 0.124391 | -0.021129 | 0.002949 | 7.79E-13 | 25OHD | 443734 |
| rs2909218 | T | C | 0.792744 | 0.016894 | 0.002418 | 2.81E-12 | 25OHD | 443734 |
| rs34726834 | T | C | 0.253615 | 0.013824 | 0.002239 | 6.65E-10 | 25OHD | 443734 |
| rs3750296 | C | G | 0.341423 | -0.020818 | 0.002042 | 2.09E-24 | 25OHD | 443734 |
| rs3768013 | A | G | 0.369605 | -0.01488 | 0.002011 | 1.37E-13 | 25OHD | 443734 |
| rs523583 | C | A | 0.469219 | 0.012174 | 0.001963 | 5.58E-10 | 25OHD | 443734 |
| rs532436 | A | G | 0.184388 | -0.015051 | 0.002515 | 2.17E-09 | 25OHD | 443734 |
| rs536006581 | G | G | 0.008644 | -0.17381 | 0.014129 | 8.87E-35 | 25OHD | 443734 |
| rs56044892 | T | C | 0.2107 | 0.015388 | 0.00244 | 2.85E-10 | 25OHD | 443734 |
| rs57631352 | G | A | 0.297255 | -0.012866 | 0.002128 | 1.48E-09 | 25OHD | 443734 |
| rs577185477 | C | T | 0.01469 | -0.379366 | 0.009583 | 1.624E-342 | 25OHD | 443734 |
| rs58073039 | G | A | 0.298368 | -0.014119 | 0.002109 | 2.16E-11 | 25OHD | 443734 |
| rs58542926 | T | C | 0.075847 | 0.032488 | 0.00367 | 8.57E-19 | 25OHD | 443734 |
| rs6123359 | G | A | 0.105192 | 0.032345 | 0.003213 | 7.74E-24 | 25OHD | 443734 |
| rs6127099 | T | A | 0.279034 | -0.036797 | 0.002219 | 9.3E-62 | 25OHD | 443734 |
| rs62007299 | A | G | 0.709377 | -0.014437 | 0.002145 | 1.69E-11 | 25OHD | 443734 |
| rs6438900 | G | C | 0.260706 | 0.013584 | 0.002221 | 9.59E-10 | 25OHD | 443734 |
| rs6698680 | G | A | 0.464195 | -0.011928 | 0.001947 | 8.99E-10 | 25OHD | 443734 |
| rs6724965 | G | A | 0.171608 | -0.016541 | 0.002573 | 1.29E-10 | 25OHD | 443734 |
| rs6773343 | T | C | 0.720232 | 0.01268 | 0.002171 | 5.2E-09 | 25OHD | 443734 |
| rs705117 | T | C | 0.849453 | -0.034494 | 0.002734 | 1.71E-36 | 25OHD | 443734 |
| rs73015021 | G | A | 0.121031 | 0.023034 | 0.002983 | 1.15E-14 | 25OHD | 443734 |
| rs7519574 | A | G | 0.181694 | 0.016991 | 0.002536 | 2.09E-11 | 25OHD | 443734 |
| rs7528419 | G | A | 0.224958 | 0.019031 | 0.002321 | 2.41E-16 | 25OHD | 443734 |
| rs7569755 | A | G | 0.292374 | 0.013923 | 0.002142 | 8.03E-11 | 25OHD | 443734 |
| rs7718395 | G | C | 0.319522 | 0.012632 | 0.002096 | 1.67E-09 | 25OHD | 443734 |
| rs77924615 | A | G | 0.197773 | -0.015792 | 0.002464 | 1.46E-10 | 25OHD | 443734 |
| rs7828742 | G | A | 0.596853 | -0.02193 | 0.00199 | 3.06E-28 | 25OHD | 443734 |
| rs78649910 | A | T | 0.110004 | -0.018331 | 0.003122 | 4.32E-09 | 25OHD | 443734 |
| rs8018720 | C | G | 0.820235 | -0.031949 | 0.002546 | 4.04E-36 | 25OHD | 443734 |
| rs804280 | A | C | 0.581959 | 0.013033 | 0.001978 | 4.43E-11 | 25OHD | 443734 |
| rs8063706 | T | A | 0.272828 | 0.012968 | 0.002198 | 3.64E-09 | 25OHD | 443734 |
| rs8091117 | A | C | 0.065396 | -0.024071 | 0.003943 | 1.03E-09 | 25OHD | 443734 |
| rs8103262 | C | T | 0.305004 | 0.012519 | 0.002114 | 3.18E-09 | 25OHD | 443734 |
| rs867772 | G | A | 0.681808 | -0.013838 | 0.002091 | 3.64E-11 | 25OHD | 443734 |
| rs960596 | T | C | 0.339588 | 0.012414 | 0.002076 | 2.23E-09 | 25OHD | 443734 |
| rs964184 | C | G | 0.863547 | 0.03977 | 0.002858 | 5.11E-44 | 25OHD | 443734 |
| rs2934744 | A | C | 0.643546 | -0.022405 | 0.002119 | 3.96E-26 | 25OHD | 443734 |
| rs115045402 | A | G | 0.026334 | 0.10713 | 0.006843 | 3.05E-55 | 25OHD | 443734 |
| rs201561609 | T | C | 0.986941 | -0.129276 | 0.011811 | 6.99E-28 | 25OHD | 443734 |
| rs185433896 | A | C | 0.993156 | -0.245785 | 0.018929 | 1.5E-38 | 25OHD | 443734 |
| rs189918701 | G | A | 0.997322 | -0.237546 | 0.02898 | 2.47E-16 | 25OHD | 443734 |
| rs375984409 | G | A | 0.991891 | -0.228513 | 0.017679 | 3.22E-38 | 25OHD | 443734 |
| rs144613541 | G | A | 0.290985 | 0.015413 | 0.002244 | 6.49E-12 | 25OHD | 443734 |
| rs150597413 | T | G | 0.004002 | 0.104852 | 0.016034 | 6.18E-11 | 25OHD | 443734 |
| rs138726443 | A | G | 0.00482 | 0.11212 | 0.014457 | 8.81E-15 | 25OHD | 443734 |
| rs61816761 | A | G | 0.023096 | 0.125479 | 0.006905 | 8.57E-74 | 25OHD | 443734 |
| rs576242124 | A | G | 0.011317 | 0.114432 | 0.014508 | 3.08E-15 | 25OHD | 443734 |
| rs184958517 | T | A | 0.993064 | -0.134888 | 0.017263 | 5.55E-15 | 25OHD | 443734 |
| rs558560635 | G | A | 0.99774 | -0.271577 | 0.033558 | 5.83E-16 | 25OHD | 443734 |
| rs11127048 | A | G | 0.616569 | 0.018107 | 0.002038 | 6.41E-19 | 25OHD | 443734 |
| rs7650253 | A | T | 0.689684 | 0.014562 | 0.002282 | 1.76E-10 | 25OHD | 443734 |
| rs7699711 | T | G | 0.454848 | -0.02864 | 0.001949 | 6.97E-49 | 25OHD | 443734 |
| rs529640451 | C | G | 0.997488 | 0.232802 | 0.027452 | 2.25E-17 | 25OHD | 443734 |
| rs528776789 | A | G | 0.993104 | 0.177644 | 0.015301 | 3.67E-31 | 25OHD | 443734 |
| rs113938679 | A | G | 0.005873 | -0.183841 | 0.014685 | 5.88E-36 | 25OHD | 443734 |
| rs564377207 | G | C | 0.995541 | -0.199047 | 0.020796 | 1.05E-21 | 25OHD | 443734 |
| rs186897112 | G | A | 0.998019 | 0.247082 | 0.03402 | 3.79E-13 | 25OHD | 443734 |
| rs557657187 | G | A | 0.99894 | 0.365446 | 0.045197 | 6.18E-16 | 25OHD | 443734 |
| rs145432346 | C | T | 0.825873 | 0.108617 | 0.003006 | 6.78E-286 | 25OHD | 443734 |
| rs560384646 | C | A | 0.023835 | -0.193328 | 0.008601 | 6.91E-112 | 25OHD | 443734 |
| rs200641845 | T | A | 0.545188 | 0.017645 | 0.002356 | 6.92E-14 | 25OHD | 443734 |
| rs565277381 | T | G | 0.998975 | 0.308457 | 0.047244 | 6.62E-11 | 25OHD | 443734 |
| rs3775150 | C | T | 0.262221 | -0.090838 | 0.002474 | 3.9E-295 | 25OHD | 443734 |
| rs222026 | T | A | 0.870776 | -0.052159 | 0.002996 | 6.98E-68 | 25OHD | 443734 |
| rs190688847 | C | T | 0.997836 | 0.290557 | 0.032893 | 1.02E-18 | 25OHD | 443734 |
| rs184291421 | C | T | 0.994155 | 0.170103 | 0.015324 | 1.25E-28 | 25OHD | 443734 |
| rs188838036 | A | G | 0.995469 | 0.17944 | 0.017666 | 3.07E-24 | 25OHD | 443734 |
| rs186881826 | A | T | 0.223069 | 0.045907 | 0.002469 | 3.64E-77 | 25OHD | 443734 |
| rs186441690 | G | A | 0.997189 | -0.26711 | 0.030494 | 1.96E-18 | 25OHD | 443734 |
| rs546541682 | T | G | 0.994255 | -0.156566 | 0.017885 | 2.06E-18 | 25OHD | 443734 |
| rs143106299 | T | A | 0.005747 | -0.169038 | 0.015251 | 1.5E-28 | 25OHD | 443734 |
| rs192785674 | A | G | 0.996588 | 0.168744 | 0.025969 | 8.14E-11 | 25OHD | 443734 |
| rs3822868 | G | A | 0.835029 | 0.021918 | 0.002745 | 1.41E-15 | 25OHD | 443734 |
| rs538325438 | C | A | 0.998583 | 0.227119 | 0.031549 | 6.07E-13 | 25OHD | 443734 |
| rs373514022 | C | T | 0.998234 | 0.202756 | 0.029333 | 4.77E-12 | 25OHD | 443734 |
| rs571618690 | A | C | 0.998605 | 0.366496 | 0.031416 | 1.9E-31 | 25OHD | 443734 |
| rs191379475 | G | A | 0.98893 | -0.102767 | 0.012908 | 1.7E-15 | 25OHD | 443734 |
| rs561089663 | G | C | 0.998499 | 0.409455 | 0.029769 | 4.79E-43 | 25OHD | 443734 |
| rs10832218 | C | T | 0.19774 | -0.034215 | 0.002912 | 7.09E-32 | 25OHD | 443734 |
| rs117206369 | T | C | 0.998354 | 0.467583 | 0.031883 | 1.07E-48 | 25OHD | 443734 |
| rs567876843 | G | T | 0.995283 | 0.541999 | 0.018921 | 1.83E-180 | 25OHD | 443734 |
| rs148514005 | T | C | 0.005501 | -0.446821 | 0.015421 | 1.37E-184 | 25OHD | 443734 |
| rs571484036 | A | G | 0.998325 | -0.217176 | 0.026698 | 4.13E-16 | 25OHD | 443734 |
| rs554808052 | C | A | 0.998315 | 0.348989 | 0.026366 | 5.41E-40 | 25OHD | 443734 |
| rs187443664 | T | G | 0.98867 | -0.106498 | 0.013059 | 3.49E-16 | 25OHD | 443734 |
| rs534042887 | G | T | 0.996899 | 0.391563 | 0.020379 | 2.82E-82 | 25OHD | 443734 |
| rs532836473 | G | A | 0.998425 | 0.435785 | 0.03131 | 4.9E-44 | 25OHD | 443734 |
| rs201501563 | T | C | 0.122197 | -0.066474 | 0.003851 | 9.17E-67 | 25OHD | 443734 |
| rs117576073 | T | G | 0.012385 | -0.114573 | 0.008813 | 1.22E-38 | 25OHD | 443734 |
| rs150585703 | G | C | 0.996054 | 0.481848 | 0.020273 | 7.16E-125 | 25OHD | 443734 |
| rs574992951 | C | T | 0.991488 | 0.086717 | 0.014741 | 4.04E-09 | 25OHD | 443734 |
| rs567415847 | G | A | 0.997973 | 0.283381 | 0.036634 | 1.03E-14 | 25OHD | 443734 |
| rs574615332 | A | C | 0.99727 | -0.286655 | 0.025845 | 1.38E-28 | 25OHD | 443734 |
| rs549940584 | T | C | 0.013335 | 0.182748 | 0.010158 | 2.31E-72 | 25OHD | 443734 |
| rs200454003 | T | C | 0.264587 | -0.0867 | 0.002536 | 3.68E-256 | 25OHD | 443734 |
| rs10793129 | A | G | 0.090078 | 0.024492 | 0.003468 | 1.64E-12 | 25OHD | 443734 |
| rs9668081 | T | C | 0.47058 | 0.0116 | 0.001988 | 5.38E-09 | 25OHD | 443734 |
| rs61937878 | T | C | 0.006418 | 0.119493 | 0.012369 | 4.43E-22 | 25OHD | 443734 |
| rs71383766 | T | C | 0.419574 | 0.012569 | 0.002065 | 1.15E-09 | 25OHD | 443734 |
| rs3814995 | T | C | 0.31224 | -0.014733 | 0.002109 | 2.83E-12 | 25OHD | 443734 |
| rs1065853 | T | G | 0.082083 | 0.027397 | 0.00367 | 8.32E-14 | 25OHD | 443734 |
| rs112285002 | T | C | 0.159529 | 0.060321 | 0.002701 | 1.77E-110 | 25OHD | 443734 |
| rs62130059 | C | A | 0.335823 | -0.02731 | 0.002255 | 9.25E-34 | 25OHD | 443734 |
| rs2232315 | A | G | 0.03 | 0.74 | 0.15 | 0.00000126 | lycopene | 441 |
| rs341075 | A | G | 0.02 | -0.87 | 0.17 | 0.000000575 | lycopene | 441 |
| rs4635297 | A | C | 0.08 | 0.26 | 0.05 | 0.000000646 | lycopene | 441 |
| rs6108801 | C | T | 0.04 | -0.48 | 0.09 | 0.000000407 | lycopene | 441 |
| rs7680948 | A | C | 0.2 | -0.19 | 0.03 | 4.97E-09 | lycopene | 441 |
| rs10882272 | C | T | 0.35 | -0.03 | 0.004 | 6.51E-15 | retinol | 5006 |
| rs1667255 | C | A | 0.31 | 0.03 | 0.004 | 6.35E-14 | retinol | 5006 |
| rs11057830 | A | G | 0.15 | 0.03 | 0.01 | 8.2E-09 | alpha-tocopherol | 7781 |
| rs2108622 | T | C | 0.21 | 0.03 | 0.01 | 1.4E-10 | alpha-tocopherol | 7781 |
| rs964184 | G | C | 0.15 | 0.04 | 0.01 | 7.8E-12 | alpha-tocopherol | 7781 |
| rs11645428 | A | G | 0.345 | -0.129 | 0.015 | 1.5E-17 | beta-carotene | 3918 |
| rs6420424 | A | G | 0.39 | 0.155 | 0.022 | 6.5E-13 | beta-carotene | 1793 |
| rs6564851 | G | T | 0.395 | 0.149 | 0.015 | 1.6E-24 | beta-carotene | 3881 |
| rs8044334 | G | T | 0.318 | 0.109 | 0.015 | 9.3E-13 | beta-carotene | 3915 |
| rs10129874 | C | T | 0.17 | 0.025 | 0.004 | 3.95E-13 | Calcium | 313903 |
| rs10224210 | C | T | 0.72 | 0.019 | 0.003 | 6.86827E-11 | Calcium | 313903 |
| rs1035798 | A | G | 0.2 | 0.019 | 0.003 | 5.11974E-09 | Calcium | 313903 |
| rs1036332 | C | A | 0.26 | 0.022 | 0.003 | 2.09049E-13 | Calcium | 313903 |
| rs10444863 | T | C | 0.21 | 0.02 | 0.003 | 1.02192E-09 | Calcium | 313903 |
| rs1048603 | A | G | 0.31 | 0.02 | 0.003 | 1.04867E-12 | Calcium | 313903 |
| rs1064608 | G | C | 0.65 | 0.021 | 0.003 | 1.34853E-14 | Calcium | 313903 |
| rs10739679 | G | A | 0.36 | 0.034 | 0.003 | 9.18806E-35 | Calcium | 313903 |
| rs10754439 | T | G | 0.58 | 0.015 | 0.003 | 3.7754E-08 | Calcium | 313903 |
| rs10898822 | A | G | 0.51 | 0.015 | 0.003 | 1.91385E-08 | Calcium | 313903 |
| rs10917386 | T | C | 0.31 | 0.019 | 0.003 | 8.49055E-12 | Calcium | 313903 |
| rs11078597 | C | T | 0.81 | 0.052 | 0.003 | 9.55207E-54 | Calcium | 313903 |
| rs11117777 | C | T | 0.16 | 0.022 | 0.004 | 1.62239E-09 | Calcium | 313903 |
| rs11122848 | A | G | 0.47 | 0.015 | 0.003 | 1.67316E-08 | Calcium | 313903 |
| rs11187838 | G | A | 0.43 | 0.019 | 0.003 | 1.36595E-12 | Calcium | 313903 |
| rs11218725 | G | A | 0.62 | 0.015 | 0.003 | 2.03277E-08 | Calcium | 313903 |
| rs112371897 | T | C | 0.91 | 0.071 | 0.004 | 2.53863E-56 | Calcium | 313903 |
| rs113911787 | A | G | 0.77 | 0.017 | 0.003 | 4.12564E-08 | Calcium | 313903 |
| rs1150781 | C | G | 0.91 | 0.044 | 0.005 | 1.91683E-21 | Calcium | 313903 |
| rs11538349 | C | T | 0.1 | 0.03 | 0.004 | 1.53435E-11 | Calcium | 313903 |
| rs11577605 | A | G | 0.12 | 0.034 | 0.004 | 2.9841E-17 | Calcium | 313903 |
| rs115946508 | A | C | 0.89 | 0.036 | 0.004 | 8.95189E-18 | Calcium | 313903 |
| rs116004654 | T | C | 0.94 | 0.059 | 0.006 | 9.09585E-25 | Calcium | 313903 |
| rs116140498 | C | T | 0.95 | 0.05 | 0.006 | 2.4985E-17 | Calcium | 313903 |
| rs11629876 | C | T | 0.33 | 0.018 | 0.003 | 2.92981E-11 | Calcium | 313903 |
| rs11632520 | C | T | 0.17 | 0.029 | 0.003 | 1.0087E-16 | Calcium | 313903 |
| rs11687510 | C | T | 0.36 | 0.018 | 0.003 | 1.20694E-10 | Calcium | 313903 |
| rs11730491 | T | G | 0.83 | 0.022 | 0.004 | 3.20471E-10 | Calcium | 313903 |
| rs11746728 | C | T | 0.65 | 0.016 | 0.003 | 4.73644E-09 | Calcium | 313903 |
| rs11753096 | C | T | 0.46 | 0.019 | 0.003 | 6.89026E-13 | Calcium | 313903 |
| rs1177274 | T | G | 0.56 | 0.028 | 0.003 | 1.45487E-26 | Calcium | 313903 |
| rs11777067 | T | C | 0.77 | 0.02 | 0.003 | 3.90393E-11 | Calcium | 313903 |
| rs11792928 | C | T | 0.29 | 0.017 | 0.003 | 5.27478E-09 | Calcium | 313903 |
| rs12212449 | C | T | 0.61 | 0.031 | 0.003 | 9.55398E-31 | Calcium | 313903 |
| rs12294466 | T | C | 0.92 | 0.029 | 0.005 | 4.97125E-09 | Calcium | 313903 |
| rs12339541 | C | A | 0.06 | 0.056 | 0.005 | 1.21993E-25 | Calcium | 313903 |
| rs12378991 | G | A | 0.08 | 0.036 | 0.005 | 1.08331E-13 | Calcium | 313903 |
| rs12519940 | C | T | 0.28 | 0.027 | 0.003 | 2.75326E-20 | Calcium | 313903 |
| rs12534476 | G | A | 0.17 | 0.021 | 0.003 | 1.07409E-09 | Calcium | 313903 |
| rs12583851 | T | C | 0.75 | 0.022 | 0.003 | 1.48338E-13 | Calcium | 313903 |
| rs1260326 | T | C | 0.61 | 0.047 | 0.003 | 3.46271E-69 | Calcium | 313903 |
| rs12613807 | C | T | 0.56 | 0.018 | 0.003 | 9.55246E-12 | Calcium | 313903 |
| rs1262217 | A | G | 0.17 | 0.02 | 0.003 | 4.99322E-09 | Calcium | 313903 |
| rs12675477 | T | C | 0.73 | 0.017 | 0.003 | 1.60924E-08 | Calcium | 313903 |
| rs12794834 | C | T | 0.52 | 0.015 | 0.003 | 3.08281E-08 | Calcium | 313903 |
| rs12917235 | C | T | 0.37 | 0.027 | 0.003 | 2.32823E-23 | Calcium | 313903 |
| rs12918968 | A | C | 0.44 | 0.032 | 0.003 | 1.06328E-34 | Calcium | 313903 |
| rs12922549 | C | T | 0.24 | 0.023 | 0.003 | 2.42134E-13 | Calcium | 313903 |
| rs12932755 | A | G | 0.51 | 0.017 | 0.003 | 1.61858E-10 | Calcium | 313903 |
| rs12933677 | T | C | 0.47 | 0.015 | 0.003 | 6.97112E-09 | Calcium | 313903 |
| rs12974855 | A | G | 0.86 | 0.026 | 0.004 | 1.96993E-11 | Calcium | 313903 |
| rs12998379 | G | A | 0.19 | 0.026 | 0.003 | 1.42987E-14 | Calcium | 313903 |
| rs1303 | T | G | 0.74 | 0.021 | 0.003 | 4.69422E-12 | Calcium | 313903 |
| rs13107325 | C | T | 0.93 | 0.059 | 0.005 | 1.14393E-32 | Calcium | 313903 |
| rs13108218 | A | G | 0.62 | 0.039 | 0.003 | 4.07193E-47 | Calcium | 313903 |
| rs13254847 | C | T | 0.22 | 0.023 | 0.003 | 1.33037E-12 | Calcium | 313903 |
| rs13325 | G | A | 0.17 | 0.025 | 0.004 | 1.44182E-12 | Calcium | 313903 |
| rs13389219 | C | T | 0.39 | 0.016 | 0.003 | 1.01948E-09 | Calcium | 313903 |
| rs1354034 | T | C | 0.6 | 0.019 | 0.003 | 3.11364E-13 | Calcium | 313903 |
| rs1476698 | A | G | 0.37 | 0.023 | 0.003 | 7.70461E-17 | Calcium | 313903 |
| rs1495747 | C | T | 0.28 | 0.019 | 0.003 | 2.36463E-10 | Calcium | 313903 |
| rs1604081 | C | T | 0.14 | 0.022 | 0.004 | 7.92618E-09 | Calcium | 313903 |
| rs1621676 | G | A | 0.25 | 0.017 | 0.003 | 3.96008E-08 | Calcium | 313903 |
| rs1657502 | T | G | 0.62 | 0.02 | 0.003 | 4.21213E-13 | Calcium | 313903 |
| rs16844401 | A | G | 0.07 | 0.051 | 0.005 | 1.31487E-21 | Calcium | 313903 |
| rs1688131 | T | C | 0.71 | 0.036 | 0.003 | 1.79069E-35 | Calcium | 313903 |
| rs17164683 | C | T | 0.27 | 0.02 | 0.003 | 1.65999E-11 | Calcium | 313903 |
| rs1749849 | C | T | 0.42 | 0.021 | 0.003 | 2.47267E-15 | Calcium | 313903 |
| rs17668044 | A | G | 0.71 | 0.018 | 0.003 | 4.65916E-10 | Calcium | 313903 |
| rs17774672 | G | A | 0.16 | 0.025 | 0.004 | 2.08696E-12 | Calcium | 313903 |
| rs1780316 | C | T | 0.06 | 0.037 | 0.006 | 1.36795E-11 | Calcium | 313903 |
| rs1801282 | C | G | 0.88 | 0.039 | 0.004 | 7.94628E-22 | Calcium | 313903 |
| rs1801725 | T | G | 0.13 | 0.191 | 0.004 | 0 | Calcium | 313903 |
| rs1827293 | G | A | 0.45 | 0.024 | 0.003 | 2.9726E-19 | Calcium | 313903 |
| rs1858800 | T | C | 0.65 | 0.032 | 0.003 | 1.60792E-30 | Calcium | 313903 |
| rs1875272 | G | A | 0.75 | 0.026 | 0.003 | 9.80579E-17 | Calcium | 313903 |
| rs2004315 | T | C | 0.38 | 0.033 | 0.003 | 5.84062E-35 | Calcium | 313903 |
| rs2070179 | C | T | 0.24 | 0.017 | 0.003 | 3.40475E-08 | Calcium | 313903 |
| rs2100431 | A | C | 0.3 | 0.017 | 0.003 | 3.03819E-09 | Calcium | 313903 |
| rs218671 | G | T | 0.55 | 0.016 | 0.003 | 6.43843E-10 | Calcium | 313903 |
| rs2245715 | A | G | 0.1 | 0.024 | 0.004 | 4.44677E-08 | Calcium | 313903 |
| rs2288920 | G | T | 0.83 | 0.026 | 0.004 | 1.12267E-13 | Calcium | 313903 |
| rs2298615 | C | T | 0.23 | 0.019 | 0.003 | 1.26733E-09 | Calcium | 313903 |
| rs2303914 | G | T | 0.52 | 0.02 | 0.003 | 2.07232E-13 | Calcium | 313903 |
| rs2343592 | A | G | 0.27 | 0.023 | 0.003 | 2.70504E-15 | Calcium | 313903 |
| rs2419886 | C | T | 0.26 | 0.023 | 0.003 | 2.90869E-14 | Calcium | 313903 |
| rs2448036 | G | T | 0.11 | 0.029 | 0.004 | 4.28142E-11 | Calcium | 313903 |
| rs2520265 | A | G | 0.29 | 0.022 | 0.003 | 5.22765E-14 | Calcium | 313903 |
| rs2546056 | T | C | 0.86 | 0.026 | 0.004 | 8.33412E-12 | Calcium | 313903 |
| rs2585135 | A | G | 0.54 | 0.015 | 0.003 | 1.85298E-08 | Calcium | 313903 |
| rs2636695 | G | T | 0.77 | 0.021 | 0.003 | 2.74084E-11 | Calcium | 313903 |
| rs2681417 | G | A | 0.93 | 0.037 | 0.005 | 2.81984E-13 | Calcium | 313903 |
| rs2785171 | G | A | 0.61 | 0.015 | 0.003 | 1.75922E-08 | Calcium | 313903 |
| rs2807880 | C | T | 0.18 | 0.02 | 0.003 | 1.86997E-09 | Calcium | 313903 |
| rs2855799 | G | A | 0.81 | 0.018 | 0.003 | 4.06011E-08 | Calcium | 313903 |
| rs28616221 | A | G | 0.82 | 0.046 | 0.003 | 4.52572E-42 | Calcium | 313903 |
| rs2918247 | A | G | 0.23 | 0.017 | 0.003 | 2.48065E-08 | Calcium | 313903 |
| rs3026445 | T | C | 0.37 | 0.018 | 0.003 | 2.51649E-11 | Calcium | 313903 |
| rs302655 | G | T | 0.43 | 0.02 | 0.003 | 3.54605E-14 | Calcium | 313903 |
| rs308981 | C | T | 0.12 | 0.023 | 0.004 | 2.25167E-08 | Calcium | 313903 |
| rs3130618 | C | A | 0.8 | 0.021 | 0.003 | 2.50148E-10 | Calcium | 313903 |
| rs34010237 | A | G | 0.84 | 0.03 | 0.004 | 1.3939E-17 | Calcium | 313903 |
| rs34372369 | A | G | 0.05 | 0.044 | 0.006 | 1.27405E-13 | Calcium | 313903 |
| rs34667500 | A | G | 0.88 | 0.038 | 0.004 | 3.13949E-21 | Calcium | 313903 |
| rs34895054 | C | G | 0.26 | 0.019 | 0.003 | 4.86149E-10 | Calcium | 313903 |
| rs35320690 | C | T | 0.72 | 0.026 | 0.003 | 4.71196E-19 | Calcium | 313903 |
| rs35587941 | T | G | 0.57 | 0.015 | 0.003 | 1.36714E-08 | Calcium | 313903 |
| rs35674179 | C | A | 0.9 | 0.026 | 0.004 | 2.00374E-09 | Calcium | 313903 |
| rs35758545 | C | T | 0.35 | 0.016 | 0.003 | 3.10367E-09 | Calcium | 313903 |
| rs35852840 | A | C | 0.94 | 0.031 | 0.006 | 4.69698E-08 | Calcium | 313903 |
| rs36086195 | T | C | 0.42 | 0.018 | 0.003 | 3.7801E-12 | Calcium | 313903 |
| rs36104352 | C | A | 0.88 | 0.03 | 0.004 | 7.60269E-14 | Calcium | 313903 |
| rs3740690 | T | C | 0.44 | 0.015 | 0.003 | 1.92365E-08 | Calcium | 313903 |
| rs3741628 | G | T | 0.57 | 0.022 | 0.003 | 1.23769E-16 | Calcium | 313903 |
| rs3794695 | T | C | 0.81 | 0.019 | 0.003 | 1.21301E-08 | Calcium | 313903 |
| rs3798236 | T | C | 0.37 | 0.017 | 0.003 | 7.9284E-10 | Calcium | 313903 |
| rs3931841 | A | G | 0.68 | 0.027 | 0.003 | 2.36658E-21 | Calcium | 313903 |
| rs41264630 | A | G | 0.92 | 0.027 | 0.005 | 1.51368E-08 | Calcium | 313903 |
| rs413948 | G | A | 0.87 | 0.022 | 0.004 | 2.5898E-08 | Calcium | 313903 |
| rs4376797 | A | G | 0.65 | 0.026 | 0.003 | 5.74706E-21 | Calcium | 313903 |
| rs4594967 | A | G | 0.67 | 0.023 | 0.003 | 7.82553E-17 | Calcium | 313903 |
| rs4633480 | A | G | 0.44 | 0.021 | 0.003 | 2.43164E-15 | Calcium | 313903 |
| rs4758621 | A | G | 0.31 | 0.028 | 0.003 | 2.55479E-22 | Calcium | 313903 |
| rs4790310 | C | T | 0.57 | 0.022 | 0.003 | 3.31041E-17 | Calcium | 313903 |
| rs4805129 | T | C | 0.63 | 0.02 | 0.003 | 1.72969E-13 | Calcium | 313903 |
| rs4915444 | C | A | 0.4 | 0.017 | 0.003 | 2.523E-10 | Calcium | 313903 |
| rs4917 | C | T | 0.36 | 0.038 | 0.003 | 5.42972E-43 | Calcium | 313903 |
| rs4925104 | G | T | 0.47 | 0.018 | 0.003 | 1.90224E-11 | Calcium | 313903 |
| rs4976647 | C | A | 0.67 | 0.018 | 0.003 | 1.02783E-10 | Calcium | 313903 |
| rs498490 | C | T | 0.16 | 0.033 | 0.004 | 8.71367E-21 | Calcium | 313903 |
| rs507666 | G | A | 0.19 | 0.021 | 0.003 | 2.69319E-10 | Calcium | 313903 |
| rs55754498 | C | T | 0.05 | 0.033 | 0.006 | 2.36508E-08 | Calcium | 313903 |
| rs56230940 | T | C | 0.93 | 0.031 | 0.005 | 3.1995E-10 | Calcium | 313903 |
| rs56252617 | T | C | 0.6 | 0.015 | 0.003 | 4.02342E-08 | Calcium | 313903 |
| rs56313825 | A | G | 0.6 | 0.015 | 0.003 | 1.4418E-08 | Calcium | 313903 |
| rs56397046 | C | T | 0.33 | 0.021 | 0.003 | 2.24467E-14 | Calcium | 313903 |
| rs5751350 | A | G | 0.67 | 0.016 | 0.003 | 1.75396E-08 | Calcium | 313903 |
| rs57564578 | G | A | 0.07 | 0.033 | 0.005 | 1.07841E-10 | Calcium | 313903 |
| rs58673639 | G | T | 0.79 | 0.018 | 0.003 | 6.34204E-09 | Calcium | 313903 |
| rs6013892 | A | C | 0.93 | 0.032 | 0.005 | 6.18562E-10 | Calcium | 313903 |
| rs60155540 | G | A | 0.2 | 0.021 | 0.003 | 2.57318E-10 | Calcium | 313903 |
| rs611150 | T | C | 0.82 | 0.022 | 0.003 | 9.17898E-11 | Calcium | 313903 |
| rs6118 | C | T | 0.9 | 0.03 | 0.004 | 8.8638E-12 | Calcium | 313903 |
| rs61594679 | C | T | 0.29 | 0.018 | 0.003 | 4.22795E-10 | Calcium | 313903 |
| rs61649210 | G | A | 0.45 | 0.015 | 0.003 | 1.34743E-08 | Calcium | 313903 |
| rs61770531 | C | T | 0.91 | 0.036 | 0.005 | 8.12615E-15 | Calcium | 313903 |
| rs61779309 | T | C | 0.21 | 0.02 | 0.003 | 9.78708E-10 | Calcium | 313903 |
| rs62134669 | C | T | 0.85 | 0.024 | 0.004 | 1.21725E-10 | Calcium | 313903 |
| rs62292542 | G | A | 0.11 | 0.032 | 0.004 | 2.37303E-14 | Calcium | 313903 |
| rs62362239 | C | T | 0.67 | 0.02 | 0.003 | 7.53469E-13 | Calcium | 313903 |
| rs62439474 | T | C | 0.7 | 0.022 | 0.003 | 4.81157E-14 | Calcium | 313903 |
| rs6560613 | C | T | 0.91 | 0.034 | 0.005 | 6.78512E-13 | Calcium | 313903 |
| rs6580981 | G | A | 0.46 | 0.019 | 0.003 | 4.68516E-13 | Calcium | 313903 |
| rs6590227 | C | T | 0.12 | 0.025 | 0.004 | 5.41614E-10 | Calcium | 313903 |
| rs66527777 | C | T | 0.17 | 0.022 | 0.004 | 2.79147E-10 | Calcium | 313903 |
| rs6680117 | C | T | 0.18 | 0.027 | 0.003 | 3.19799E-15 | Calcium | 313903 |
| rs6719061 | C | T | 0.6 | 0.019 | 0.003 | 6.18701E-13 | Calcium | 313903 |
| rs6722613 | G | A | 0.57 | 0.016 | 0.003 | 5.31389E-10 | Calcium | 313903 |
| rs6731551 | T | C | 0.48 | 0.015 | 0.003 | 1.25899E-08 | Calcium | 313903 |
| rs6734610 | G | A | 0.48 | 0.023 | 0.003 | 1.58129E-18 | Calcium | 313903 |
| rs6741561 | C | T | 0.39 | 0.04 | 0.003 | 1.84951E-50 | Calcium | 313903 |
| rs681664 | C | T | 0.22 | 0.018 | 0.003 | 1.26984E-08 | Calcium | 313903 |
| rs6830950 | T | C | 0.27 | 0.017 | 0.003 | 1.50081E-08 | Calcium | 313903 |
| rs6841429 | C | A | 0.17 | 0.041 | 0.004 | 1.02224E-31 | Calcium | 313903 |
| rs6894167 | C | T | 0.52 | 0.017 | 0.003 | 5.07118E-11 | Calcium | 313903 |
| rs6909201 | A | G | 0.48 | 0.043 | 0.003 | 4.11896E-60 | Calcium | 313903 |
| rs697852 | G | A | 0.82 | 0.022 | 0.003 | 3.13159E-10 | Calcium | 313903 |
| rs7012637 | A | G | 0.52 | 0.023 | 0.003 | 5.47617E-18 | Calcium | 313903 |
| rs7208714 | G | A | 0.75 | 0.027 | 0.003 | 1.74727E-19 | Calcium | 313903 |
| rs7221118 | T | C | 0.21 | 0.021 | 0.003 | 1.17994E-10 | Calcium | 313903 |
| rs722298 | A | G | 0.56 | 0.016 | 0.003 | 3.38215E-09 | Calcium | 313903 |
| rs72740967 | C | T | 0.31 | 0.02 | 0.003 | 1.14853E-12 | Calcium | 313903 |
| rs72847071 | G | A | 0.09 | 0.03 | 0.005 | 7.17409E-11 | Calcium | 313903 |
| rs72999033 | T | C | 0.93 | 0.043 | 0.005 | 5.1182E-16 | Calcium | 313903 |
| rs7313874 | T | C | 0.6 | 0.017 | 0.003 | 6.1343E-10 | Calcium | 313903 |
| rs73183155 | C | T | 0.23 | 0.028 | 0.003 | 1.39979E-18 | Calcium | 313903 |
| rs7320843 | C | T | 0.14 | 0.036 | 0.004 | 2.59573E-22 | Calcium | 313903 |
| rs7559013 | C | A | 0.87 | 0.026 | 0.004 | 2.06998E-11 | Calcium | 313903 |
| rs7568296 | T | C | 0.42 | 0.02 | 0.003 | 5.83183E-14 | Calcium | 313903 |
| rs760077 | A | T | 0.61 | 0.027 | 0.003 | 1.84319E-23 | Calcium | 313903 |
| rs76758508 | C | T | 0.32 | 0.015 | 0.003 | 4.19269E-08 | Calcium | 313903 |
| rs77722590 | A | G | 0.85 | 0.023 | 0.004 | 2.37764E-10 | Calcium | 313903 |
| rs7864156 | G | T | 0.61 | 0.018 | 0.003 | 1.42022E-11 | Calcium | 313903 |
| rs7913072 | G | A | 0.86 | 0.021 | 0.004 | 3.94832E-08 | Calcium | 313903 |
| rs7968405 | C | T | 0.82 | 0.022 | 0.003 | 2.23629E-10 | Calcium | 313903 |
| rs8011945 | G | T | 0.09 | 0.026 | 0.005 | 1.13843E-08 | Calcium | 313903 |
| rs8034835 | A | G | 0.47 | 0.019 | 0.003 | 9.95405E-13 | Calcium | 313903 |
| rs80350997 | A | G | 0.92 | 0.046 | 0.005 | 1.03396E-20 | Calcium | 313903 |
| rs8081353 | C | T | 0.1 | 0.025 | 0.004 | 1.21259E-08 | Calcium | 313903 |
| rs838718 | G | A | 0.48 | 0.047 | 0.003 | 9.09272E-73 | Calcium | 313903 |
| rs841572 | A | G | 0.59 | 0.033 | 0.003 | 1.30139E-35 | Calcium | 313903 |
| rs848492 | A | G | 0.28 | 0.027 | 0.003 | 1.00529E-19 | Calcium | 313903 |
| rs872629 | A | C | 0.89 | 0.02404 | 0.004 | 6.00422E-09 | Calcium | 313903 |
| rs900399 | G | A | 0.6 | 0.019 | 0.003 | 3.78794E-12 | Calcium | 313903 |
| rs915894 | T | G | 0.6 | 0.015 | 0.003 | 1.70483E-08 | Calcium | 313903 |
| rs926103 | C | T | 0.35 | 0.022 | 0.003 | 8.0186E-16 | Calcium | 313903 |
| rs9282641 | G | A | 0.92 | 0.036 | 0.005 | 2.71065E-14 | Calcium | 313903 |
| rs9388399 | T | C | 0.31 | 0.024 | 0.003 | 1.93927E-17 | Calcium | 313903 |
| rs9420589 | T | G | 0.56 | 0.017 | 0.003 | 6.73896E-11 | Calcium | 313903 |
| rs9530 | A | G | 0.55 | 0.032 | 0.003 | 3.63705E-34 | Calcium | 313903 |
| rs9562385 | A | C | 0.56 | 0.015 | 0.003 | 1.07785E-08 | Calcium | 313903 |
| rs9635741 | C | A | 0.93 | 0.039 | 0.005 | 8.13665E-15 | Calcium | 313903 |
| rs965344 | A | G | 0.79 | 0.031 | 0.003 | 1.07874E-21 | Calcium | 313903 |
| rs9806062 | G | T | 0.89 | 0.026 | 0.004 | 5.09945E-10 | Calcium | 313903 |
| rs11584885 | G | A | 0.28 | 0.026 | 0.003 | 9.85039E-19 | Calcium | 313903 |
| rs3217795 | A | G | 0.09 | 0.025 | 0.005 | 4.94574E-08 | Calcium | 313903 |
| rs4763297 | C | A | 0.57 | 0.015 | 0.003 | 5.79834E-09 | Calcium | 313903 |
| rs55633823 | C | T | 0.75 | 0.022 | 0.003 | 1.45084E-12 | Calcium | 313903 |
| rs7312673 | A | G | 0.07 | 0.043 | 0.005 | 2.41405E-17 | Calcium | 313903 |
| rs74753001 | C | T | 0.93 | 0.028 | 0.005 | 3.11559E-08 | Calcium | 313903 |
| rs80339483 | T | C | 0.93 | 0.031 | 0.005 | 1.81056E-09 | Calcium | 313903 |
| rs9895661 | C | T | 0.83 | 0.028 | 0.003 | 5.04342E-16 | Calcium | 313903 |
| rs1175550 | G | A | 0.22 | 0.198 | 0.032 | 5.03E-10 | Copper | 2603 |
| rs2769264 | G | T | 0.16 | 0.313 | 0.034 | 2.63E-20 | Copper | 2603 |
| rs1799945 | G | C | 0.15 | 0.189 | 0.01 | 1.1E-81 | Iron | 48972 |
| rs1800562 | A | G | 0.07 | 0.328 | 0.016 | 2.72E-97 | Iron | 48972 |
| rs855791 | G | A | 0.55 | 0.181 | 0.007 | 1.32E-139 | Iron | 48972 |
| rs11144134 | C | T | 0.08 | 0.011 | 0.001 | 8.21E-15 | Magnesium | 23829 |
| rs13146355 | A | G | 0.44 | 0.005 | 0.001 | 6.27E-13 | Magnesium | 23829 |
| rs3925584 | T | C | 0.55 | 0.006 | 0.001 | 5.2E-16 | Magnesium | 23829 |
| rs4072037 | T | C | 0.54 | 0.01 | 0.001 | 2.01E-36 | Magnesium | 23829 |
| rs448378 | A | G | 0.53 | 0.004 | 0.001 | 1.25E-08 | Magnesium | 23829 |
| rs7965584 | A | G | 0.71 | 0.007 | 0.001 | 1.05E-16 | Magnesium | 23829 |
| rs1697421 | A | G | 0.49 | 0.05 | 0.005 | 1.14E-27 | Phosphorus | 21726 |
| rs17265703 | A | G | 0.85 | 0.036 | 0.006 | 4.32E-09 | Phosphorus | 21733 |
| rs2970818 | A | T | 0.09 | 0.047 | 0.008 | 4.38E-09 | Phosphorus | 21734 |
| rs9469578 | C | T | 0.92 | 0.059 | 0.009 | 1.11E-11 | Phosphorus | 21734 |
| rs947583 | C | T | 0.29 | 0.035 | 0.005 | 3.45E-12 | Phosphorus | 21733 |
| rs1131603 | C | T | 0.06 | 0.19 | 0.017 | 4.3E-28 | Vitamin B12 | 37283 |
| rs1141321 | C | T | 0.63 | 0.061 | 0.007 | 1.4E-16 | Vitamin B12 | 37283 |
| rs1801222 | G | A | 0.59 | 0.11 | 0.007 | 1.1E-52 | Vitamin B12 | 37283 |
| rs2270655 | G | C | 0.94 | 0.066 | 0.016 | 0.000035 | Vitamin B12 | 37283 |
| rs34324219 | C | A | 0.88 | 0.21 | 0.011 | 8.8E-71 | Vitamin B12 | 37283 |
| rs3742801 | T | C | 0.29 | 0.045 | 0.008 | 0.000000053 | Vitamin B12 | 37283 |
| rs41281112 | C | T | 0.95 | 0.17 | 0.016 | 9.6E-27 | Vitamin B12 | 37283 |
| rs602662 | A | G | 0.6 | 0.16 | 0.008 | 4.1E-96 | Vitamin B12 | 37283 |
| rs778805 | A | G | 0.25 | 0.046 | 0.009 | 0.00000021 | Vitamin B12 | 37283 |
| rs1256335 | A | G | 0.79 | 0.14 | 0.02 | 1.4E-15 | Vitamin B6 | 4763 |
| rs4654748 | T | C | 0.52 | 0.1 | 0.01 | 4.3E-11 | Vitamin B6 | 4763 |
| rs10051765 | C | T | 0.342 | 0.039 | 0.007 | 3.64E-09 | vitamin C | 52018 |
| rs117885456 | A | G | 0.087 | 0.078 | 0.012 | 1.7E-11 | vitamin C | 52018 |
| rs13028225 | T | C | 0.857 | 0.102 | 0.009 | 2.38E-30 | vitamin C | 52018 |
| rs174547 | C | T | 0.328 | 0.036 | 0.007 | 3.84E-08 | vitamin C | 52018 |
| rs2559850 | A | G | 0.598 | 0.058 | 0.006 | 6.3E-20 | vitamin C | 52018 |
| rs33972313 | C | T | 0.968 | 0.36 | 0.018 | 4.61E-90 | vitamin C | 52018 |
| rs56738967 | C | G | 0.321 | 0.041 | 0.007 | 7.62E-10 | vitamin C | 52018 |
| rs6693447 | T | G | 0.551 | 0.039 | 0.006 | 6.25E-10 | vitamin C | 52018 |
| rs7740812 | G | A | 0.594 | 0.038 | 0.006 | 1.88E-09 | vitamin C | 52018 |
| rs10136000 | A | G | 0.283 | 0.04 | 0.007 | 1.33E-08 | vitamin C | 52018 |
| rs9895661 | T | C | 0.817 | 0.063 | 0.008 | 1.05E-14 | vitamin C | 52018 |
| rs1532423 | A | G | 0.37 | 0.178 | 0.026 | 6.4E-12 | Zinc | 2603 |
| rs2120019 | T | C | 0.79 | 0.287 | 0.033 | 1.55E-18 | Zinc | 2603 |
| rs4826508 | T | C | 0.48 | 0.21 | 0.03 | 1.4E-12 | Zinc | 2603 |
| rs174547 | C | T | 0.33 | 0.016 | 0.001 | 4E-64 | Alpha-linolenic acid (ALA) | 8631 |
| rs16966952 | G | A | 0.69 | 0.199 | 0.031 | 2.4E-10 | Arachidonic acid (AA) | 8631 |
| rs174547 | T | C | 0.68 | 1.691 | 0.025 | 3.3E-971 | Arachidonic acid (AA) | 8631 |
| rs16966952 | G | A | 0.69 | 0.2204 | 0.013 | 7.55E-65 | Dihomo-gamma-linolenic acid (DGLA) | 8631 |
| rs174547 | C | T | 0.33 | 0.355 | 0.0136 | 2.63E-151 | Dihomo-gamma-linolenic acid (DGLA) | 8631 |
| rs11604424 | T | C | 0.756672 | -0.08311 | 0.014241 | 7.84007E-09 | Docosahexaenoic acid (DHA) | 13495 |
| rs143988316 | T | C | 0.069487 | -0.150045 | 0.024351 | 1.09999E-09 | Docosahexaenoic acid (DHA) | 13494 |
| rs174546 | T | C | 0.402849 | -0.127635 | 0.012483 | 4.8095E-24 | Docosahexaenoic acid (DHA) | 13499 |
| rs2281591 | G | A | 0.13372 | -0.108394 | 0.018174 | 3.65999E-09 | Docosahexaenoic acid (DHA) | 13498 |
| rs261334 | C | G | 0.769129 | -0.110247 | 0.014749 | 1.44012E-13 | Docosahexaenoic acid (DHA) | 13498 |
| rs145717049 | T | C | 0.044058 | -0.201292 | 0.03275 | 1.21001E-09 | Docosahexaenoic acid (DHA) | 13491 |
| rs174547 | T | C | 0.67 | 0.075 | 0.003 | 4E-154 | Docosapentaenoic acid (DPA) | 8631 |
| rs3734398 | C | T | 0.43 | 0.04 | 0.003 | 1E-43 | Docosapentaenoic acid (DPA) | 8631 |
| rs780094 | T | C | 0.41 | 0.017 | 0.003 | 0.000000009 | Docosapentaenoic acid (DPA) | 8631 |
| rs174538 | G | A | 0.72 | 0.083 | 0.005 | 5E-58 | Eicosapentaenoic acid (EPA) | 8631 |
| rs3798713 | C | G | 0.43 | 0.035 | 0.005 | 2E-12 | Eicosapentaenoic acid (EPA) | 8631 |
| rs16966952 | G | A | 0.69 | 0.0061 | 0.0009 | 5.05E-11 | Gamma linolenic acid (GLA) | 8631 |
| rs174547 | T | C | 0.67 | 0.0156 | 0.0009 | 2.29E-72 | Gamma linolenic acid (GLA) | 8631 |
| rs1260326 | C | T | 0.638809 | -0.085678 | 0.009934 | 9.75E-18 | Isoleucine | 22549 |
| rs1440580 | A | T | 0.481233 | 0.072612 | 0.00941 | 1.68E-14 | Isoleucine | 24772 |
| rs12325419 | A | G | 0.120221 | -0.082062 | 0.014985 | 4.55E-08 | Leucine | 22500 |
| rs1260326 | C | T | 0.638971 | -0.079808 | 0.009937 | 1.07E-15 | Leucine | 22500 |
| rs17789027 | G | A | 0.384329 | 0.108594 | 0.009541 | 6.16E-30 | Leucine | 24725 |
| rs10402112 | A | T | 0.09963 | -0.171088 | 0.021087 | 7.89951E-16 | Linoleic acid (LA) | 13522 |
| rs12239737 | A | T | 0.260158 | -0.100493 | 0.014041 | 1.20005E-12 | Linoleic acid (LA) | 13523 |
| rs1260326 | C | T | 0.636686 | -0.082178 | 0.01269 | 1.28E-10 | Linoleic acid (LA) | 13527 |
| rs143341434 | T | C | 0.039532 | -0.17864 | 0.032309 | 4.03005E-08 | Linoleic acid (LA) | 13523 |
| rs144064722 | G | A | 0.026298 | 0.230203 | 0.039539 | 7.45006E-09 | Linoleic acid (LA) | 13520 |
| rs144723570 | T | C | 0.01117 | -0.332453 | 0.059815 | 3.42003E-08 | Linoleic acid (LA) | 13523 |
| rs17414716 | G | A | 0.028489 | -0.300064 | 0.037805 | 3.25987E-15 | Linoleic acid (LA) | 13522 |
| rs174418 | C | T | 0.562555 | -0.086064 | 0.012538 | 9.42106E-12 | Linoleic acid (LA) | 13524 |
| rs1800588 | T | C | 0.24922 | 0.129233 | 0.014469 | 7.40969E-19 | Linoleic acid (LA) | 13525 |
| rs4296389 | T | C | 0.325777 | -0.081438 | 0.013081 | 6.37001E-10 | Linoleic acid (LA) | 13521 |
| rs7412 | T | C | 0.057292 | -0.295042 | 0.028251 | 3.40017E-25 | Linoleic acid (LA) | 13523 |
| rs76366838 | A | G | 0.018475 | 0.287426 | 0.049651 | 9.06004E-09 | Linoleic acid (LA) | 13524 |
| rs769449 | A | G | 0.156683 | 0.142266 | 0.016956 | 8.04081E-17 | Linoleic acid (LA) | 13524 |
| rs79225634 | T | C | 0.35159 | 0.096057 | 0.012933 | 1.65997E-13 | Linoleic acid (LA) | 13523 |
| rs964184 | C | G | 0.856199 | -0.193421 | 0.01729 | 1.13999E-28 | Linoleic acid (LA) | 13525 |
| rs9804646 | T | C | 0.124469 | -0.105674 | 0.019115 | 4.05005E-08 | Linoleic acid (LA) | 13520 |
| rs99780 | T | C | 0.401006 | 0.148368 | 0.012472 | 3.34965E-32 | Linoleic acid (LA) | 13523 |
| rs190934192 | A | G | 0.027535 | -0.248268 | 0.043367 | 1.31999E-08 | Linoleic acid (LA) | 13523 |
| rs4609471 | A | C | 0.041643 | -0.253291 | 0.035446 | 1.29987E-12 | Linoleic acid (LA) | 13526 |
| rs821840 | G | A | 0.25211 | 0.083597 | 0.014639 | 1.43001E-08 | Linoleic acid (LA) | 13523 |
| rs2863979 | a | g | 0.7241 | 0.0143 | 0.0017 | 1.44E-17 | lysine | 7812 |
| rs320485 | t | c | 0.1324 | 0.0094 | 0.0017 | 4.97E-08 | methionine | 7795 |
| rs1718309 | G | A | 0.602584 | -0.077165 | 0.009719 | 2.50E-15 | Phenylalanine | 22660 |
| rs2731672 | C | T | 0.740037 | 0.094918 | 0.011618 | 3.85E-16 | Phenylalanine | 20436 |
| rs4253238 | T | C | 0.555072 | 0.067292 | 0.010053 | 2.53E-11 | Phenylalanine | 20435 |
| rs182695896 | C | A | 0.018066 | 0.245321 | 0.040112 | 1.09E-09 | Phenylalanine | 21467 |
| rs1016522 | A | G | 0.5803 | 0.0058 | 0.0009 | 1.59E-10 | tryptophan | 7804 |
| rs13122250 | T | C | 0.5542 | 0.0062 | 0.0009 | 8.95E-12 | tryptophan | 7804 |
| rs1373962 | T | C | 0.5973 | 0.005 | 0.0009 | 2.71E-08 | tryptophan | 7804 |
| rs1559063 | C | G | 0.6209 | 0.0052 | 0.0009 | 7.82E-09 | tryptophan | 7804 |
| rs2111118 | T | C | 0.6207 | 0.0051 | 0.0009 | 1.21E-08 | tryptophan | 7804 |
| rs284191 | A | G | 0.615 | -0.006 | 0.001 | 1.97E-09 | tryptophan | 7804 |
| rs38271 | A | G | 0.5925 | -0.0051 | 0.0009 | 1.19E-08 | tryptophan | 7804 |
| rs4306882 | T | G | 0.6153 | -0.0057 | 0.0009 | 2.52E-10 | tryptophan | 7804 |
| rs4615256 | A | G | 0.5327 | 0.0049 | 0.0009 | 4.99E-08 | tryptophan | 7804 |
| rs4695138 | A | T | 0.5352 | 0.0052 | 0.0009 | 8.00E-09 | tryptophan | 7804 |
| rs4958379 | A | G | 0.433 | -0.005 | 0.0009 | 2.18E-08 | tryptophan | 7804 |
| rs603446 | T | C | 0.4448 | 0.0051 | 0.0009 | 1.38E-08 | tryptophan | 7804 |
| rs6480970 | A | G | 0.6159 | -0.0049 | 0.0009 | 4.29E-08 | tryptophan | 7804 |
| rs6901004 | C | G | 0.5742 | -0.0061 | 0.0009 | 1.08E-11 | tryptophan | 7804 |
| rs6935961 | A | G | 0.5578 | -0.0056 | 0.0009 | 3.75E-10 | tryptophan | 7804 |
| rs710580 | A | C | 0.3566 | -0.005 | 0.0009 | 3.57E-08 | tryptophan | 7804 |
| rs7463805 | T | C | 0.5213 | -0.0053 | 0.0009 | 4.60E-09 | tryptophan | 7804 |
| rs7584842 | T | C | 0.5377 | -0.005 | 0.0009 | 4.15E-08 | tryptophan | 7804 |
| rs9511152 | A | G | 0.559 | -0.005 | 0.0009 | 2.99E-08 | tryptophan | 7804 |
| rs972459 | T | C | 0.5787 | 0.005 | 0.0009 | 1.97E-08 | tryptophan | 7804 |
| rs10211524 | A | G | 0.40991 | 0.086403 | 0.009427 | 5.24E-20 | Valine | 24898 |
| rs2072560 | C | T | 0.928539 | 0.104738 | 0.017688 | 3.28E-09 | Valine | 24895 |
| rs7406661 | C | T | 0.243378 | 0.079073 | 0.012727 | 5.35E-10 | Valine | 22659 |
| rs7655059 | G | C | 0.217667 | -0.068459 | 0.011163 | 8.91E-10 | Valine | 24897 |
| rs9637599 | C | A | 0.46958 | 0.113927 | 0.009156 | 1.67E-35 | Valine | 24899 |

Abbreviations: A1FREQ: A1 frequency; SE, standard error

# Supplementary Figures


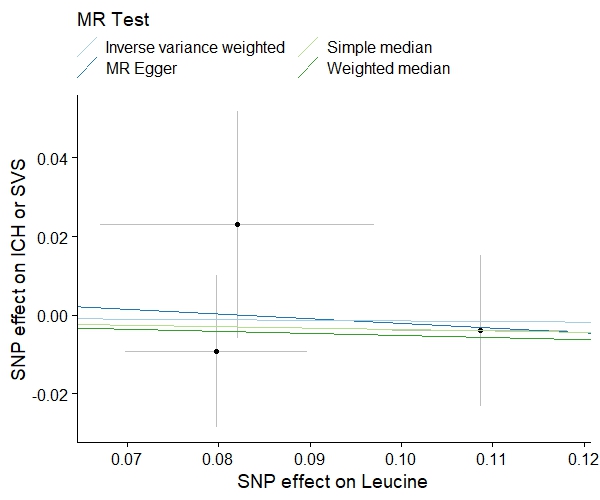


Supplementary Figure S1A. Scatterplot of leucine and ICH or SVS


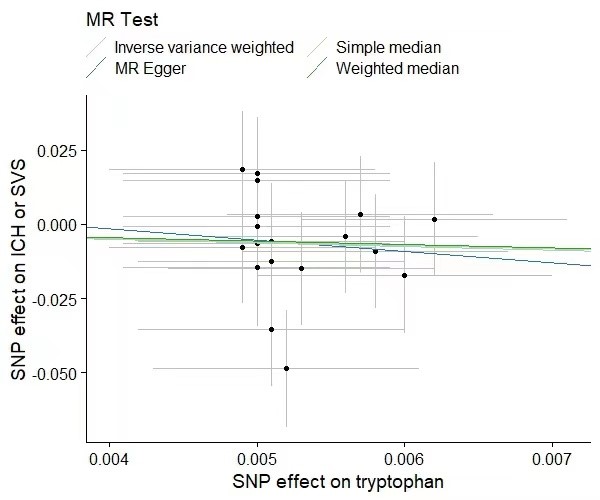


Supplementary Figure S1B. Scatterplot of tryptophan and ICH or SVS


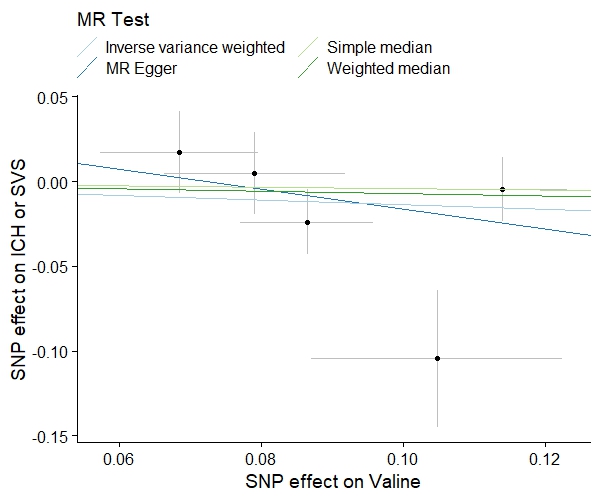


Supplementary Figure S1C. Scatterplot of valine and ICH or SVS


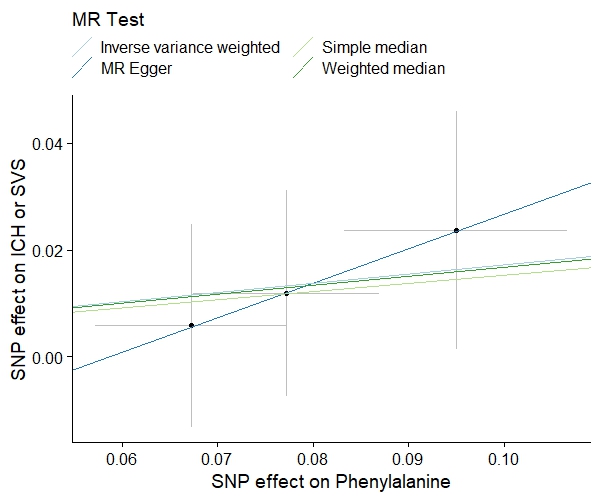


Supplementary Figure S1D. Scatterplot of phenylalanine and ICH or SVS


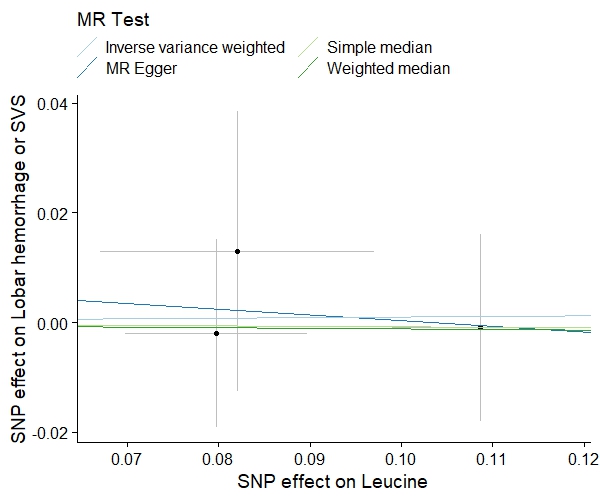


Supplementary Figure S1E. Scatterplot of leucine and lobar hemorrhage or SVS


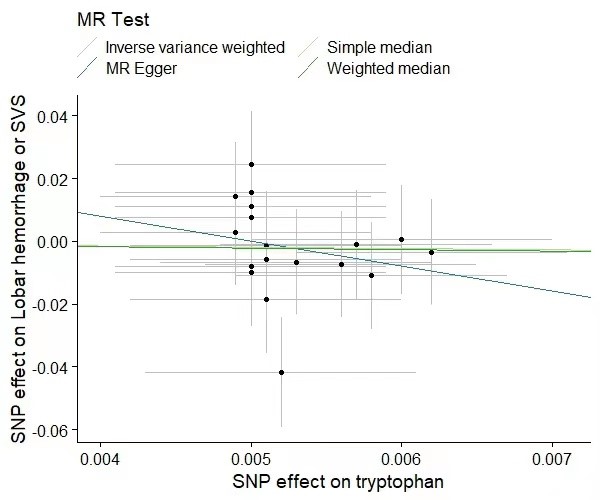


Supplementary Figure S1F. Scatterplot of tryptophan and lobar hemorrhage or SVS


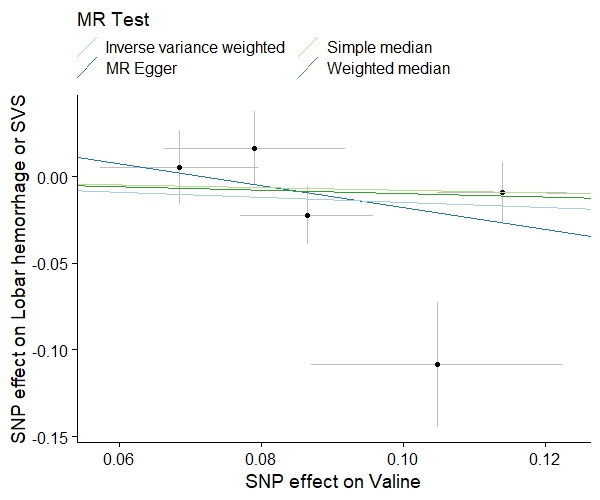


Supplementary Figure S1G. Scatterplot of valine and lobar hemorrhage or SVS


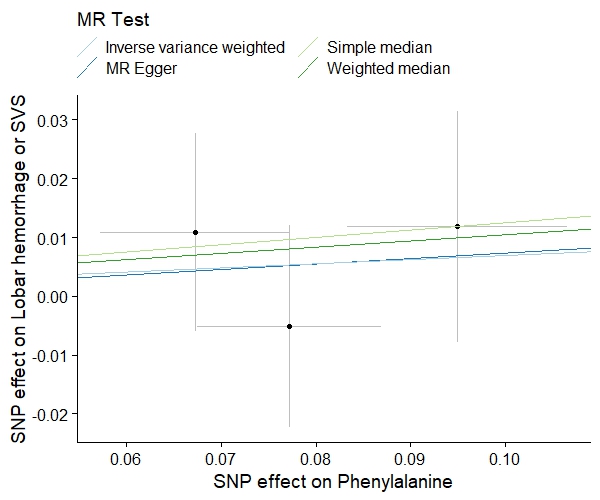


Supplementary Figure S1H. Scatterplot of phenylalanine and lobar hemorrhage or SVS


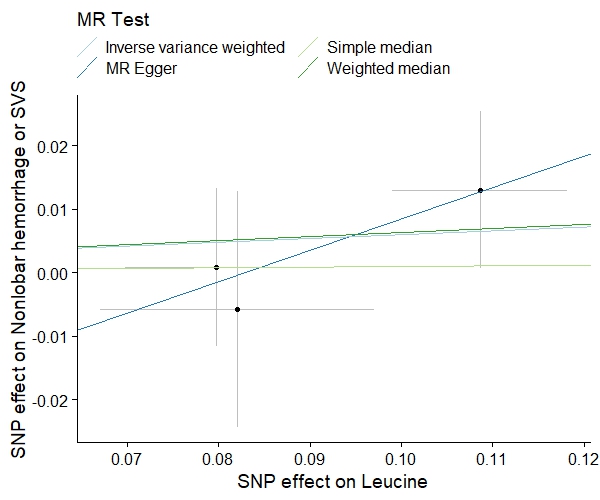


Supplementary Figure S1I. Scatterplot of leucine and nonlobar hemorrhage or SVS


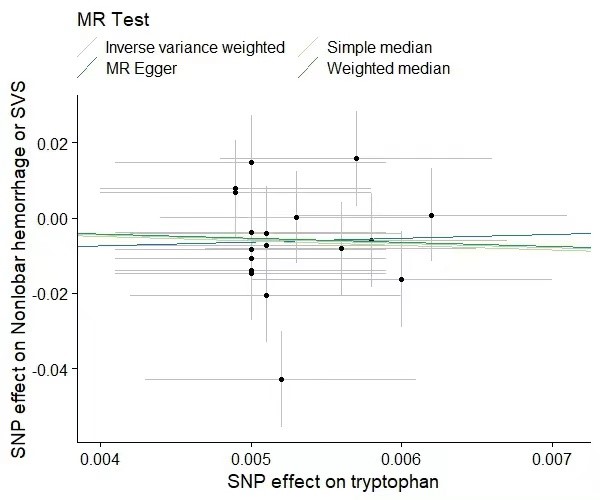


Supplementary Figure S1J. Scatterplot of tryptophan and nonlobar hemorrhage or SVS


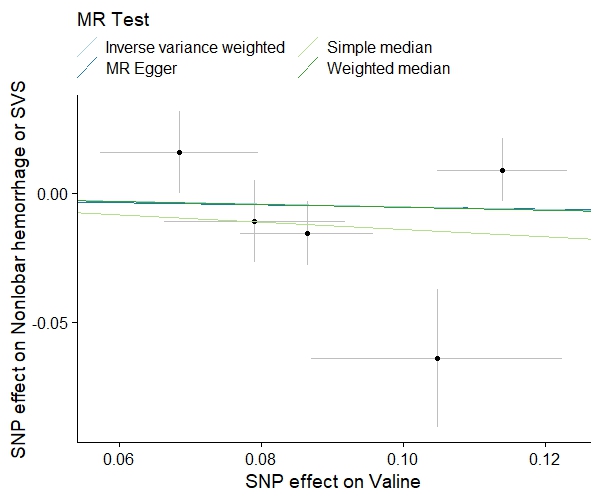


Supplementary Figure S1K. Scatterplot of valine and nonlobar hemorrhage or SVS


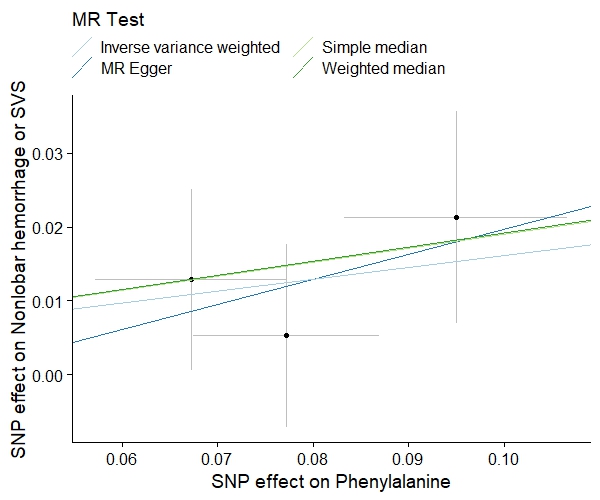


Supplementary Figure S1L. Scatterplot of phenylalanine and nonlobar hemorrhage or SVS

Supplementary Figure S1. Scatterplots of essential amino acids and cerebral hemorrhage or SVS. The x-axis represents the previously published β-estimate for the association between each SNP and essential nutrients. The y-axis represents the β-estimate for the association between each SNP and risk of cerebral hemorrhage. The slope of each line corresponds to the estimated MR effect per method.


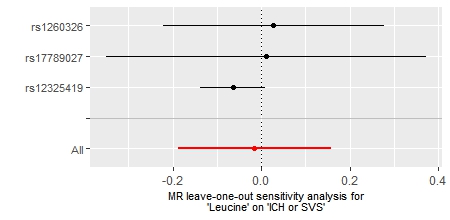


Supplementary Figure S2A. Leave-one-out plot of leucine and ICH or SVS


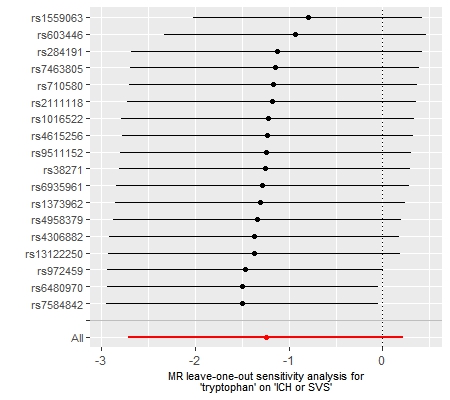


Supplementary Figure S2B. Leave-one-out plot of tryptophan and ICH or SVS


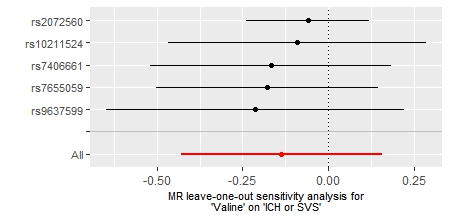


Supplementary Figure S2C. Leave-one-out plot of valine and ICH or SVS


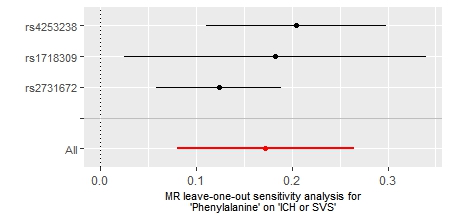


Supplementary Figure S2D. Leave-one-out plot of phenylalanine and ICH or SVS


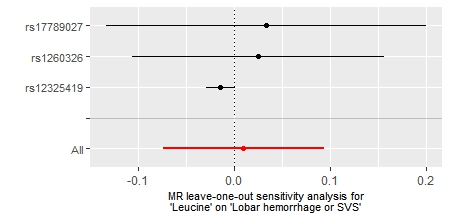


Supplementary Figure S2E. Leave-one-out plot of leucine and lobar hemorrhage or SVS


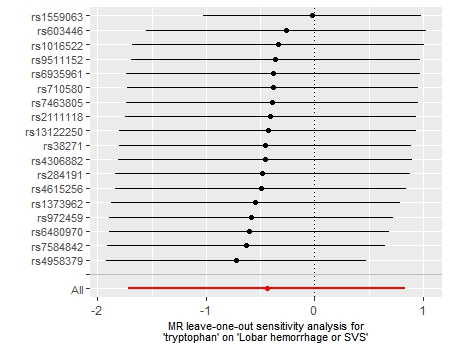


Supplementary Figure S2F. Leave-one-out plot of tryptophan and lobar hemorrhage or SVS


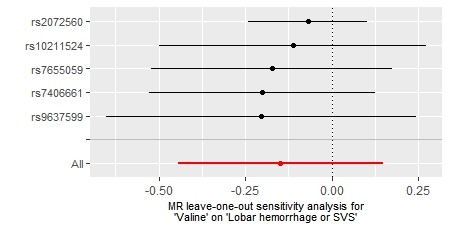


Supplementary Figure S2G. Leave-one-out plot of valine and lobar hemorrhage or SVS


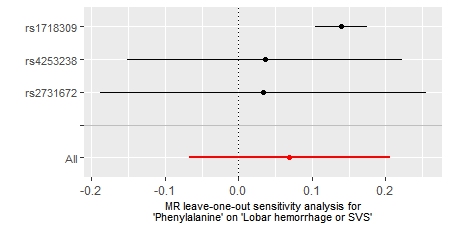


Supplementary Figure S2H. Leave-one-out plot of phenylalanine and lobar hemorrhage or SVS


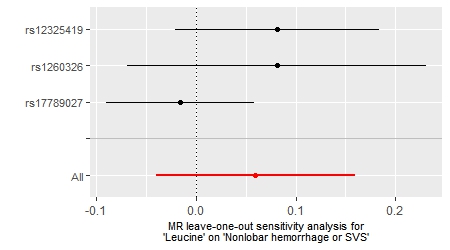


Supplementary Figure S2I. Leave-one-out plot of leucine and nonlobar hemorrhage or SVS


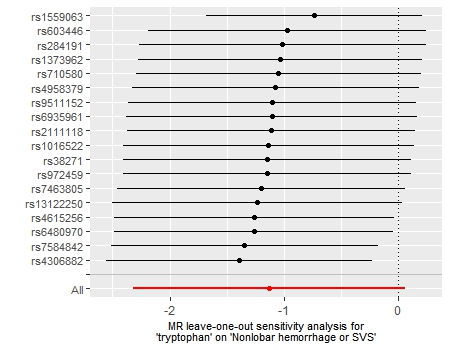


Supplementary Figure S2J. Leave-one-out plot of tryptophan and nonlobar hemorrhage or SVS


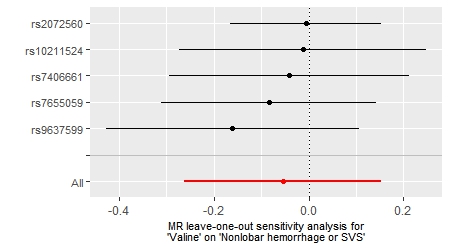


Supplementary Figure S2K. Leave-one-out plot of valine and nonlobar hemorrhage or SVS


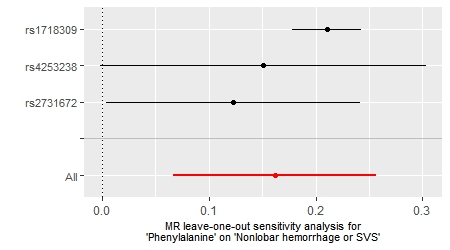


Supplementary Figure S2L. Leave-one-out plot of phenylalanine and nonlobar hemorrhage or SVS

Supplementary Figure S2. Leave-one-out plots of essential amino acids and cerebral hemorrhage or SVS. The x-axis represents the beta value for the outcome obtained by removing the left SNP from the IVW analysis (i.e., the dots on each solid line). The y-axis represents the SNP removed for each analysis. Each solid line represents the 95% CI for the beta value. The bottom red line is the overall result obtained by all the SNPs of exposure.


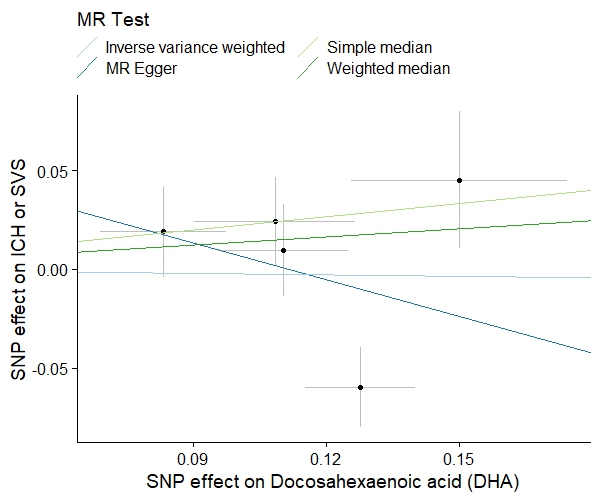


Supplementary Figure S3A. Scatterplot of docosahexaenoic acid (DHA) and ICH or SVS


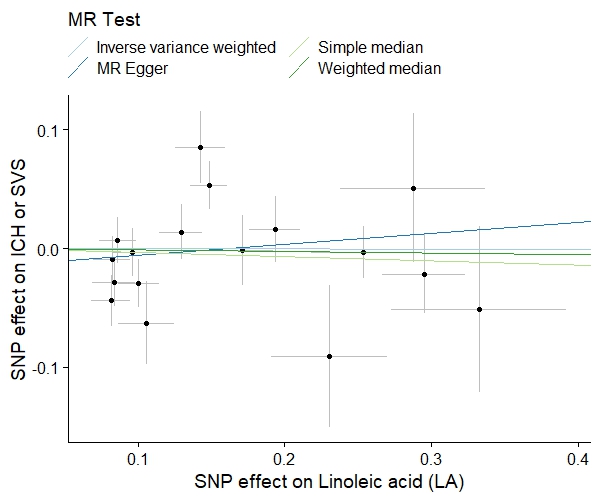


Supplementary Figure S3B. Scatterplot of linoleic acid (LA) and ICH or SVS


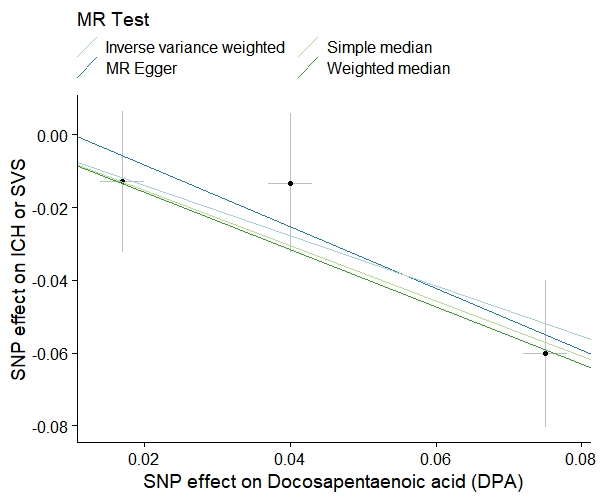


Supplementary Figure S3C. Scatterplot of docosapentaenoic acid (DPA) and ICH or SVS


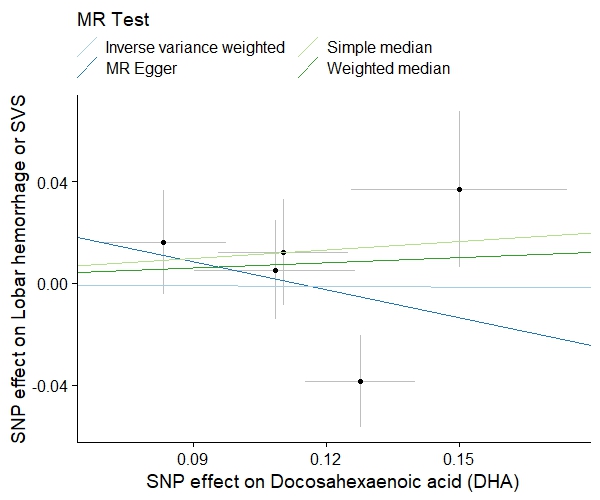


Supplementary Figure S3D. Scatterplot of docosahexaenoic acid (DHA) and lobar hemorrhage or SVS


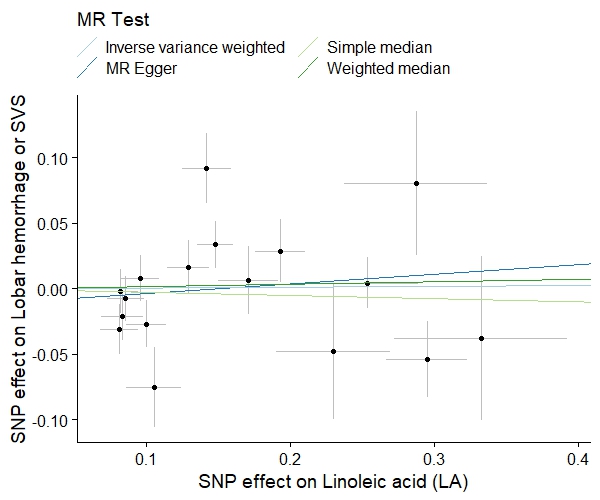


Supplementary Figure S3E. Scatterplot of linoleic acid (LA) and lobar hemorrhage or SVS


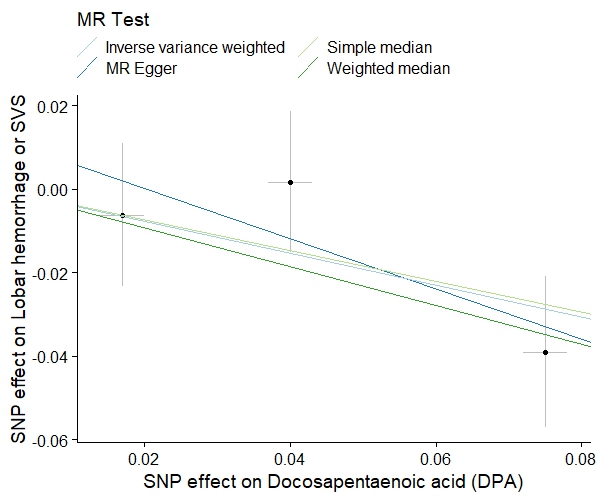


Supplementary Figure S3F. Scatterplot of docosapentaenoic acid (DPA) and lobar hemorrhage or SVS


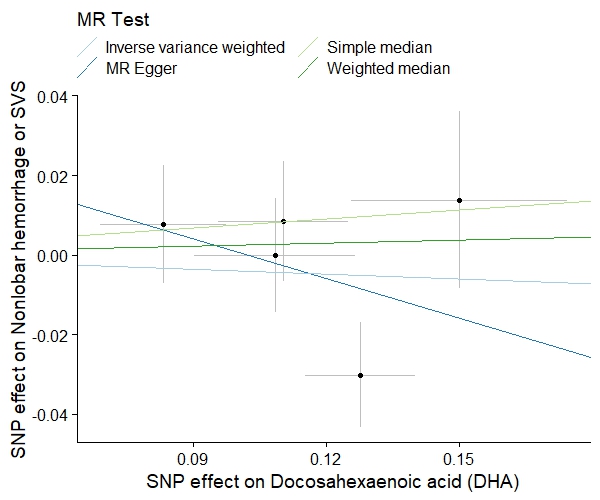


Supplementary Figure S3G. Scatterplot of docosahexaenoic acid (DHA) and nonlobar hemorrhage or SVS


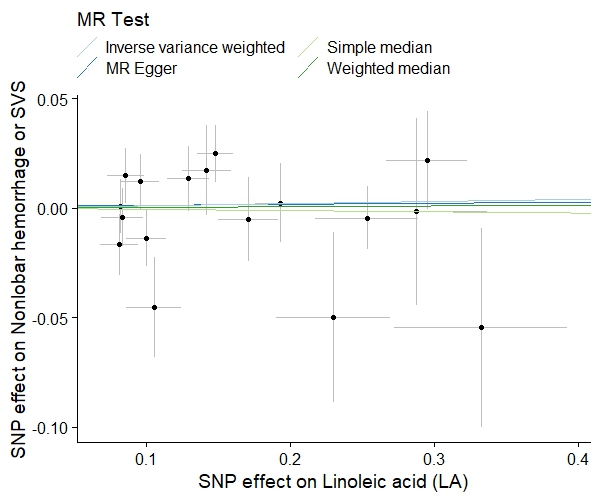


Supplementary Figure S3H. Scatterplot of linoleic acid (LA) and nonlobar hemorrhage or SVS


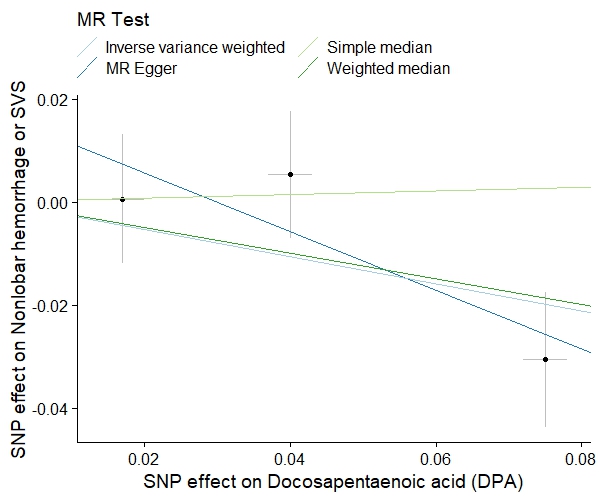


Supplementary Figure S3I. Scatterplot of docosapentaenoic acid (DPA) and nonlobar hemorrhage or SVS

Supplementary Figure S3. Scatterplots of essential polyunsaturated fatty acids and cerebral hemorrhage or SVS. The x-axis represents the previously published β-estimate for the association between each SNP and essential nutrients. The y-axis represents the β-estimate for the association between each SNP and risk of cerebral hemorrhage. The slope of each line corresponds to the estimated MR effect per method.


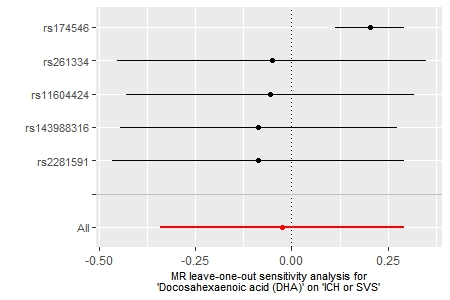


Supplementary Figure S4A. Leave-one-out plot of docosahexaenoic acid (DHA) and ICH or SVS


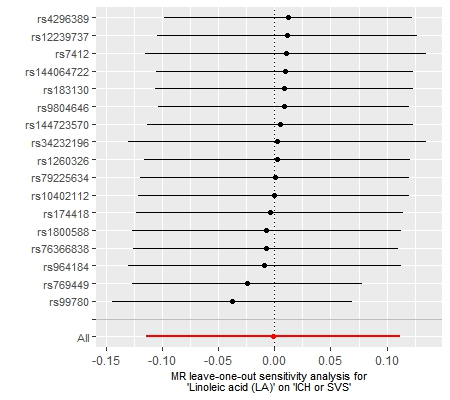


Supplementary Figure S4B. Leave-one-out plot of linoleic acid (LA) and ICH or SVS


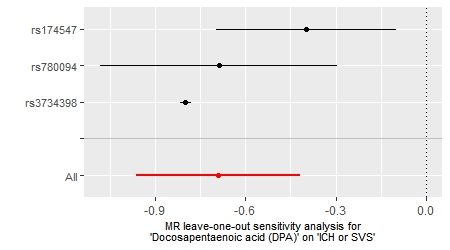


Supplementary Figure S4C. Leave-one-out plot of docosapentaenoic acid (DPA) and ICH or SVS


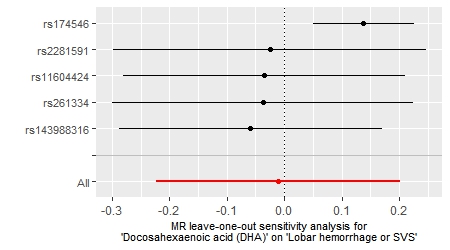


Supplementary Figure S4D. Leave-one-out plot of docosahexaenoic acid (DHA) and lobar hemorrhage or SVS


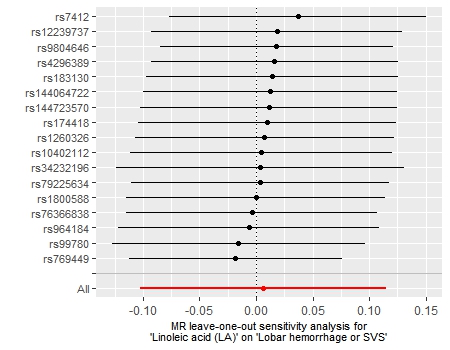


Supplementary Figure S4E. Leave-one-out plot of linoleic acid (LA) and lobar hemorrhage or SVS


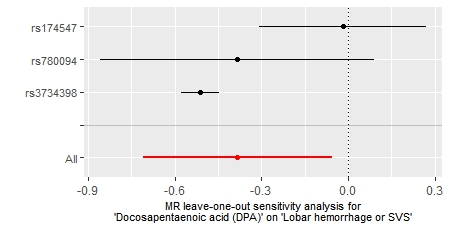


Supplementary Figure S4F. Leave-one-out plot of docosapentaenoic acid (DPA) and lobar hemorrhage or SVS


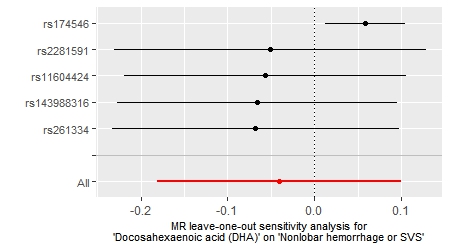


Supplementary Figure S4G. Leave-one-out plot of docosahexaenoic acid (DHA) and nonlobar hemorrhage or SVS


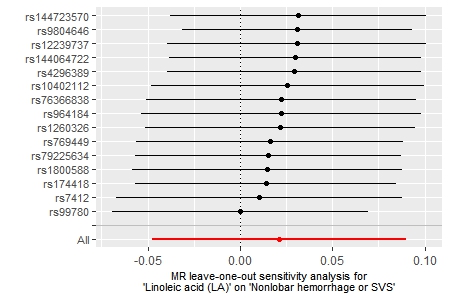


Supplementary Figure S4H. Leave-one-out plot of linoleic acid (LA) and nonlobar hemorrhage or SVS


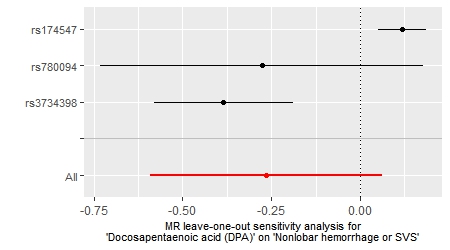


Supplementary Figure S4I. Leave-one-out plot of docosapentaenoic acid (DPA) and nonlobar hemorrhage or SVS

Supplementary Figure S4. Leave-one-out plots of essential polyunsaturated fatty acids and cerebral hemorrhage or SVS. The x-axis represents the beta value for the outcome obtained by removing the left SNP from the IVW analysis (i.e., the dots on each solid line). The y-axis represents the SNP removed for each analysis. Each solid line represents the 95% CI for the beta value. The bottom red line is the overall result obtained by all the SNPs of exposure.


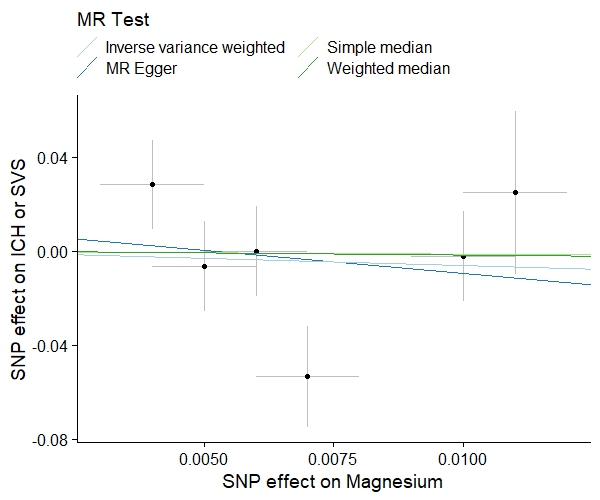


Supplementary Figure S5A. Scatterplot of magnesium and ICH or SVS


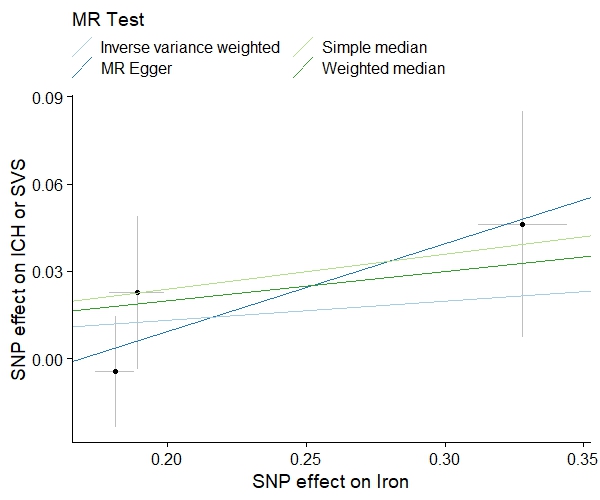


Supplementary Figure S5B. Scatterplot of iron and ICH or SVS


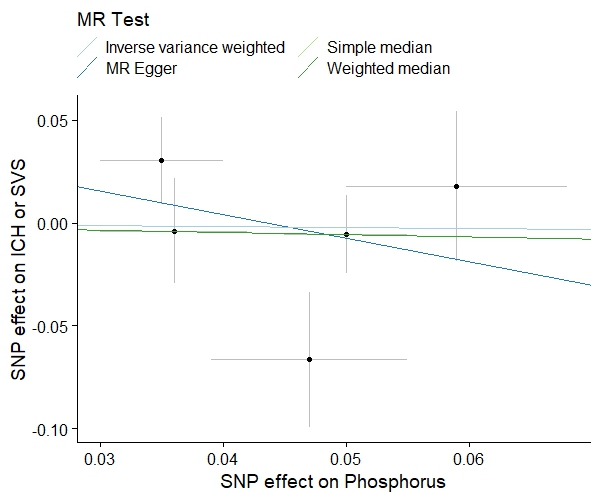


Supplementary Figure S5C. Scatterplot of phosphorus and ICH or SVS


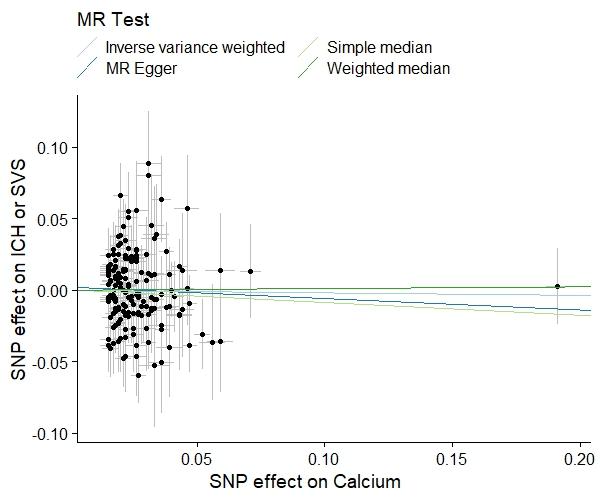


Supplementary Figure S5D. Scatterplot of calcium and ICH or SVS


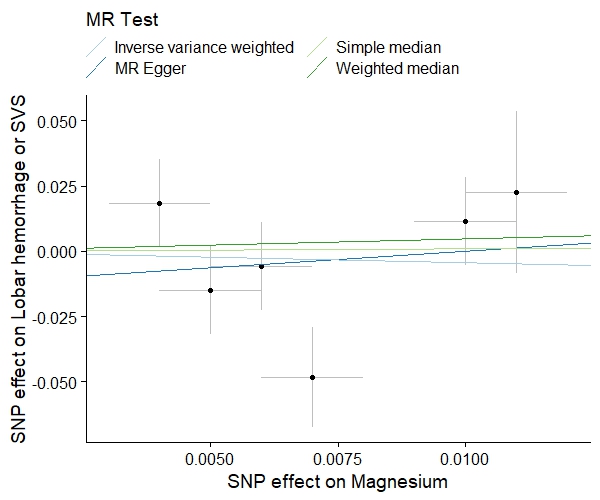


Supplementary Figure S5E. Scatterplot of magnesium and lobar hemorrhage or SVS


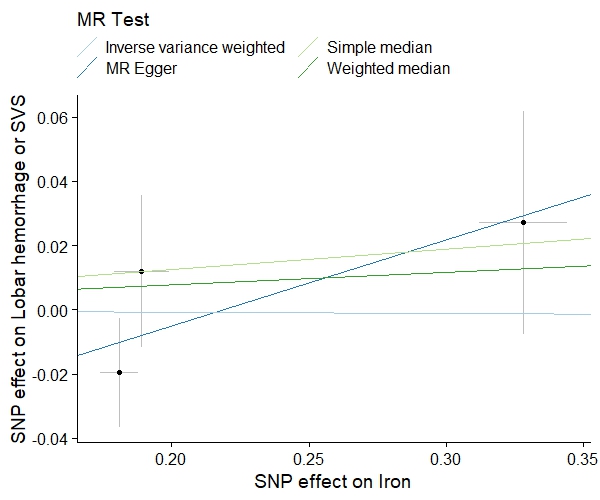


Supplementary Figure S5F. Scatterplot of iron and lobar hemorrhage or SVS


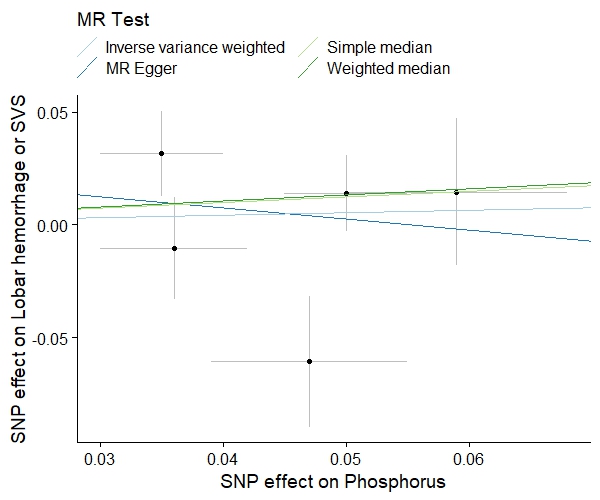


Supplementary Figure S5G. Scatterplot of phosphorus and lobar hemorrhage or SVS


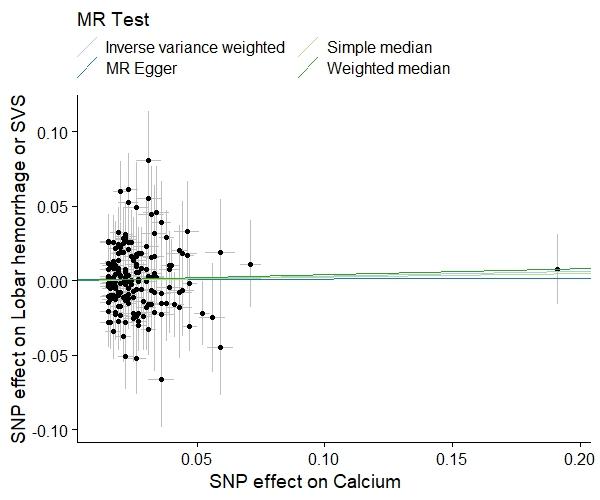


Supplementary Figure S5H. Scatterplot of calcium and lobar hemorrhage or SVS


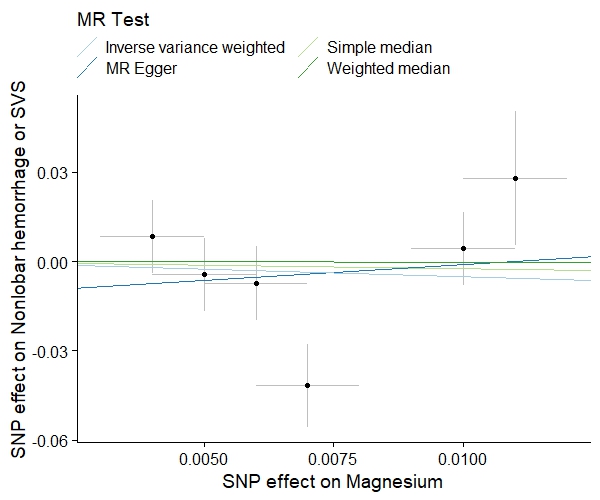


Supplementary Figure S5I. Scatterplot of magnesium and nonlobar hemorrhage or SVS


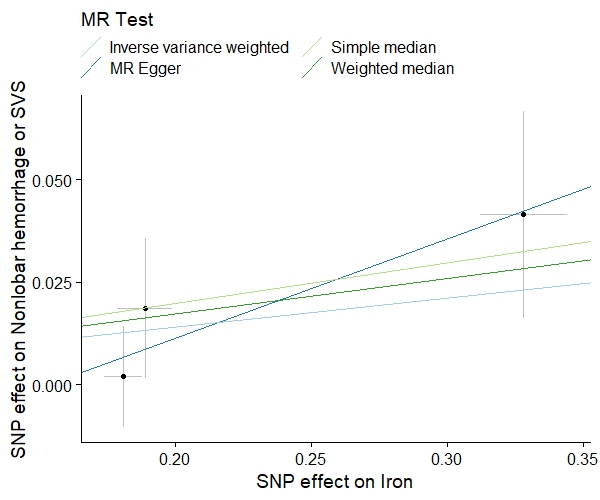


Supplementary Figure S5J. Scatterplot of iron and nonlobar hemorrhage or SVS


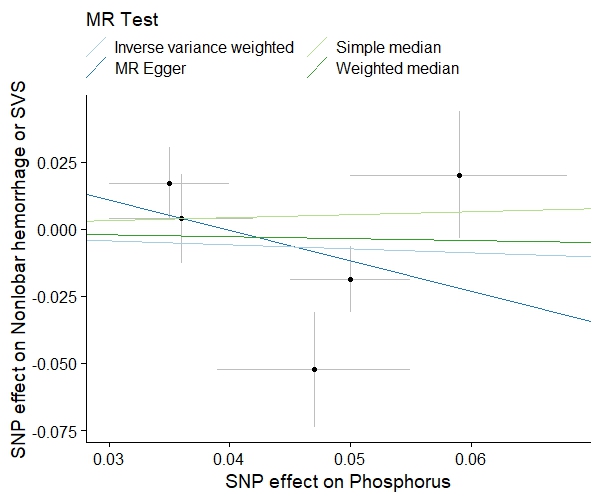


Supplementary Figure S5K. Scatterplot of phosphorus and nonlobar hemorrhage or SVS


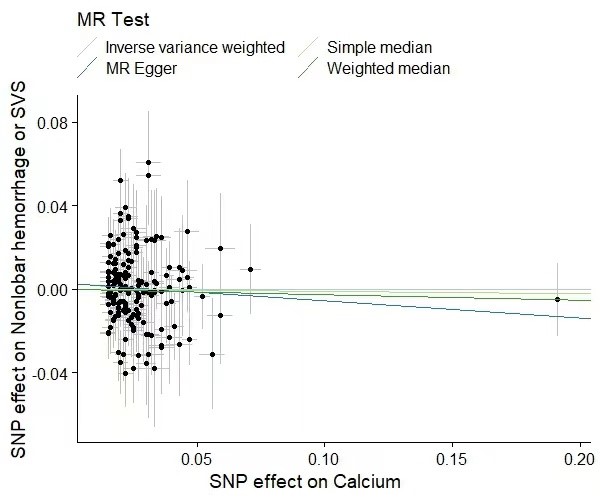


Supplementary Figure S5L. Scatterplot of calcium and nonlobar hemorrhage or SVS

Supplementary Figure S5. Scatterplots of essential minerals and cerebral hemorrhage or SVS. The x-axis represents the previously published β-estimate for the association between each SNP and essential nutrients. The y-axis represents the β-estimate for the association between each SNP and risk of cerebral hemorrhage. The slope of each line corresponds to the estimated MR effect per method.


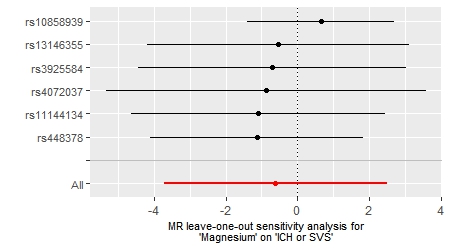


Supplementary Figure S6A. Leave-one-out plot of magnesium and ICH or SVS


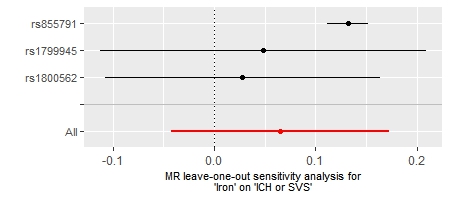


Supplementary Figure S6B. Leave-one-out plot of iron and ICH or SVS


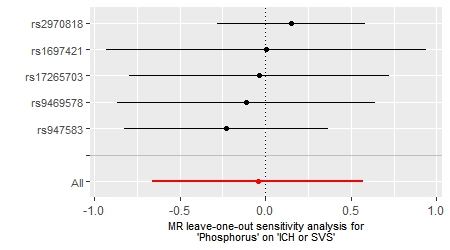


Supplementary Figure S6C. Leave-one-out plot of phosphorus and ICH or SVS


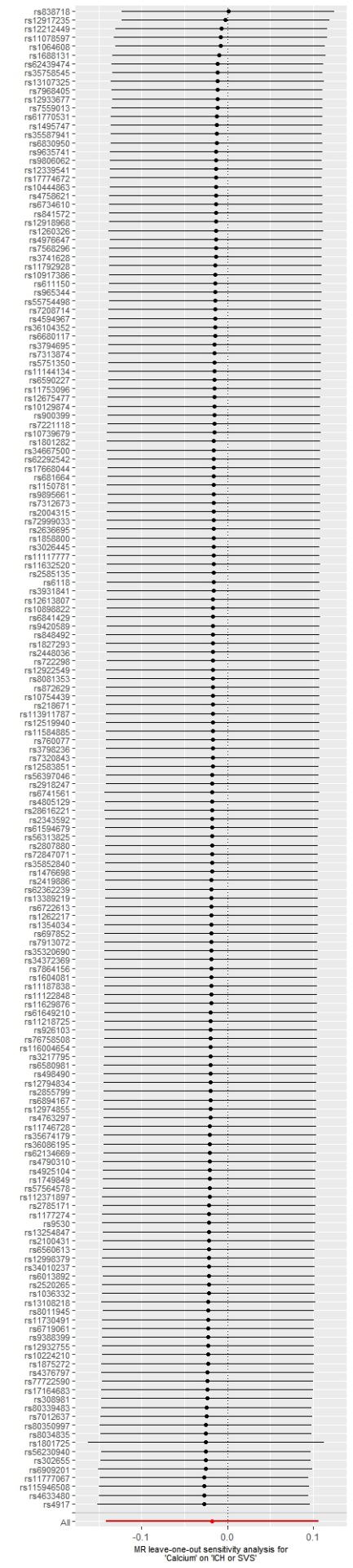


Supplementary Figure S6D. Leave-one-out plot of calcium and ICH or SVS


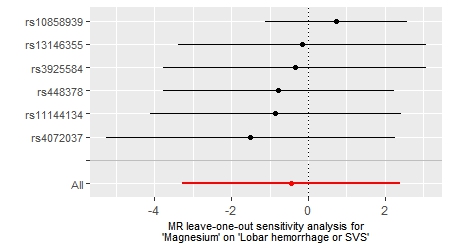


Supplementary Figure S6E. Leave-one-out plot of magnesium and lobar hemorrhage or SVS


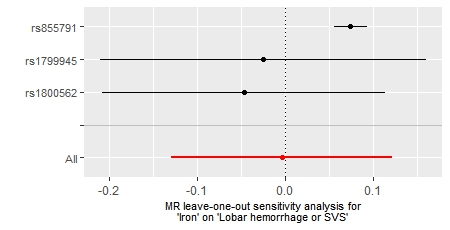


Supplementary Figure S6F. Leave-one-out plot of iron and lobar hemorrhage or SVS


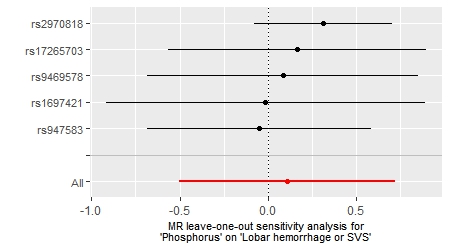


Supplementary Figure S6G. Leave-one-out plot of phosphorus and lobar hemorrhage or SVS


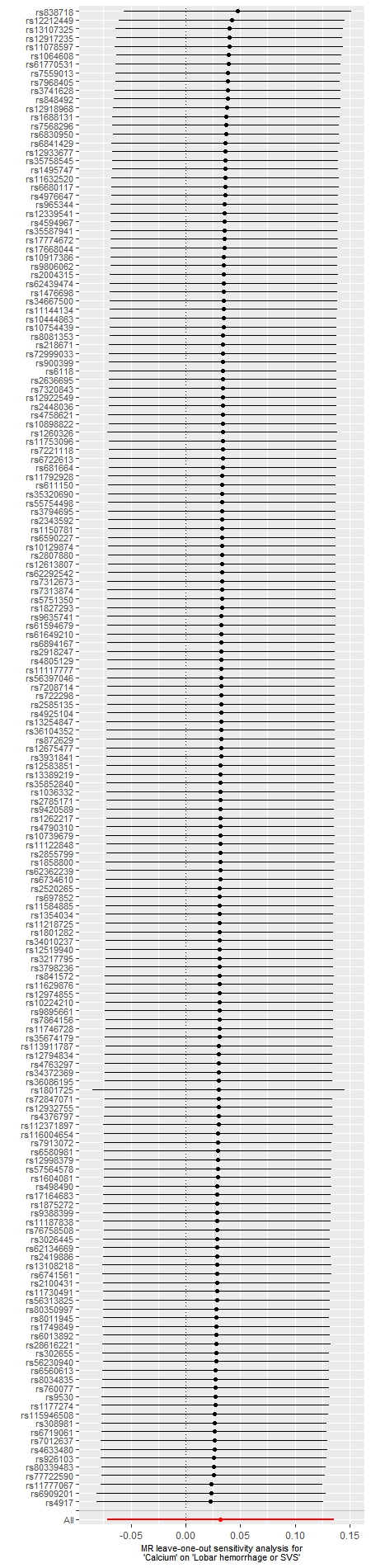


Supplementary Figure S6H. Leave-one-out plot of calcium and lobar hemorrhage or SVS


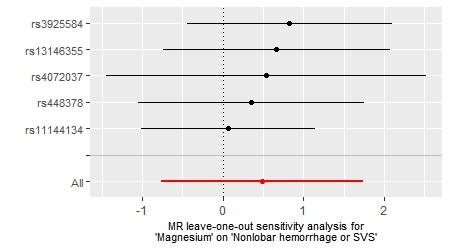


Supplementary Figure S3I. Leave-one-out plot of magnesium and nonlobar hemorrhage or SVS


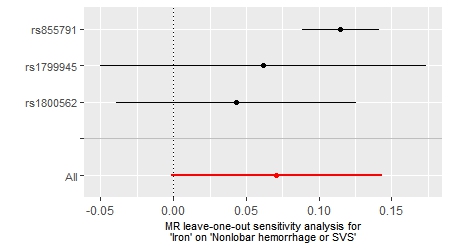


Supplementary Figure S6J. Leave-one-out plot of iron and nonlobar hemorrhage or SVS


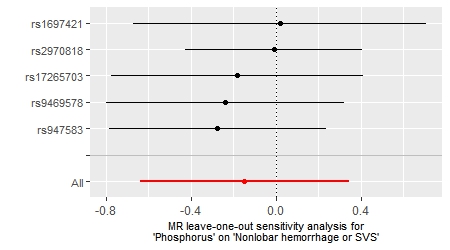


Supplementary Figure S6K. Leave-one-out plot of phosphorus and nonlobar hemorrhage or SVS


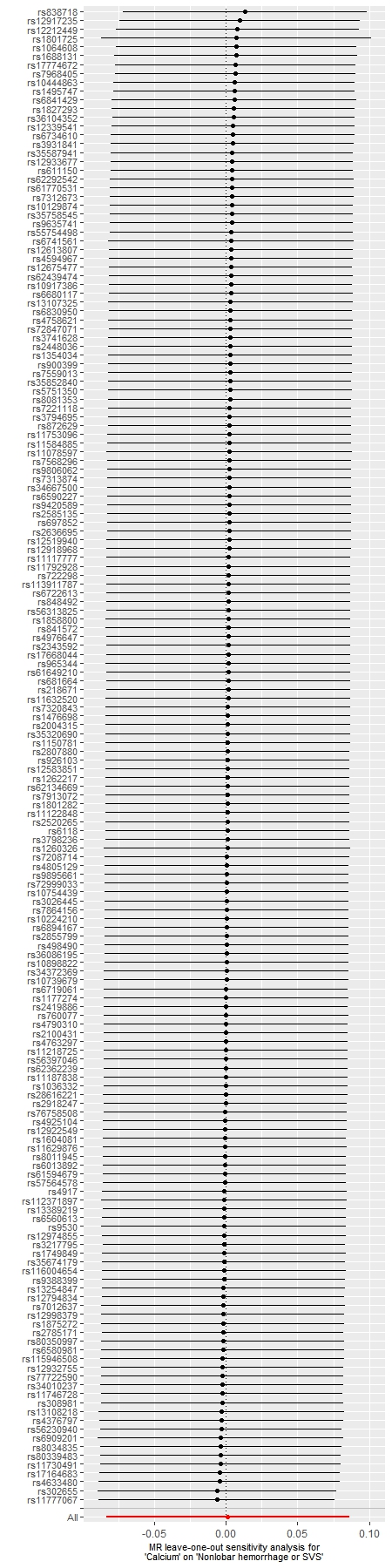


Supplementary Figure S6L. Leave-one-out plot of calcium and nonlobar hemorrhage or SVS

Supplementary Figure S6. Leave-one-out plots of essential minerals and cerebral hemorrhage or SVS. The x-axis represents the beta value for the outcome obtained by removing the left SNP from the IVW analysis (i.e., the dots on each solid line). The y-axis represents the SNP removed for each analysis. Each solid line represents the 95% CI for the beta value. The bottom red line is the overall result obtained by all the SNPs of exposure.


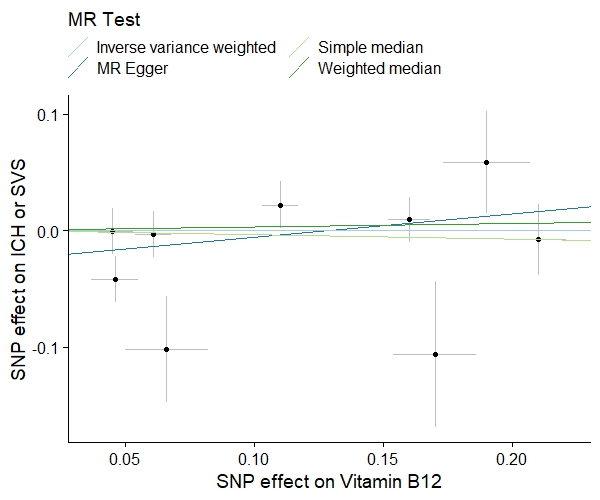


Supplementary Figure S7A. Scatterplot of vitamin B12 and ICH or SVS


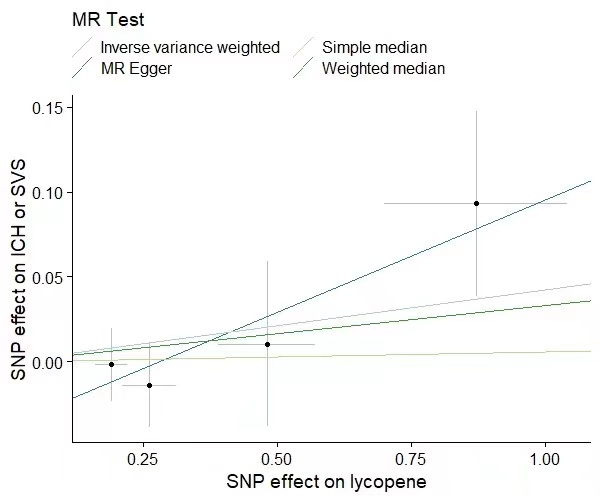


Supplementary Figure S7B. Scatterplot of lycopene and ICH or SVS


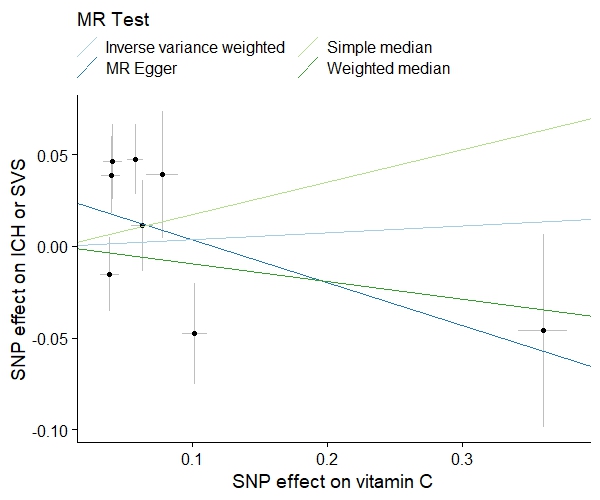


Supplementary Figure S7C. Scatterplot of vitamin C and ICH or SVS


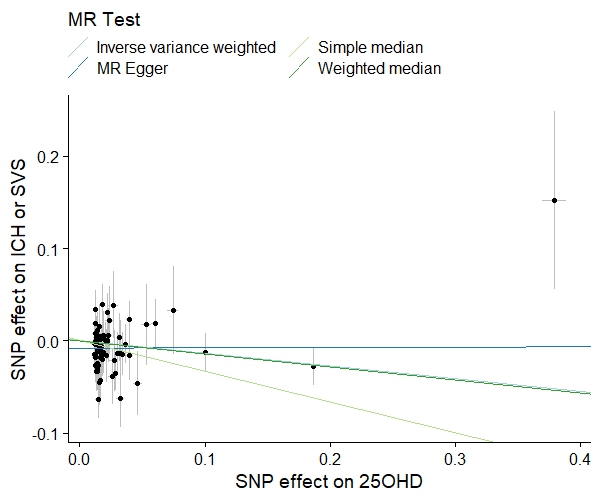


Supplementary Figure S7D. Scatterplot of 25(OH)D and ICH or SVS


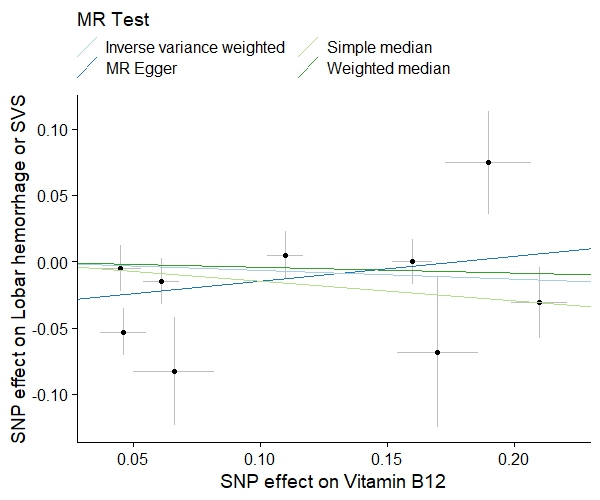


Supplementary Figure S7E. Scatterplot of vitamin B12 and lobar hemorrhage or SVS


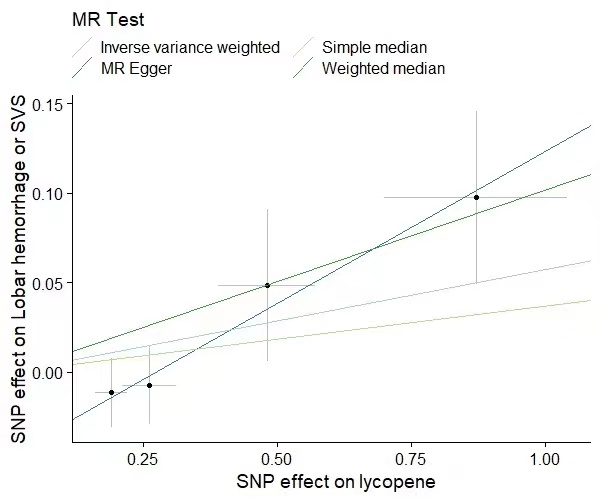


Supplementary Figure S7F. Scatterplot of lycopene and lobar hemorrhage or SVS


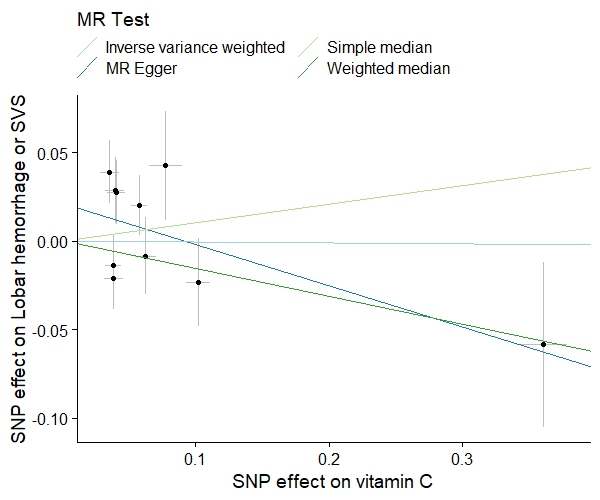


Supplementary Figure S7G. Scatterplot of vitamin C and lobar hemorrhage or SVS


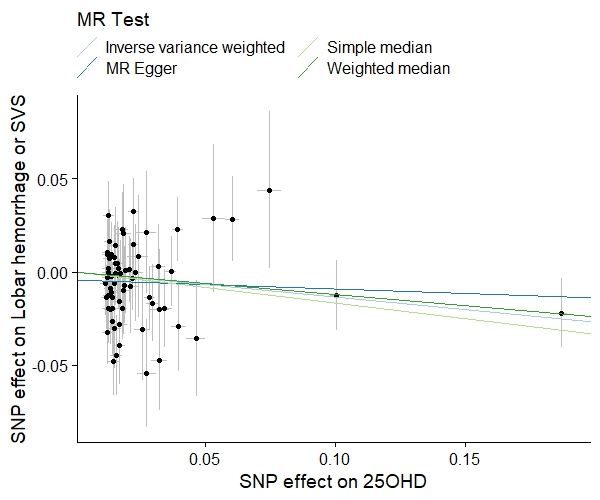


Supplementary Figure S7H. Scatterplot of 25(OH)D and lobar hemorrhage or SVS


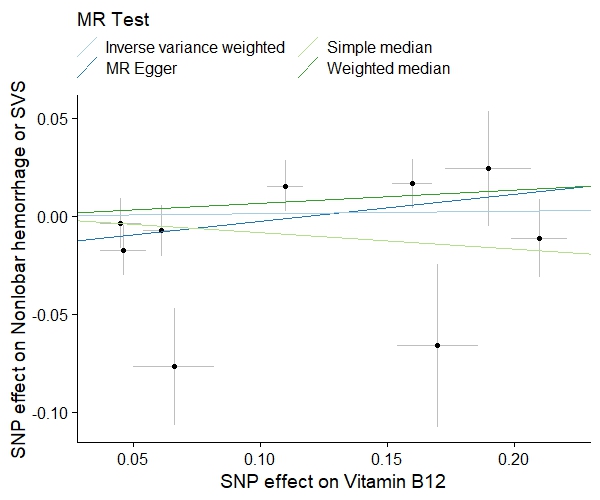


Supplementary Figure S7I. Scatterplot of vitamin B12 and nonlobar hemorrhage or SVS


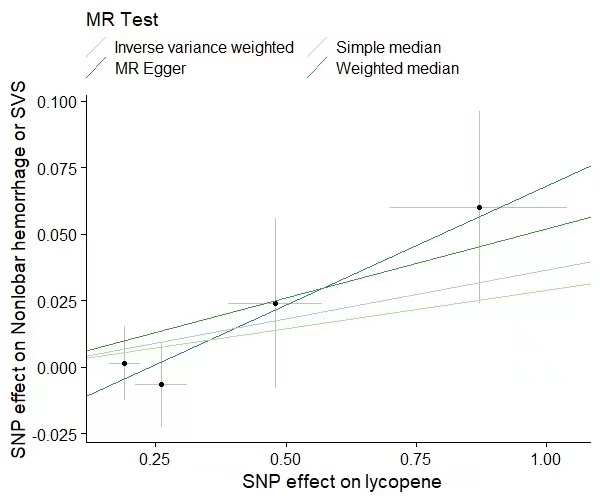


Supplementary Figure S7J. Scatterplot of lycopene and nonlobar hemorrhage or SVS


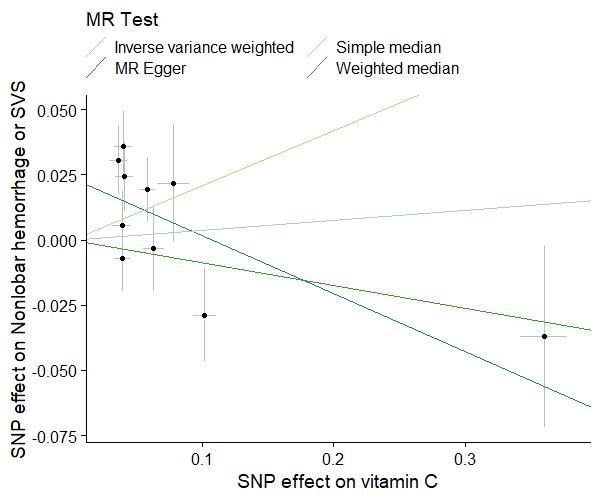


Supplementary Figure S7K. Scatterplot of vitamin C and nonlobar hemorrhage or SVS


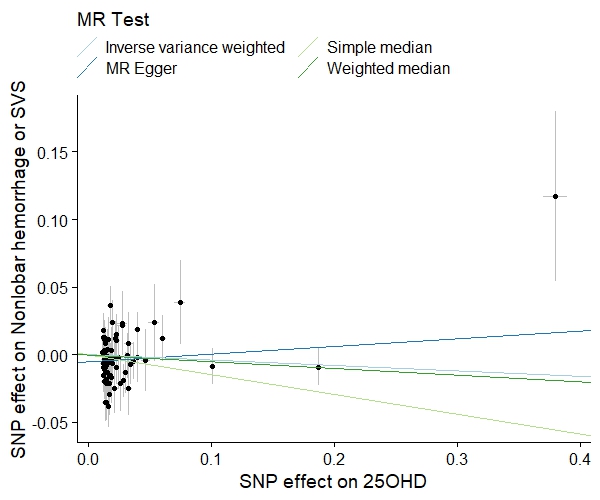


Supplementary Figure S7L. Scatterplot of 25(OH)D and nonlobar hemorrhage or SVS

Supplementary Figure S7. Scatterplots of essential vitamins and cerebral hemorrhage or SVS. The x-axis represents the previously published β-estimate for the association between each SNP and essential nutrients. The y-axis represents the β-estimate for the association between each SNP and risk of cerebral hemorrhage. The slope of each line corresponds to the estimated MR effect per method.


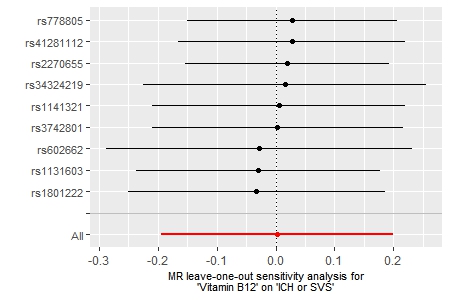


Supplementary Figure S8A. Leave-one-out plot of vitamin B12 and ICH or SVS


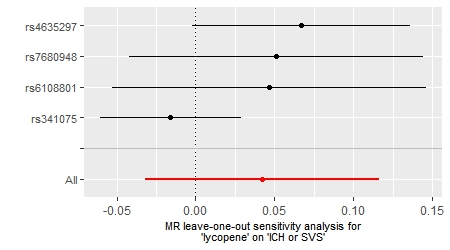


Supplementary Figure S8B. Leave-one-out plot of lycopene and ICH or SVS


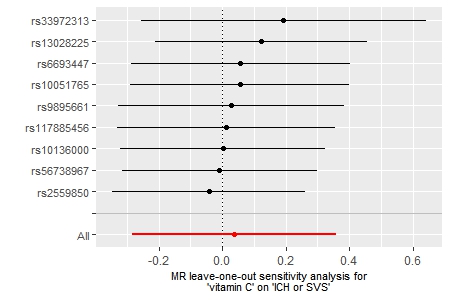


Supplementary Figure S8C. Leave-one-out plot of vitamin C and ICH or SVS


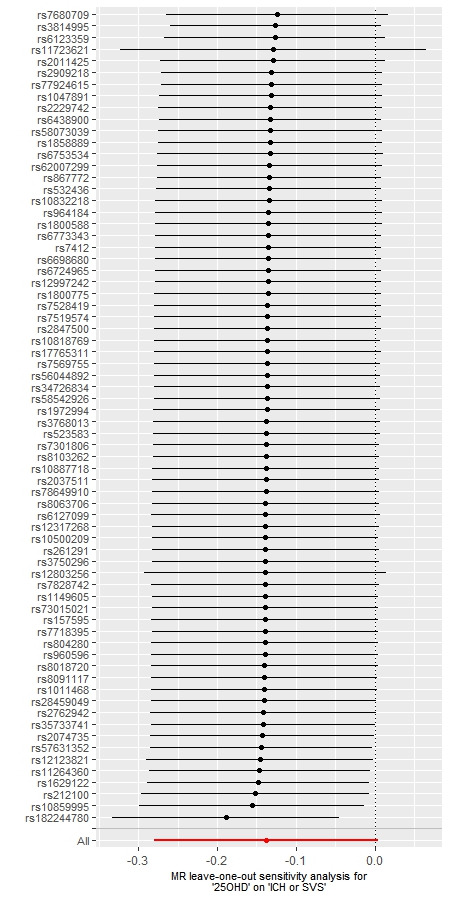


Supplementary Figure S8D. Leave-one-out plot of 25(OH)D and ICH or SVS


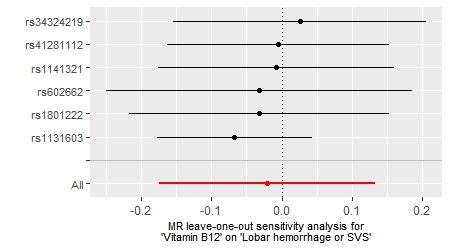


Supplementary Figure S8E. Leave-one-out plot of vitamin B12 and lobar hemorrhage or SVS


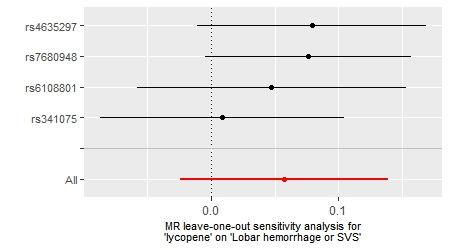


Supplementary Figure S8F. Leave-one-out plot of lycopene and lobar hemorrhage or SVS


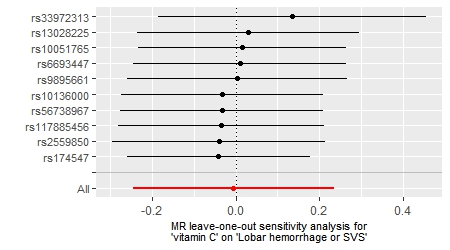


Supplementary Figure S8G. Leave-one-out plot of vitamin C and lobar hemorrhage or SVS


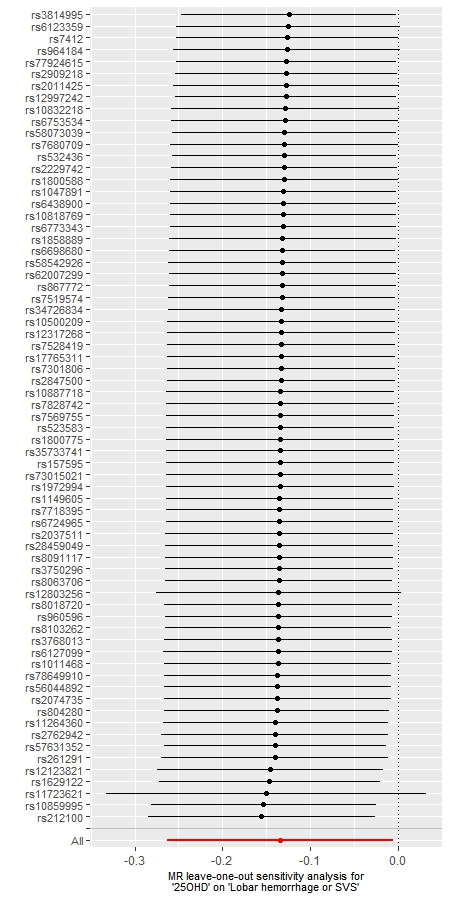


Supplementary Figure S8H. Leave-one-out plot of 25(OH)D and lobar hemorrhage or SVS


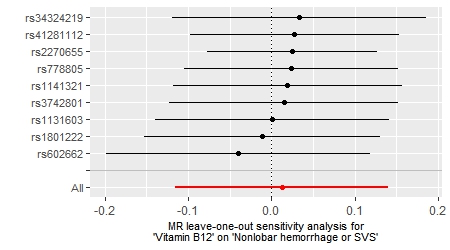


Supplementary Figure S8I. Leave-one-out plot of vitamin B12 and nonlobar hemorrhage or SVS


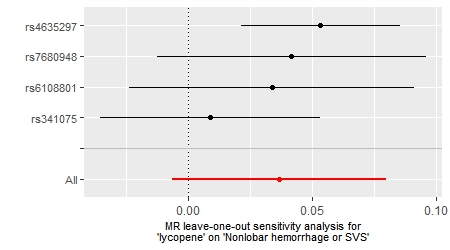


Supplementary Figure S3J. Leave-one-out plot of lycopene and nonlobar hemorrhage or SVS


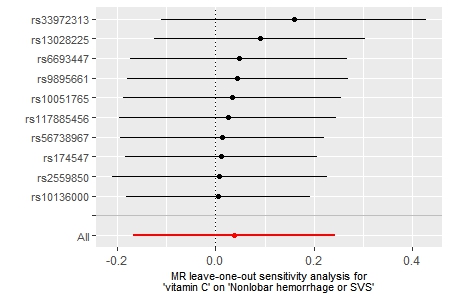


Supplementary Figure S8K. Leave-one-out plot of vitamin C and nonlobar hemorrhage or SVS


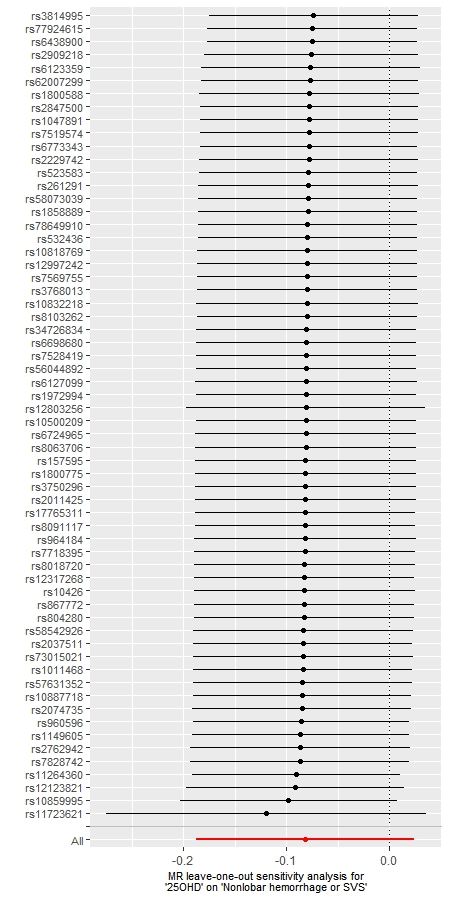


Supplementary Figure S8L. Leave-one-out plot of 25(OH)D and nonlobar hemorrhage or SVS

Supplementary Figure S8. Leave-one-out plots of essential vitamins and cerebral hemorrhage or SVS. The x-axis represents the beta value for the outcome obtained by removing the left SNP from the IVW analysis (i.e., the dots on each solid line). The y-axis represents the SNP removed for each analysis. Each solid line represents the 95% CI for the beta value. The bottom red line is the overall result obtained by all the SNPs of exposure.
